# Macroalgal community transformation during successive marine heatwaves in southern California kelp forests

**DOI:** 10.1038/s43247-026-03599-5

**Published:** 2026-05-16

**Authors:** Kristen M. Michaud, Daniel C. Reed, Robert J. Miller

**Affiliations:** Marine Science Institute, University of California Santa Barbara, Santa Barbara, CA USA

**Keywords:** Marine biology, Environmental impact, Climate-change ecology, Community ecology

## Abstract

Understanding the acute and longer-term effects of marine heatwaves on coastal ecosystems is critical for predicting their response to future warming. We used a 23-year time series that included two marine heatwaves (the 2014–2015 “warm blob” and 2018) to investigate their effects on understory macroalgae at nine rocky reefs with giant kelp forests in the Santa Barbara Channel monitored by the Santa Barbara Coastal Long Term Ecological Research program. We observed little change in macroalgal biomass or species richness in response to the heatwaves, but significant changes in community composition, including the loss of the dominant understory kelp *Pterygophora californica* and shifts towards subtropical and introduced species. Understory communities overall were surprisingly resilient, as community composition largely returned to pre-heatwave conditions within a few years. However, there were exceptions. In particular, subtropical species and geniculate coralline algae increased in abundance in the decade following the heatwaves, suggesting that macroalgal communities are likely to shift in the long term if marine heatwaves increase in frequency and intensity.

## Abstract (web summary)

Marine heatwaves induce significant changes in the community composition of kelp forests, including loss of understory kelp and shifts towards subtropical/introduced species, according to analyses of temperature data and ecological transects from southern California.

## Introduction

Temperature is fundamentally important in determining the distribution and diversity of marine organisms^1–4^ over ecological and evolutionary time scales^5^. As global ocean temperature has significantly increased over the last century^6,7^, many species have shifted both poleward and deeper to remain within their thermal tolerance limits^8–10^. In conjunction with this longer-term trend, acute warming events (i.e., marine heatwaves) can cause abrupt and drastic shifts in marine communities^11,12^. Increases in mean ocean temperatures combined with regionally-specific climate drivers, including ocean-atmospheric processes and ENSO dynamics in the Pacific, have coincided with increased frequency and intensity of marine heatwaves over the last century^13,14^. These extreme events are projected to become more frequent in many regions under future warming scenarios^14,15^, potentially impacting marine ecosystems and the goods and services they provide.

The ecological and socioeconomic consequences of marine heatwaves can be severe, threatening biodiversity and altering ecosystem structure and function^11,12,16,17^. Such impacts can be driven by both range expansions and species losses^10,18–20^. Increases in warm-tolerant invasive species have caused significant changes to community composition following heatwave disturbances^21,22^. By contrast, declines of fish species during periods of acute warming have caused substantial losses in commercial and recreational fisheries^23,24^. Marine heatwaves can be particularly damaging when extended warming threatens coastal foundation species such as kelps, seagrasses, mangroves, and hermatypic corals, which increase habitat complexity, biodiversity, productivity, and ecosystem stability^11,12^. For example, mass coral bleaching events have been accompanied by functional homogenization of fish communities as coral-associated species are lost^25^. Such shifts in species abundances may subsequently alter interspecific interactions, with unexpected consequences for marine communities^26^.

Shallow temperate reefs dominated by macroalgae are highly productive ecosystems, hosting a diverse suite of species that provide important ecosystem goods and services to coastal communities^27–29^. The response of macroalgae to marine heatwaves varies among species and functional groups; some are resistant, while others, including many large canopy-forming kelps, display decreased physiological performance or abundance declines^30^. Differences in life history or thermal tolerance may confer advantages to some species and functional groups, but whether such traits can be used to reliably predict shifts in benthic community structure is unclear. For example, geniculate coralline algae (GCA) showed poor thermal tolerance to simulated heatwaves in the laboratory^31^, yet GCA increased during strong El Niño years^32,33^, suggesting that this group may benefit from warmer conditions. Species-specific responses can also vary over both time and space^34–36^, making it difficult to predict how temperate macroalgal communities will respond to future heatwaves.

Long-term observations are crucial for identifying both abrupt and gradual changes in community composition in response to marine heatwaves^12^. The Santa Barbara Coastal Long Term Ecological Research (SBC LTER) program has measured species-specific biomass of macroalgae annually at nine rocky reef sites off the mainland coast of the Santa Barbara Channel since 2001 to detect such patterns and identify the processes driving them. Two recent marine heatwaves, the first and strongest in 2014–2015 (the warm blob) and the second in the summer of 2018, manifested as strong positive temperature anomalies along coastal California, including the Santa Barbara Channel^37–39^. During the 2014–2015 heatwave, which occurred across the entire northeast Pacific^40^, intense surface stratification limited upwelling efficiency, allowing anomalously high ocean temperatures to persist for two years, from the winter of 2014 to the winter of 2016^37^. Three years later, another marine heatwave occurred off southern California and Baja California, bringing anomalously warm waters to the coastal ocean in the summer and fall of 2018^38^.

We used long-term monitoring data from the SBC LTER to explore the initial impact of these two marine heatwaves on subtidal macroalgal community structure and the legacy left by them over the past decade. We evaluated whether the response of macroalgae to heatwave duration was consistent across taxonomic and functional groups and assessed whether warm-tolerant taxa and introduced species increased and persisted after the heatwaves. We show dramatic shifts in community composition during heatwave years, including rapid declines in understory kelps, particularly the dominant species *Pterygophora californica*, and increases in subtropical and introduced species. Macroalgal communities, however, were resilient, and understory kelps recovered quickly after heatwave conditions subsided. Contrary to predictions from laboratory studies^31^, the calcareous species *Corallina officinalis* increased during the heatwaves, and has remained more abundant since, highlighting the importance of in situ assessments of thermal tolerance under realistic heatwave conditions. Both acute and gradual increases in multiple species of GCA, including *Corallina officinalis, Amphiroa beauvoisii*, and *Calliarthron cheilosporioides*, suggest that this functional group may be particularly successful under warmer conditions with more frequent marine heatwaves.

## Results

### Identifying thermal anomalies and marine heatwaves

**Fig. 1:**
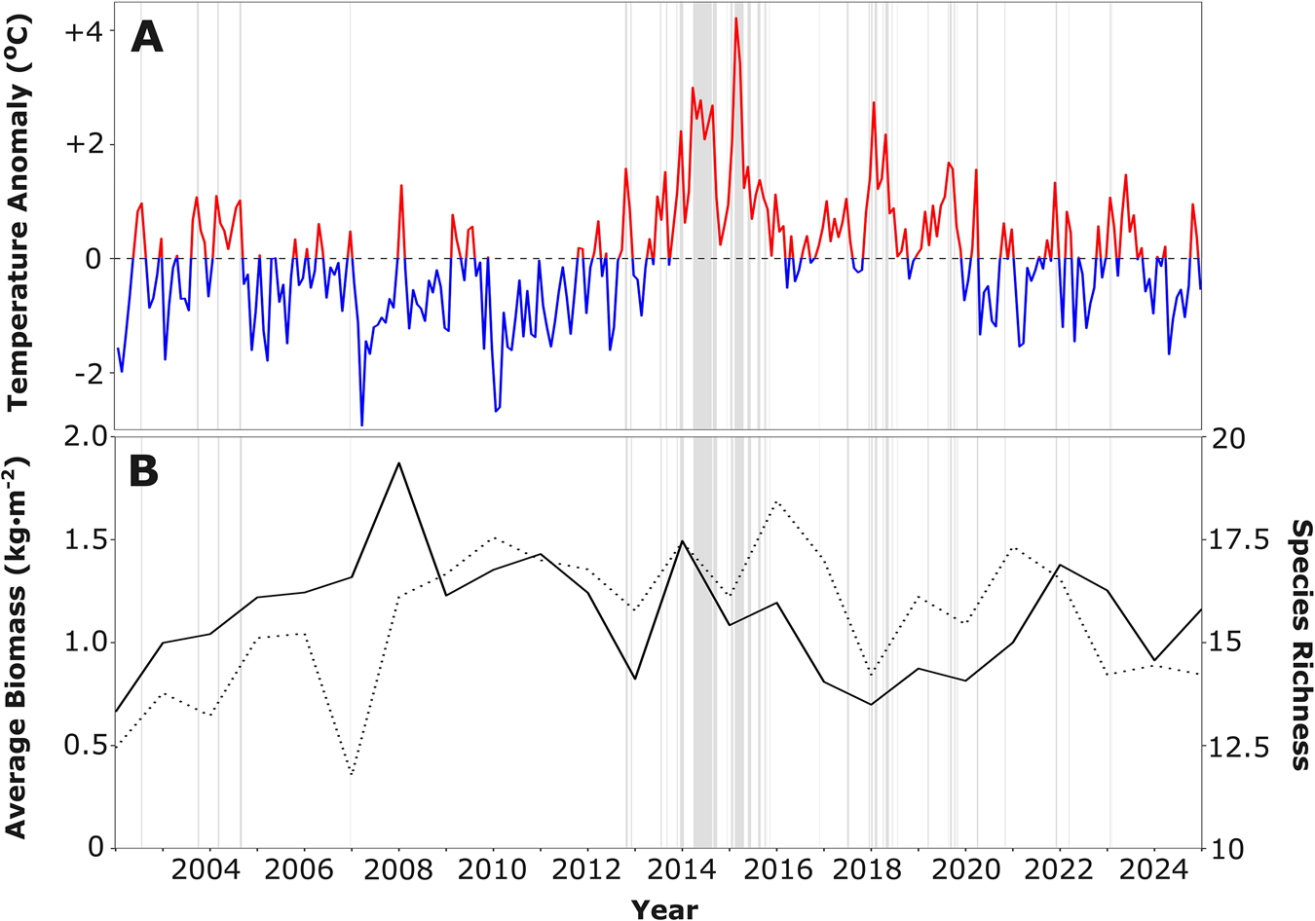
Thermal anomalies and average understory macroalgal biomass and richness during heatwave periods. **A** Average monthly bottom temperature anomaly across the nine sites. Positive anomalies are shown in red, while negative anomalies are shown in blue. **B** Average understory macroalgal biomass across sites is shown in the solid black line, while average species richness is shown in the dotted black line. Heatwave periods are shown in gray shading. Tick marks on the x-axis signify July (the month in which sampling occurred).

Both the 2014–2015 and 2018 heatwave periods sustained large positive bottom temperature anomalies, particularly during the summer and fall months (Fig. 1A). Anomalies were highest in fall of 2015 when the average monthly anomaly exceeded +4 °C (Fig. 1A). On average across sites, 49.6% (July 2014–June 2015), 41.6% (July 2015–June 2016), and 20.6% of days (July 2018–June 2019) were classified as heatwave days (Supplementary Fig. 1A). The longest continuous stretches of heatwave conditions were observed from October 2014 to February 2015 (146 days) and from August to October 2015 (71 days). All years since 2013 have had at least one heatwave event.

### Reef-wide shifts in community composition during marine heatwaves

Total understory macroalgal biomass and species richness during and after the 2014–2015 heatwave remained within or above the range observed before the heatwave (2002–2014) and increased from 2019 to 2022 following the 2018 heatwave (Fig. 1B). Heatwave duration was not a significant predictor of understory biomass or richness over time. Instead, understory biomass and richness were negatively associated with the biomass of the major overstory canopy species, *Macrocystis pyrifera* (Table 1, Models 1 and 2 and Supplementary Figs. 2 and 3). Biomass of *Macrocystis* varied considerably across sites and over time (Supplementary Fig. 4) but was also unresponsive to heatwave duration (Table 1, Model 3 and Supplementary Figs. 4 and 5).

**Table 1:** Model results for all generalized linear mixed effects models and linear mixed effects models

Model	Response	Predictors	DF	β	95% CI	*SE*	*z*	*p*
1	Total understory biomass	**Intercept**	179	33.188	[29.487, 36.888]	1.888	17.577	**<0.001**
		***Macrocystis*** **biomass**		−1.635	[−−3.061, −0.209]	0.728	−2.248	**0.025**
	Random Effects		Groups	Correlation	Observations			
		Year|Site	8	0.89	184			
2	Total understory richness	**Intercept**	178	16.004	[14.105, 17.904]	0.969	16.514	**<0.001**
		Proportion of Heatwave Days		2.782	[−1.964, 7.528]	2.422	1.149	0.251
		***Macrocystis*** **biomass**		−0.990	[−1.608, −0.372]	0.315	−3.141	**0.002**
	Random Effects		Groups	Correlation	Observations			
		Year|Site	8	0.90	184			
3	*Macrocystis* biomass	**Intercept**	179	−1.321	[−1.793, −0.868]	0.232	−5.694	**<0.001**
		Proportion of Heatwave Days		0.944	[−0.215, 2.101]	0.563	1.677	0.094 *
	Random Effects		Groups	Correlation	Observations			
		Year|Site	8	0.95	184			
4	Subtropical species biomass	**Intercept**	177	3.737	[3.043, 4.430]	0.354	10.560	**<0.001**
		**Proportion of Heatwave Days**		1.305	[0.341, 2.269]	0.492	2.653	**0.008**
		***Macrocystis*** **biomass**		−0.294	[−0.429, −0.160]	0.069	−4.279	**<0.001**
	Random Effects		Groups	Correlation	Observations			
		Year|Site	8	0.96	184			
5	Subtropical species biomass	**Intercept**	179	−135.098	[−170.253, −99.942]	17.937	−7.532	**<0.001**
		**Year**		0.069	[0.052, 0.086]	0.009	7.759	**<0.001**
	Random Effects		Groups	Observations				
		Site	8	184				
6	Transitional species biomass	Conditional Model	177					
		**Intercept**		−2.041	[−2.649, −1.434]	0.310	−6.584	**<0.001**
		Proportion of Heatwave Days		1.323	[−0.073, 2.719]	0.712	1.857	0.063 *
		*Macrocystis* biomass		0.171	[−0.046, 0.389]	0.111	1.542	0.123
		Dispersion Model						
		**Intercept**		2.514	[2.067, 2.961]	0.228	11.019	**<0.001**
		*Macrocystis* biomass		−0.191	[−0.490, 0.108]	0.153	−1.252	0.211
	Random Effects		Groups	Correlation	Observations			
		Year|Site	8	0.86	184			
7	Introduced species biomass	**Intercept**	179	−6.560	[−12.921, −0.200]	3.245	−2.022	**0.043**
		**Proportion of Heatwave Days**		11.109	[6.827, 15.390]	2.184	5.086	**<0.001**
	Random Effects		Groups	Observations				
		Site	8	184				
8	Temperate species biomass	**Intercept**	179	−1.240	[−1.603, −0.779]	0.198	−6.268	**<0.001**
		*Macrocystis* biomass		−0.120	[−0.265, 0.031]	0.072	−1.666	0.096 *
	Random Effects		Groups	Correlation	Observations			
		Year|Site	8	0.87	184			
**Model**	**Response**	**Predictors**	**DF**	***β***	**95% CI**	***SE***	***z***	***p***
9	Understory kelp biomass	**Intercept**	177	5.132	[4.320, 5.944]	0.414	12.388	**<0.001**
		**Proportion of Heatwave Days**		−2.562	[−4.118, −1.005]	0.794	−3.225	**0.001**
		*Macrocystis* biomass		0.153	[−0.022, 0.327]	0.089	1.718	0.086 *
	Random Effects		Groups	Correlation	Observations			
		Year|Site	8	0.87	184			
10	GCA biomass	**Intercept**	179	−188.496	[−223.861, −153.131]	18.044	−10.45	**<0.001**
		**Year**		0.095	[0.078, 0.113]	0.009	10.65	**<0.001**
	Random Effects		Groups	Observations				
		Site	8	184				
11	GCA biomass	**Intercept**	177	3.222	[2.322, 4.122]	0.459	7.014	**<0.001**
		Proportion of Heatwave Days		0.526	[−0.543, 1.594]	0.545	0.964	0.335
		***Macrocystis*** **biomass**		−0.180	[−0.318, −0.041]	0.071	−2.546	**0.011**
	Random Effects		Groups	Correlation	Observations			
		Year|Site	8	0.96	184			
12	Non-kelp brown biomass	**Intercept**	179	5.653	[5.245, 6.060]	0.208	27.205	**<0.001**
		**Proportion of Heatwave Days**		1.711	[0.686, 2.736]	0.523	3.271	**0.001**
	Random Effects		Groups	Observations				
		Site	8	184				
13	Non−coralline red biomass	**Intercept**	179	−1.629	[−2.082, −1.176]	0.231	−7.049	**<0.001**
		***Macrocystis*** **biomass**		−0.165	[−0.316, −0.013]	0.077	−2.125	**0.034**
	Random Effects		Groups	Correlation	Observations			
		Year|Site	8	0.89	184			
14	Green biomass	Intercept	177	0.482	[−0.342, 1.307]	0.421	1.146	0.252
		Proportion of Heatwave Days		1.847	[−0.179, 3.873]	1.034	1.787	0.074 *
		***Macrocystis*** **biomass**		−1.053	[−1.501, −0.605]	0.229	−4.608	**<0.001**
	Random Effects		Groups	Correlation	Observations			
		Year|Site	8	0.86	184			

Degrees of freedom reported are residual degrees of freedom. Significant effects are bolded.

*signifies effects with *p* values between 0.05 and 0.10.

**Fig. 2:**
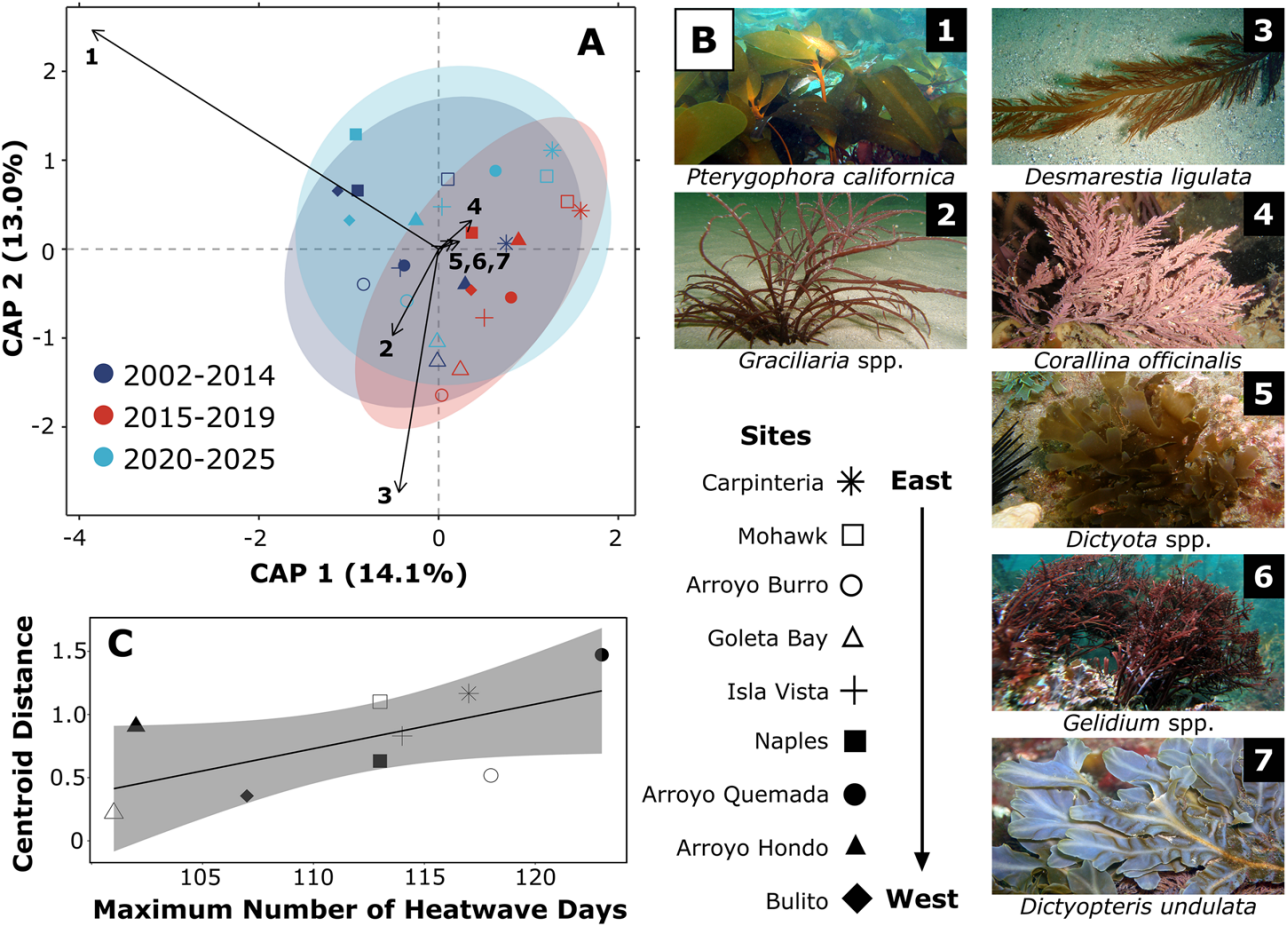
Shifts in macroalgal species composition during and following strong heatwave years. **A** Canonical Analysis of Principal Coordinates for community composition across the nine sites and three time periods (*R*^2^ = 0.488). The ordination was constrained by the interaction between site and time period (*F* = 5.916, *p* < 0.001), and points represent centroids for each site and time period interaction. The ellipses represent the spread of all points during each time period across all sites and years (Supplementary Fig. 6). Shapes represent sites. Numbered arrows correspond to the species that contributed the most to community dissimilarity over time with *r* > 0.20 which are shown in (**B**). **C** Correlation between the difference in average community composition at a site before (2002–2014) vs. after (2020–2025) the heatwave years (2015–2019) against the maximum number of days per year when bottom temperature exceeded the site-specific 90th percentile temperature threshold. Community differences were estimated using the distances in the average community centroids for each site from (**A**) for each time period. The solid line represents the linear fit and 95% confidence interval in gray shading (Supplementary Table 1).

Although total understory biomass remained high, there were significant differences in community composition before (2002–2014), during (2015–2019), and after (2020–2025) the strong heatwave years (PERMANOVA, *F* = 9.8326, *p* < 0.001; Fig. 2A). Biomass of the dominant understory kelp *Pterygophora californica* was negatively associated with heatwave conditions, while biomass of *Corallina officinalis, Dictyota* spp., *Dictyopteris undulata*, and *Gelidium* spp. were positively associated with heatwave and post-heatwave conditions, particularly at the warmest sites (Fig. 2A).

**Fig. 3:**
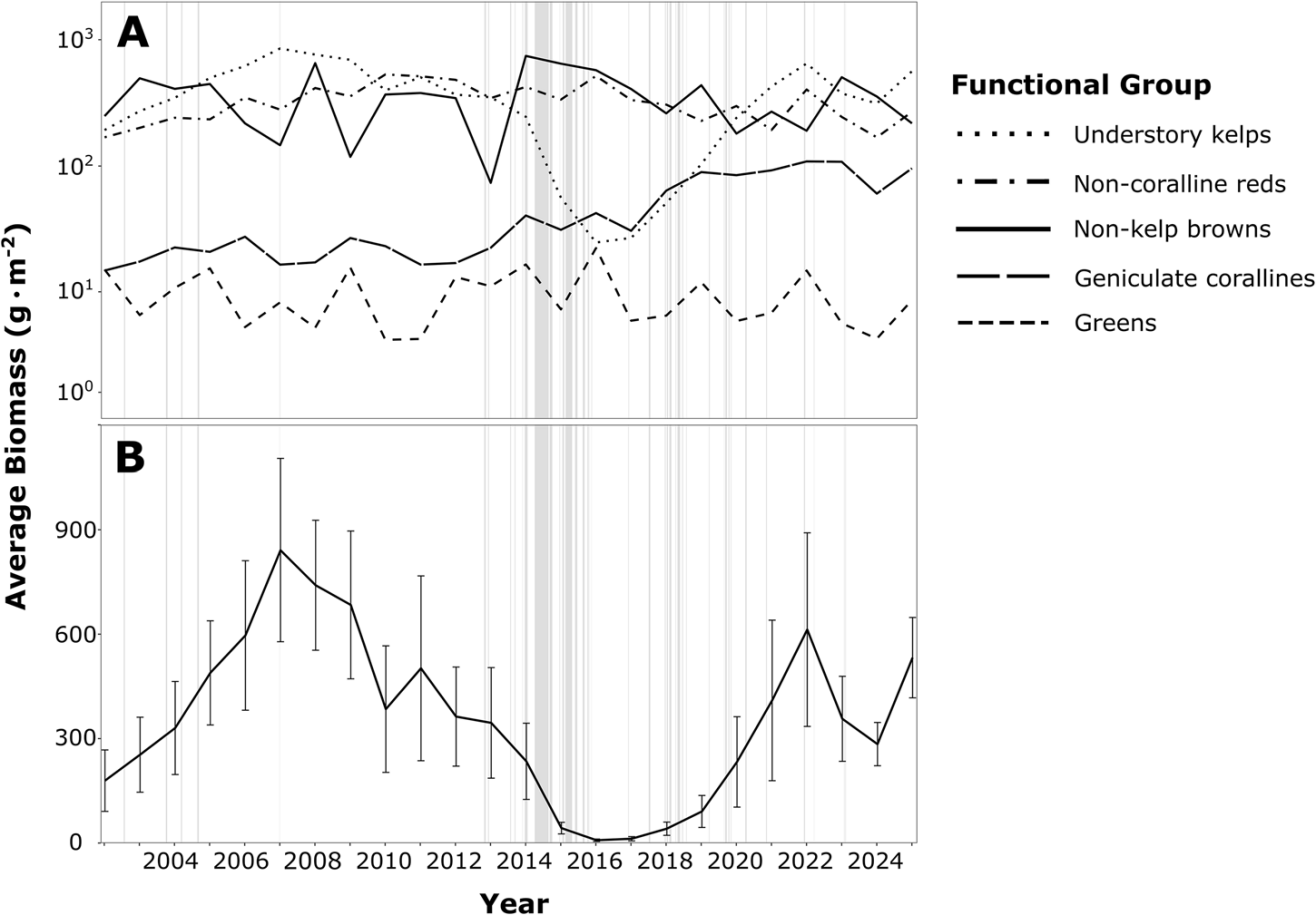
Average functional group responses to heatwaves over time. Mean annual biomass of understory macroalgae averaged across sites by (**A**) functional group and (**B**) *Pterygophora californica* only (understory kelp). Biomass is represented on a log scale for (**A**). Error bars represent standard error. Heatwave days are shown in gray shading. Tick marks on the x-axis signify July (the month in which sampling occurred).

*Pterygophora* biomass declined sharply to near zero at all sites during the 2014–2015 heatwave, reaching a 23-year minimum in 2016 (Fig. 3B and Supplementary Fig. 7; IndVal = 0.971, *p* < 0.001). Prior to heatwave conditions, biomass of this species was variable across sites and consistently high at the coolest, westernmost site, Bulito, until the 2014–2015 heatwave (Supplementary Fig. 7A). Its recovery began in 2018 at the coolest sites (e.g., Bulito and Naples) before progressing to the warmer sites beginning in 2020 (Supplementary Fig. 7). The loss of *Pterygophora* accounted for the largest shifts in community composition during heatwave years (Fig. 2A).

There was a positive correlation between the extent of warming and the degree to which community composition shifted over time as sites with more anomalously warm days in a given year exhibited larger shifts than those with fewer anomalously warm days (Fig. 2C and Supplementary Table 1). Site-specific shifts in species composition were also positively correlated with heatwave intensity from July 2018 to June 2019 (Supplementary Table 1).

### Community homogenization during and following marine heatwaves

**Fig. 4:**
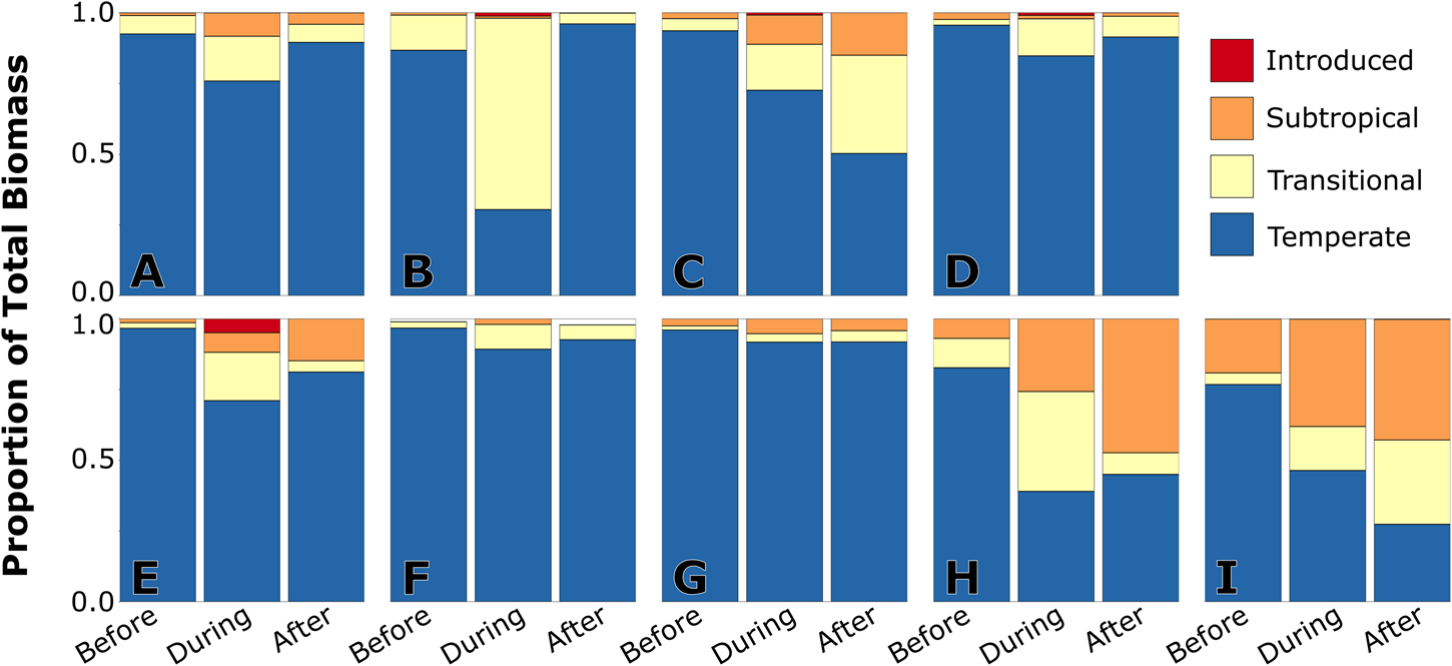
Proportion of total understory biomass within each thermal group before (2002–2014), during (2015–2019), and after (2020–2025) strong heatwave years. Panels represent proportions at each site across the thermal gradient from west to east at (**A**) Bulito, (**B**) Arroyo Hondo, (**C**) Arroyo Quemada, (**D**) Naples, (**E**) Isla Vista, (**F**) Goleta Bay, (**G**) Arroyo Burro, (**H**) Mohawk, and (**I**) Carpinteria reefs.

Prior to heatwave conditions, all sites differed significantly in composition across the thermal gradient (ANOSIM *R* = 0.497, *p* = 0.001, Fig. 2A), though the two easternmost sites exhibited the highest proportion of subtropical species (Fig. 4H, I). The proportional biomass of subtropical species increased across most sites during the heatwave years, but this increase was the highest at the easternmost sites, where their biomass increased from 7.0% to 25.7% at Mohawk and from 19.1% to 37.7% at Carpinteria (Fig. 4).

Sites remained significantly different from one another in composition during (ANOSIM, *R* = 0.580, *p* = 0.001) and following (ANOSIM, *R* = 0.663, *p* = 0.001, Figs. 2A and 4) the strong heatwave years, suggesting that community composition did not converge across the thermal gradient in response to more frequent heatwaves. However, composition became more similar at three sites: Carpinteria, Mohawk, and Arroyo Quemada (Fig. 2A). Carpinteria and Mohawk exhibited the highest proportion of subtropical species in total biomass during and after the heatwave periods (44.7% and 50.7%, respectively), while the proportion of transitional species increased during and after the heatwave years at Arroyo Quemada (16.2–41.9%) and Carpinteria (15.6% to 26.9%, Fig. 4). The contribution of temperate species returned to pre-heatwave levels at most sites except Arroyo Quemada, Mohawk, and Carpinteria, where temperate species only contributed 41.5%, 40.1%, and 28.3%, respectively, after the strong heatwave years (Fig. 4).

### Thermal group shifts during marine heatwaves

Heatwave duration was a significant positive predictor of subtropical species biomass over time (Table 1, Model 4, Supplementary Fig. 8). Transitional species biomass increased with increasing heatwave duration, though this trend was not significant (Table 1, Model 6, Supplementary Fig. 9). Both groups increased in biomass steadily during the strongest heatwave years (Supplementary Fig. 10), though increases of transitional species beginning in 2015 were driven largely by the dominant brown fucoid *Stephanocystis osmundacea* and the GCA species *Calliarthron cheilosporioides* (Supplementary Fig. 11). Subtropical species increased in biomass by 140.0% (2014–2016) and 64.5% (2018–2019) following the strong heatwave years in 2015 and 2018 (Supplementary Fig. 12). Biomass subsequently declined in 2017 and 2020 but increased significantly over time across the thermal gradient (Table 1, Model 5 and Supplementary Fig. 13), predominantly due to increases in the GCA species *Corallina officinalis* (Supplementary Fig. 12). Increases in two brown algae, *Dictyota* spp. and *Dictyopteris undulata*, and two red algae, *Sarcodiotheca furcata* and *Acrosorium ciliolatum*, were pronounced in 2016 and 2019 (Supplementary Fig. 12), though these increases were largely restricted to the warmest sites. *Dictyopteris undulata* (IndVal = 0.366, *p* = 0.020), *Acrosorium ciliolatum* (IndVal = 0.639, *p* < 0.001), *Sarcodiotheca furcata* (IndVal = 0.498, *p* = 0.002), and the GCA species *Amphiroa beauvoisii* (IndVal = 0.366, *p* = 0.002, Supplementary Fig. 12) were significantly more abundant during and after heatwave years compared to the period prior to heatwave years.

Heatwave duration was also a significant positive predictor of introduced species biomass (Table 1, Model 7 and Supplementary Fig. 14), which peaked in 2015 during the 2014–2015 heatwave (Supplementary Figs. 10 and 15). Biomass of the Asian fucoid *Sargassum muticum* increased during the heatwave years (Supplementary Fig. 15) and was significantly more abundant from 2015–2019 than before or after the strong heatwave years (IndVal = 0.457, *p* < 0.001). Its less abundant congener *Sargassum horneri* was only observed at the warmest site, Carpinteria, where its biomass was significantly higher after the strong heatwave years compared to before and during heatwave years (IndVal = 0.445, *p* < 0.001, Supplementary Fig. 15). There was no effect of heatwave duration on temperate species, which comprise the majority of surveyed species (Table 1, Model 8 and Supplementary Fig. 16).

### Functional group susceptibility to marine heatwaves

Biomass in three of the five functional groups shifted during strong heatwave years (Fig. 3A). Heatwave duration was a significant negative predictor of understory kelp biomass (Table 1, Model 9 and Supplementary Fig. 17), which is composed primarily of the dominant species *Pterygophora californica* (Supplementary Fig. 18). *Macrocystis* biomass was not a significant predictor of understory kelp biomass over time (Table 1, Model 9 and Supplementary Fig. 17), though it was a significant negative predictor of biomass of GCA (Table 1, Model 11 and Supplementary Fig. 19), non-coralline red (Table 1, Model 13 and Supplementary Fig. 20) and green algae (Table 1, Model 14 and Supplementary Fig. 21).

Biomass of understory kelps declined during the 2014–2015 heatwave, reaching a minimum in 2016 and remaining low until after the 2018 heatwave (Fig. 3B and Supplementary Fig. 18). The biomass of GCA, particularly *Corallina officinalis, Calliarthron cheilosporioides*, and *Amphiroa beauvoisii*, increased significantly over time, particularly during and after 2018, though this was not affected by heatwave duration (Table 1, Models 10, 11 and Fig. 3A, Supplementary Figs. 19, 22). Heatwave duration was a significant positive predictor of non-kelp brown algal biomass but was unrelated to the biomass of non-coralline red or green algae (Table 1, Models 12–14 and Supplementary Figs. 23, 20–21).

## Discussion

There were significant shifts in understory macroalgal community composition across the mainland coast of the Santa Barbara Channel during the severe and prolonged 2014–2015 heatwave and the subsequent 2018 heatwave. Though richness and biomass remained relatively unchanged during the most severe heatwave conditions, community composition shifted considerably across sites with notable changes in two functional groups: increases in geniculate coralline algae over time and declines in understory kelps. However, declines in understory kelps and increases in non-kelp brown algae were driven either by a few large dominant species, *Pterygophora californica* (kelp) and *Stephanocystis osmundacea* (non-kelp brown), or select subtropical species of non-kelp brown algae such as *Dictyota* and *Dictyopteris*. This suggests that responses to heatwaves were mostly driven by species-specific responses rather than by coherent functional groups. Nevertheless, increases in subtropical species, both during strong heatwave years and over time, suggest that species with higher thermal tolerance benefit from anomalously warm conditions.

The dominant canopy species, *Macrocystis*, was resistant to warming within the Santa Barbara Channel, potentially due to temperatures remaining below the species’ critical thermal threshold^36,39^. In contrast, strong declines were observed towards the southern limit of its range in the northeast Pacific, in other parts of southern California, in Baja California during the 2014–2015 heatwave^36,41^, and in Australia and New Zealand, due to warming and heatwaves^42–44^.

The most striking impact of the 2014–2015 heatwave was the loss of the dominant understory kelp, *Pterygophora californica*. During that period, we observed the crowns of this species turning black and disintegrating (Miller, pers. obs.). Species-rich systems can exhibit higher stability due to species’ asynchronous responses to environmental fluctuations; such species insurance^45^ compensated for the loss of *Pterygophora*, resulting in little net change in total understory biomass and richness during strong heatwave years^46^. In contrast to other locations, including Australia, New Zealand, and the eastern Pacific, where losses of canopy-forming kelps persisted following heatwave conditions^35,43,47,48^, *Pterygophora* populations recovered rapidly within five years following the heatwave period. Pre-heatwave *Pterygophora* populations were variable across sites, and this variability was not driven by overstory shading by *Macrocystis* (Supplementary Fig. 17 and Table 1, Model 9). Further investigation could help us understand the abiotic and biotic factors that affect recruitment and survival of this important algal species across spatial scales^49^.

*Pterygophora* recovery was strongest at Bulito and Naples, the sites with the largest and least variable pre-heatwave populations (Supplementary Fig. 7). The subsequent 2018 heatwave, although it may have slowed recovery, was not associated with net declines at these sites (Supplementary Fig. 7), perhaps due to differences in the intensity or duration of thermal stress or nutrient availability during the growth season between the two heatwaves. In contrast to the 2014–2015 heatwave, where positive temperature anomalies began during the spring months, limiting mixing and nutrient delivery to surface waters^37^, the 2018 heatwave began during summer months^38,50^ when wind-driven upwelling is generally less important for nutrient delivery in the Santa Barbara Channel^51^. Water column chlorophyll-*a* concentrations were lower during the springs of 2014 and 2015 compared to 2018^52^, suggesting that nutrients may not have been as limiting during the 2018 heatwave. Both the timing and duration of future heatwaves and their subsequent effects on nutrient availability will affect the capacity of *Pterygophora* populations to resist declines or recover from biomass losses, particularly as this species has demonstrated limited reproductive capacity at elevated temperatures^53,54^.

There were significant shifts in the composition of understory macroalgal communities during and after the heatwave years, particularly increases in the abundance of subtropical GCA. Local increases in *Corallina officinalis*, the dominant species of GCA at our sites, during strong heatwave years suggest high thermal tolerance in this species. However, laboratory experiments present conflicting evidence depending on the physiological metric measured and thermal treatments; growth rates of *Corallina officinalis* remained unaffected under conditions of up to 28 °C in one manipulation^55^, while *Corallina officinalis* exhibited decreased physiological performance above a 23 °C threshold following 7 weeks of thermal stress in another experiment^31^, suggesting drastically different thermal limits. Calcification rates across measured coralline species decline, on average, at thermal extremes +5 °C above ambient^32^. During the warmest month of the 2014–2015 heatwave, temperatures reached an average of 21.1 °C across the mainland of the Santa Barbara Channel, about +4.2 °C above the monthly average. Increases in photosynthetic rate under warmer conditions may have given *Corallina* a competitive advantage in recent years, though future heatwaves above the species’ thermal tolerance limits may hinder its success. The subtropical species *Amphiroa beauvoisii* also increased in abundance, particularly during the strongest heatwave years. Similar increases in this species and other subtropical species of GCA occurred in Baja California following the 1997-1998 El Niño^33^ and during the 2009-2010 El Niño in the southern Mexican Pacific, where *Amphiroa* was observed overgrowing live corals^56^. Thus far, populations of *Amphiroa* have remained restricted to the warmest site in the SBC, though increases in geniculate corallines suggest this functional group may be particularly successful during future heatwaves either due to high thermal tolerance, high phenotypic plasticity, or life history traits that support rapid colonization^32^.

Increases in introduced species were mostly restricted to strong heatwave years. Prior observations in La Jolla, CA, during the 1982-1983 El Niño revealed an exceptionally high recruitment of the annual fucoid *Sargassum muticum* after the loss of two Laminarian kelps, *Egregia* and *Eisenia*^57^, suggesting *S. muticum* was able to colonize during periods with high temperatures and with competitive release of less thermally tolerant understory kelps. Laboratory experiments have shown the success of *S. muticum* over native species during heatwave conditions^58^, while increases in other non-native *Sargassum* species have occurred and persisted following the 2014–2015 MHW in Baja California^21^ and following the 1997-1998 El Niño in Southern Japan^59^. In contrast, biomass of *Sargassum muticum* peaked in 2015 and subsequently declined, suggesting that this species may have opportunistically established during years with favorable conditions but died back concomitantly with the recovery of understory kelps or the return of cooler waters.

Shifts in understory species composition have potential implications for benthic habitat and productivity^60^. Understory kelps like *Pterygophora* are highly productive and can comprise the majority of reef macroalgal biomass when *Macrocystis* is absent^61,62^. Losses of kelps and compensatory increases in non-natives would alter the physical structure of macroalgal habitat^63^. Shifts to either less productive coralline algae, including *Corallina officinalis*^62^, or non-natives^64^, would change the quality and quantity of food for grazers and detritivores. Omnivorous fishes and mobile invertebrates, including two dominant urchin species, *Mesocentrotus franciscanus* and *Strongylocentrotus purpuratus*, consume GCA in the Santa Barbara Channel^65^, but the nutritional quality or consumption rates of corallines compared to other macroalgal taxa have not yet been explored.

Understory communities shifted across sites in the Santa Barbara Channel in response to heatwave conditions; however, high species richness and distinct community composition across the sites suggest there was not a simplification or homogenization of community composition. Site-level variation in composition has persisted, and differences in abiotic conditions, reef structure, or biotic interactions across sites likely caused dissimilarity at this scale^49,66,67^. The greatest changes in composition occurred at sites where thermal anomalies were most persistent and where bottom temperatures were the highest in 2018, suggesting that the duration of heatwaves, particularly during the warmest months, will affect the degree to which communities respond to future disturbances. Understory communities were relatively resilient during strong heatwave years, and rapid recovery of the dominant understory kelp, *Pterygophora*, drove average community composition back towards pre-heatwave conditions. However, increases in biomass of subtropical and GCA species over time suggest that macroalgal communities will continue to shift in response to more frequent and intense marine heatwaves.

## Methods

### Study sites

**Fig. 5:**
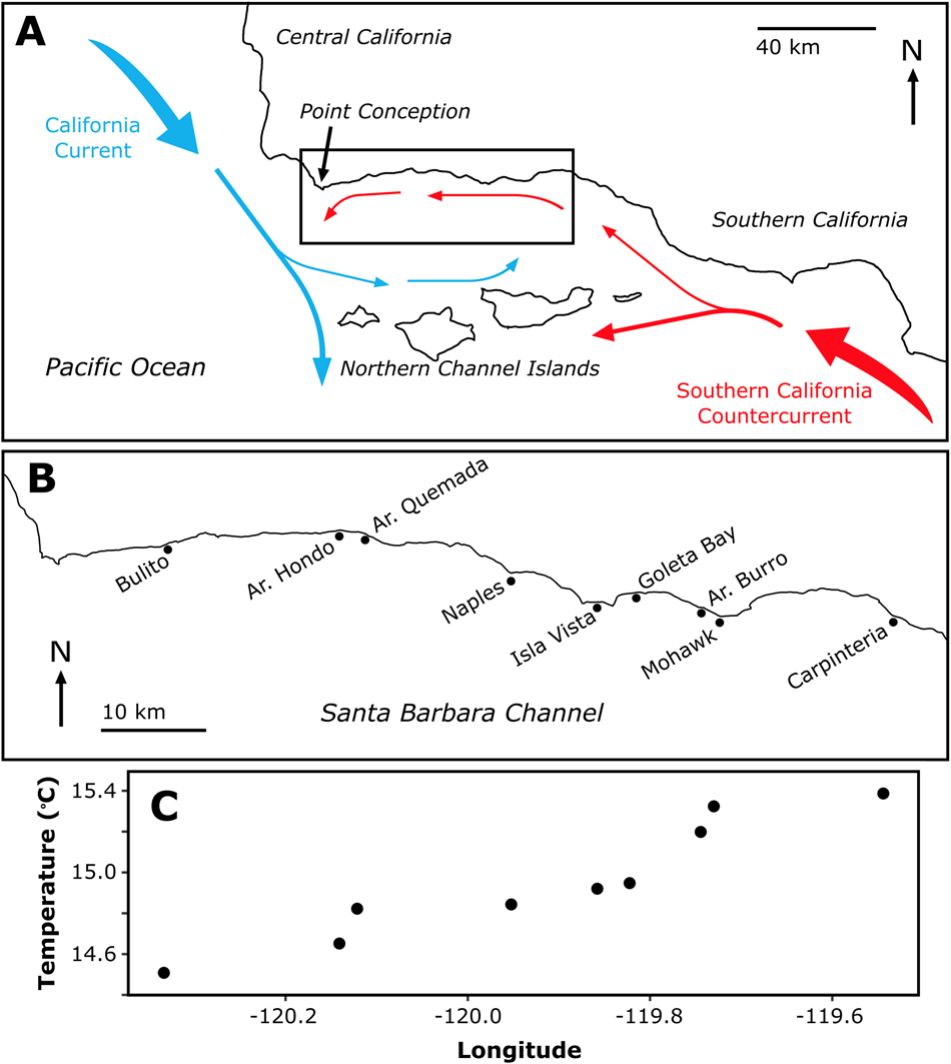
Oceanographic conditions across sampling sites within the Santa Barbara Channel. **A** Map of the convergence of two major currents flowing within the Santa Barbara Channel. Arrows represent the direction of flow of the northward-flowing Southern California Countercurrent (red arrows) and the southward-flowing California Current (blue arrows). The rectangular inset shows the locations of sites represented in (**B**). **B** Map of the 9 sites along the mainland coast of the Santa Barbara Channel surveyed by the SBC LTER. **C** Average annual bottom temperature across the longitudinal gradient from 2003 to 2025 in the Santa Barbara Channel. Points represent the average bottom temperature at each site shown in panel B. The longitudinal scale in (**C**) is consistent with (**B**).

The mainland coast of the Santa Barbara Channel is characterized by a longitudinal gradient in upwelling intensity and bottom temperature (Fig. 5A), which is largely due to its southward-facing coastline and the convergence of the southward flowing California Current and the northward-flowing Southern California Countercurrent^68^. This gradient contributes significantly to the variability in composition of macroalgal communities within the SBC LTER domain^49^. Though shifts in macroalgal community composition are more commonly observed at wider temperature gradients (e.g., 2–3 °C over spatial scales of hundreds to thousands of kilometers^69,70^), the Santa Barbara Channel offers a unique opportunity to observe the degree to which marine heatwaves alter the community structure of understory macroalgal communities that exist along a narrow thermal gradient of ~1 °C (Fig. 5C and Supplementary Fig. 1B). The convergence of currents forms a strong biogeographic barrier at Point Conception that bounds the flow of propagules from north of the point southward and eastward towards the mainland coast^71^. This boundary may contribute to homogenization of the macroalgal community from east to west following disturbance or in response to gradual warming as colder-affinity species are replaced by subtropical species moving westward.

The SBC LTER program has been monitoring community structure along permanent transects at nine mainland kelp forest reefs (see Supplementary Note 1 for site descriptions) annually in July since 2001^72^. Sites were located across ~ 78 km of the mainland coast of the Santa Barbara Channel (Bulito: 34.46 °N, 120.33 °W, Arroyo Hondo: 34.47 °N, 120.14 °W, Arroyo Quemada: 34.47 °N, 120.12 °W, Naples: 34.42 °N, 119.95 °W, Isla Vista: 34.40 °N, 119.86 °W, Goleta Bay: 34.41 °N, 119.82 °W, Arroyo Burro: 34.40 °N, 119.74 °W, Mohawk: 34.39 °N, 119.73 °W, Carpinteria: 34.39 °N, 119.54 °W; Fig. 5B). The number of permanent transects varied by site from 2 to 8 depending on the total area of the rocky reef^72^. Average transect depth varied between 2.5 m and 11 m below mean lower low water (MLLW) across sites, with a mean of 7 m.

### Data Collection

Bottom temperature was recorded every 15 min at each site since August 2002^73^ by fixed loggers (Stowaway Onset tidbits, Onset Computer, Bourne, Massachusetts, USA) at an average depth of 7 m MLLW.

Divers used a combination of swath, quadrat, and uniform point contact surveys to estimate abundance and biomass of 62 taxa of understory and canopy-forming macroalgae (Supplementary Table 2). Laminarian kelps, including *Macrocystis pyrifera, Laminaria farlowii, Pterygophora californica, Eisenia arborea* and the fucoid *Stephanocystis osmundacea* were counted and sized along fixed 40 m × 2 m transects by divers. The abundances of juvenile kelps and the non-native fucoids *Sargassum horneri* and *Sargassum muticum* were measured by counting and sizing individuals within 6 fixed 1 m^2^ quadrats uniformly spaced along the transect. The abundance of smaller species and those that are difficult to distinguish as individuals were measured using percent cover estimates from 80 uniformly spaced points along the transect.

For *Macrocystis pyrifera*, we converted measurements of frond density (no. per transect) to estimates of wet biomass density (g·m^−2^) using allometric relationships developed for each month of the year^74^. For all other species, we used taxon-specific allometric relationships to convert measurements of size-specific density or percent cover to wet biomass^72,75,76^. Wet biomass was then averaged across transects to obtain wet biomass by species at each site per year from 2003 to 2025.

### Identifying thermal anomalies and marine heatwaves

To estimate deviations in bottom temperature from the climatological mean and to identify periods when bottom temperatures were significantly higher than the climatological mean for prolonged periods, we calculated monthly bottom temperature anomalies for each site. Average monthly temperatures were calculated for each site from 2002 to 2025. For each site, we subtracted the average monthly temperature in a given year from the 23-year average temperature for that month. We then averaged the monthly anomalies across all nine sites to produce a monthly anomaly for the SBC region (Fig. 1A).

To identify marine heatwave events and estimate the severity of warming in a given year, the number of heatwave days per year was calculated for each site using the *detect()* function in the **heatwaveR** package^77^. The climatological mean for each site was calculated using bottom temperature data from August 2002 to July 2025^73^. Heatwave days were considered to be days when the average daily temperature exceeded the site-specific 90th percentile of the climatological mean for five or more consecutive days^78^. We estimated the degree of warming in the year preceding a benthic community survey, as the number of heatwave days in the calendar year beginning July 1^st^ of the previous year through June 30^th^ of the sampled year. The proportion of days in the year prior to sampling that were classified as heatwave days was calculated by dividing the number of heatwave days by the total number of days in the year (Supplementary Fig. 1A).

To evaluate whether heatwave severity or duration were related to the degree to which community composition shifted during the strong heatwave years from 2015–2019 (see *Reef-Wide Shifts in Community Composition During Marine Heatwaves* below*)*, we calculated the maximum annual number of heatwave days, the cumulative number of heatwave days from 2015–2019, and the cumulative intensity of heatwave conditions by site from 2015–2019. We also identified periods when bottom temperature exceeded the 90th percentile for a given site, excluding the climatological mean, using the *exceedance()* function. This estimate allowed us to assess whether the number of days that exceeded an absolute temperature threshold was an important indicator of thermal stress. For each site, we calculated the total number of days per year that bottom temperature exceeded the 90th percentile and identified the year during the heatwave period (2015–2019) with the most days that exceeded this threshold.

### Reef-wide shifts in community composition during marine heatwaves

We used linear mixed effects models and generalized linear mixed effects models in the **glmmTMB** package^79^ to evaluate whether the proportion of heatwave days in the year prior to sampling was a significant predictor of total understory macroalgal biomass and species richness. The proportion of heatwave days per year and the biomass of the overstory, canopy-forming giant kelp, *Macrocystis pyrifera*, were included in each model as fixed effects, as *Macrocystis* biomass has been shown to inhibit understory biomass and productivity at the study sites through its effect on light attenuation on the benthos^61,80,81^. A temporal autocorrelation term was included within site as a grouping variable to account for repeated measures.

We used *capscale*() in the **vegan** package^82^ to perform a Canonical Analysis of Principal Coordinates (CAP^83^), to assess whether average community composition per site in July varied over time. The ordination was constrained by the interaction between site and time period: before the heatwave years (2002–2014), during the strong heatwave years (2015–2019), or after the heatwave years (2020–2025).

We performed a Permutational Multivariate Analysis of Variance (PERMANOVA) using the function *adonis2*() in the **vegan** package^82^ to assess whether there were significant differences in species-based community composition as a function of time period. Prior to PERMANOVA, we evaluated whether groups exhibited homogeneity of group dispersions using the *betadisper*() function. Homogeneity of group dispersions between time periods did not differ significantly. Permutations were limited within each site as blocks.

We used the function *lm*() to evaluate whether linear correlations existed between heatwave severity from 2015–2019 and the shift in community composition by site from before the heatwave years (2002–2014) to after the heatwave years (2020–2025). We assessed multiple linear models with individual predictors estimating heatwave severity including the cumulative and maximum annual heatwave intensity (estimated from the *detect*() function in **heatwaveR)**, the cumulative and maximum annual duration of heatwave conditions from 2015–2019, and the cumulative and maximum annual duration of days that exceeded the absolute 90^th^ percentile temperature threshold for a given site. Average community composition shifts were estimated by calculating the distance in community centroids for each site from before and after the heatwave years from the CAP (Fig. 2A).

To identify which species were significantly more abundant during a given time period, we used the **indicspecies** package^84^ to perform an Indicator Species Analysis by time period across all sites.

### Community homogenization during and following marine heatwaves

We used the function anosim() in the **vegan** package to perform multiple Analyses of Similarity (ANOSIM) to evaluate whether community composition between sites became more similar during or following heatwave events. Community similarity was evaluated across sites during three time periods: before the strong heatwave years (2002–2014), during the strong heatwave years (2015–2019), and after the strong heatwave years (2020–2025). We also evaluated whether sites that shifted considerably in composition after the heatwave years (Fig. 2C) became more similar to the warmest sites, Mohawk and Carpinteria.

### Thermal group shifts during marine heatwaves

To assess whether warm-tolerant species have become more abundant over time or during years with many heatwave days, species were assigned to a thermal group corresponding to a historic range as defined by Abbott and Hollenberg^85^ (Supplementary Table 2). Most species surveyed by the SBC LTER have a broad geographical distribution that extends from either British Columbia or Alaska through Baja California, Mexico, and were classified as temperate. Species with ranges either extending beyond Baja California into central America or whose distribution was limited to the Santa Barbara Channel as the northernmost range limit were classified as subtropical. Non-native species to the Santa Barbara Channel were classified as introduced. Temperate species with a narrower thermal range bounded by southern Oregon and extending through Baja California were classified as transitional species. Biomass of species classified as northern-temperate, where the Santa Barbara Channel is at the southern-most edge of their range, was excluded from analyses as their biomass was negligible.

The changes in biomass of subtropical, introduced, transitional, and temperate species were evaluated in separate models against the proportion of heatwave days per year and the biomass of *Macrocystis pyrifera* using generalized linear mixed effects models. A temporal autocorrelation term was included within site to account for repeated measures for all models except for the model evaluating introduced species biomass. Inclusion of the temporal autocorrelation term prevented model convergence, and this term was excluded from the final model. To evaluate whether the change in biomass of subtropical species was significant over time, a separate model was fit with year as a predictor and site as a random effect.

For all linear and generalized linear mixed effects models presented, the **DHARMa** package^86^ was used to evaluate model fit and residuals. All final models were only accepted if the model Akaike Information Criterion (AIC) was significantly lower than that of the null model and there were no significant issues within the distribution of model residuals. Fixed effects were removed from the final model if they did not significantly improve model AIC. The best-fitting model for temperate species biomass was not significantly better than the null model, and the model with the lowest AIC value is presented. The distributions of all response variables and model parameters for generalized and linear mixed effects models are included in Supplementary Table 3.

### Functional group susceptibility to marine heatwaves

We used generalized linear mixed effects models to evaluate whether the proportion of heatwave days in the year prior to sampling was a significant predictor of functional group biomass. Species were assigned to one of the following functional groups: understory kelps, non-kelp brown algae, green algae, GCA, and non-coralline red algae based on taxonomic relatedness (Supplementary Table 2). Initial models included the proportion of heatwave days in the year prior to sampling and *Macrocystis pyrifera* biomass (standardized to a mean of 0 and a standard deviation of 1) as fixed effects. A temporal autocorrelation term was included within site as a grouping variable to account for repeated measures.

There was a general positive trend in GCA biomass over time (Fig. 3A), and a separate model with year as a fixed effect and site as a random effect was fit to evaluate whether the biomass of GCA changed significantly over time.

### Reporting summary

Further information on research design is available in the Nature Portfolio Reporting Summary linked to this article.

## Supplementary information

Transparent Peer Review file

Supplementary Material

Reporting Summary

## Data Availability

All data used in our analysis is publicly available from the Santa Barbara Long Term Ecological Research program: Reed, D. C. & Miller, R. J. SBC LTER: reef: bottom temperature: continuous water temperature, ongoing since 2000 ver 33. Environmental Data Initiative (2026). doi:10.6073/pasta/e565d409b63f20c768133c653ccdb2d7. Reed, D. C. & Miller, R. J. SBC LTER: Reef: Annual time series of biomass for kelp forest species, ongoing since 2000 ver 18. Environmental Data Initiative (2025). doi:10.6073/pasta/cb45bf15430ba570828444b13dd8521f

## Code availability

All code is publicly available: Michaud, K. M. Macroalgal community analysis Michaud et al. 2026 ver 2. Zenodo. 10.5281/zenodo.19617116 (2025).

## References

[1] Hoek, C. V. den. The distribution of benthic marine algae in relation to the temperature regulation of their life histories. *Biol. J. Linn. Soc.* **18**, 81–144 (1982).

[2] Harley, C. D. G., Smith, K. F. & Moore, V. L. Environmental variability and biogeography: the relationship between bathymetric distribution and geographical range size in marine algae and gastropods. *Glob. Ecol. Biogeogr.* **12**, 499–506 (2003).

[3] Tomanek, L. & Helmuth, B. Physiological ecology of rocky intertidal organisms: a synergy of concepts. *Integr. Comp. Biol.* **42**, 771–775 (2002). 21708774 10.1093/icb/42.4.771

[4] Tittensor, D. P. et al. Global patterns and predictors of marine biodiversity across taxa. *Nature* **466**, 1098–1101 (2010). 20668450 10.1038/nature09329

[5] Adey, W. H. & Steneck, R. S. Thermogeography over time creates biogeographic regions: a temperature/space/time-integrated model and an abundance-weighted test for benthic marine algae. *J. Phycol.* **37**, 677–698 (2001).

[6] Cheng, L. et al. Past and future ocean warming. *Nat. Rev. Earth Environ.* **3**, 776–794 (2022).

[7] Cheng, L. et al. Improved estimates of ocean heat content from 1960 to 2015. *Sci. Adv.* **3**, e1601545 (2017). 28345033 10.1126/sciadv.1601545 PMC5345929

[8] Poloczanska, E. S. et al. Global imprint of climate change on marine life. *Nat. Clim. Change* **3**, 919–925 (2013).

[9] Burrows, M. T. et al. Ocean community warming responses explained by thermal affinities and temperature gradients. *Nat. Clim. Chang.* **9**, 959–963 (2019).

[10] Pinsky, M. L., Selden, R. L. & Kitchel, Z. J. Climate-driven shifts in marine species ranges: scaling from organisms to communities. *Annu. Rev. Mar. Sci.* **12**, 1–27 (2019). 10.1146/annurev-marine-010419-010916 31505130

[11] Smith, K. E. et al. Biological impacts of marine heatwaves. *Annu. Rev. Mar. Sci.* **15**, 119–145 (2022). 10.1146/annurev-marine-032122-121437 35977411

[12] Reed, D. C. et al. Responses of coastal ecosystems to climate change: insights from long-term ecological research. *Bioscience* **72**, 871–888 (2022).

[13] Holbrook, N. J. et al. A global assessment of marine heatwaves and their drivers. *Nat. Commun.* **10**, 2624 (2019). 31201309 10.1038/s41467-019-10206-z PMC6570771

[14] Oliver, E. C. J. et al. Longer and more frequent marine heatwaves over the past century. *Nat. Commun.* **9**, 1324 (2018). 29636482 10.1038/s41467-018-03732-9 PMC5893591

[15] Frölicher, T. L., Fischer, E. M. & Gruber, N. Marine heatwaves under global warming. *Nature* **560**, 360–364 (2018). 30111788 10.1038/s41586-018-0383-9

[16] Smith, K. E. et al. Socioeconomic impacts of marine heatwaves: Global issues and opportunities. *Science* **374**, eabj3593 (2021). 34672757 10.1126/science.abj3593

[17] Smale, D. A. et al. Marine heatwaves threaten global biodiversity and the provision of ecosystem services. *Nat. Clim. Change* **9**, 306–312 (2019).

[18] Sunday, J. M., Bates, A. E. & Dulvy, N. K. Thermal tolerance and the global redistribution of animals. *Nat. Clim. Change* **2**, 686–690 (2012).

[19] Cheung, W. W. L. et al. Projecting global marine biodiversity impacts under climate change scenarios. *Fish Fish* **10**, 235–251 (2009).

[20] Morley, J. W. et al. Projecting shifts in thermal habitat for 686 species on the North American continental shelf. *PLoS ONE* **13**, e0196127 (2018). 29768423 10.1371/journal.pone.0196127 PMC5955691

[21] Félix-Loaiza, A. C. et al. Marine heatwaves facilitate invasive algae takeover as foundational kelp. *Bot. Mar.* **65**, 315–319 (2022).

[22] Montie, S. & Thomsen, M. S. Long-term community shifts driven by local extinction of an iconic foundation species following an extreme marine heatwave. *Ecol. Evol.* **13**, e10235 (2023). 37384244 10.1002/ece3.10235 PMC10293786

[23] Free, C. M. et al. Impact of the 2014–2016 marine heatwave on US and Canada West Coast fisheries: Surprises and lessons from key case studies. *Fish Fish* **24**, 652–674 (2023).

[24] Villaseñor-Derbez, J. C., Arafeh-Dalmau, N. & Micheli, F. Past and future impacts of marine heatwaves on small-scale fisheries in Baja California, Mexico. *Commun. Earth Environ.* **5**, 623 (2024).

[25] Richardson, L. E. et al. Mass coral bleaching causes biotic homogenization of reef fish assemblages. *Glob. Chang. Biol.* **24**, 3117–3129 (2018). 29633512 10.1111/gcb.14119

[26] Harley, C. D. G. Climate change, keystone predation, and biodiversity loss. *Science* **334**, 1124–1127 (2011). 22116885 10.1126/science.1210199

[27] Teagle, H. et al. The role of kelp species as biogenic habitat formers in coastal marine ecosystems. *J. Exp. Mar. Biol. Ecol.* **492**, 81–98 (2017).

[28] Graham, M. H. Effects of local deforestation on the diversity and structure of Southern California giant kelp forest food webs. *Ecosystems* **7**, 341–357 (2004).

[29] Ronnback, P. et al. Ecosystem goods and services from Swedish coastal habitats: identification, valuation, and implications of ecosystem shifts. *AMBIO J. Hum. Environ.* **36**, 534–544 (2007). 10.1579/0044-7447(2007)36[534:egasfs]2.0.co;2 18074889

[30] Straub, S. C. et al. Resistance, extinction, and everything in between – the diverse responses of seaweeds to marine heatwaves. *Front. Mar. Sci.* **6**, 763 (2019).

[31] Rendina, F. et al. Physiological response of the coralline alga *Corallina officinalis* L. to both predicted long-term increases in temperature and short-term heatwave events. *Mar. Environ. Res.* **150**, 104764 (2019). 31376632 10.1016/j.marenvres.2019.104764

[32] Cornwall, C. E., Diaz-Pulido, G. & Comeau, S. Impacts of ocean warming on coralline algal calcification: meta-analysis, knowledge gaps, and key recommendations for future research. *Front. Mar. Sci.* **6**, 186 (2019).

[33] Carballo, J. L., Olabarria, C. & Osuna, T. G. Analysis of four macroalgal assemblages along the Pacific Mexican Coast during and after the 1997–98 El Niño. *Ecosystems* **5**, 0749–0760 (2002).

[34] Smale, D. A. & Wernberg, T. Extreme climatic event drives range contraction of a habitat-forming species. *Proc. R. Soc. B: Biol. Sci.* **280**, 20122829 (2013). 10.1098/rspb.2012.2829 PMC3574333 23325774

[35] Wernberg, T. et al. Climate-driven regime shift of a temperate marine ecosystem. *Science* **353**, 169–172 (2016). 27387951 10.1126/science.aad8745

[36] Cavanaugh, K. C. et al. Spatial variability in the resistance and resilience of giant kelp in Southern and Baja California to a multiyear heatwave. *Front. Mar. Sci.* **6**, 413 (2019).

[37] Zaba, K. D. & Rudnick, D. L. The 2014–2015 warming anomaly in the Southern California Current System observed by underwater gliders. *Geophys. Res. Lett.* **43**, 1241–1248 (2016).

[38] Wei, X. et al. Large-scale conditions for the record-setting Southern California marine heatwave of August 2018. *Geophys. Res. Lett*. **48**, (2021).

[39] Reed, D. et al. Extreme warming challenges sentinel status of kelp forests as indicators of climate change. *Nat. Commun.* **7**, 13757 (2016). 27958273 10.1038/ncomms13757 PMC5159872

[40] Lorenzo, E. D. & Mantua, N. Multi-year persistence of the 2014/15 North Pacific marine heatwave. *Nat. Clim. Change* **6**, 1042–1047 (2016).

[41] Arafeh-Dalmau, N. et al. Extreme marine heatwaves alter kelp forest community near its equatorward distribution limit. *Front. Mar. Sci.* **6**, 499 (2019).

[42] Layton, C. et al. Kelp forest restoration in Australia. *Front. Mar. Sci.* **7**, 74 (2020).

[43] Tait, L. W. et al. Loss of giant kelp, *Macrocystis pyrifera*, driven by marine heatwaves and exacerbated by poor water clarity in New Zealand. *Front. Mar. Sci.* **8**, 721087 (2021).

[44] Johnson, C. R. et al. Climate change cascades: shifts in oceanography, species’ ranges and subtidal marine community dynamics in eastern Tasmania. *J. Exp. Mar. Biol. Ecol.* **400**, 17–32 (2011).

[45] Yachi, S. & Loreau, M. Biodiversity and ecosystem productivity in a fluctuating environment: the insurance hypothesis. *Proc. Natl. Acad. Sci.* **96**, 1463–1468 (1999). 9990046 10.1073/pnas.96.4.1463 PMC15485

[46] Lamy, T. et al. Species insurance trumps spatial insurance in stabilizing biomass of a marine macroalgal metacommunity. *Ecology* **100**, e02719 (2019). 31081945 10.1002/ecy.2719

[47] Thomsen, M. S. et al. Local extinction of bull kelp (*Durvillaea* spp.) due to a marine heatwave. *Front. Mar. Sci.* **6**, 84 (2019).

[48] Rogers-Bennett, L. & Catton, C. A. Marine heat wave and multiple stressors tip bull kelp forest to sea urchin barrens. *Sci. Rep.* **9**, 15050 (2019). 31636286 10.1038/s41598-019-51114-y PMC6803666

[49] Lamy, T. et al. Scale-specific drivers of kelp forest communities. *Oecologia* **186**, 217–233 (2018). 29101467 10.1007/s00442-017-3994-1

[50] Amaya, D. J. et al. Physical drivers of the summer 2019 North Pacific marine heatwave. *Nat. Commun.* **11**, 1903 (2020). 32313028 10.1038/s41467-020-15820-w PMC7171163

[51] Winant, C. D., Dever, E. P. & Hendershott, M. C. Characteristic patterns of shelf circulation at the boundary between central and southern California. *J. Geophys. Res. Oceans* **108**, 3021 (2003).

[52] Michaud, K. M., Reed, D. C. & Miller, R. J. The Blob marine heatwave transforms California kelp forest ecosystems. *Commun. Biol.* **5**, 1143 (2022). 36307673 10.1038/s42003-022-04107-z PMC9614761

[53] Matson, P. G. & Edwards, M. S. Effects of ocean temperature on the southern range limits of two understory kelps, *Pterygophora californica* and *Eisenia arborea*, at multiple life-stages. *Mar. Biol.* **151**, 1941–1949 (2007).

[54] Muth, A. F. et al. Recruitment tolerance to increased temperature present across multiple kelp clades. *Ecology* **100**, e02594 (2019). 30615200 10.1002/ecy.2594

[55] Kim, J.-H. et al. Elevated temperature and changed carbonate chemistry: effects on calcification, photosynthesis, and growth of *Corallina officinalis* (Corallinales, Rhodophyta). *Phycologia* **57**, 280–286 (2017).

[56] Lopez-Perez, A. et al. Reef community changes associated with the 2009-2010 El Niño in the Southern Mexican Pacific. *Pac. Sci.* **70**, 175–190 (2016).

[57] Gunnill, F. C. Population fluctuations of seven macroalgae in Southern California during 1981–1983 including effects of severe storms and an El Niño. *J. Exp. Mar. Biol. Ecol.* **85**, 149–164 (1985).

[58] Atkinson, J. et al. Summer and winter marine heatwaves favor an invasive over native seaweeds. *J. Phycol.* **56**, 1591–1600 (2020). 32679619 10.1111/jpy.13051

[59] Tanaka, K. et al. Warming off southwestern Japan linked to distributional shifts of subtidal canopy-forming seaweeds. *Ecol. Evol.* **2**, 2854–2865 (2012). 23170219 10.1002/ece3.391 PMC3501636

[60] Foster, M. C., Byrnes, J. E. K. & Reed, D. C. Effects of five southern California macroalgal diets on consumption, growth, and gonad weight, in the purple sea urchin *Strongylocentrotus purpuratus*. *PeerJ* **3**, e719 (2015). 25653904 10.7717/peerj.719 PMC4304870

[61] Castorani, M. C. N., Reed, D. C. & Miller, R. J. Loss of foundation species: disturbance frequency outweighs severity in structuring kelp forest communities. *Ecology* **99**, 2442–2454 (2018). 30376154 10.1002/ecy.2485

[62] Miller, R. J., Reed, D. C. & Brzezinski, M. A. Partitioning of primary production among giant kelp (*Macrocystis pyrifera*), understory macroalgae, and phytoplankton on a temperate reef. *Limnol. Oceanogr.* **56**, 119–132 (2011).

[63] Krumhansl, K. & Scheibling, R. Detrital subsidy from subtidal kelp beds is altered by the invasive green alga *Codium fragile* ssp. fragile. *Mar. Ecol. Prog. Ser.* **456**, 73–85 (2012).

[64] Tait, L. W. et al. Assemblage and understory carbon production of native and invasive canopy-forming macroalgae. *J. Exp. Mar. Biol. Ecol.* **469**, 10–17 (2015).

[65] Byrnes, J. E. et al. Climate-driven increases in storm frequency simplify kelp forest food webs. *Glob. Chang. Biol.* **17**, 2513–2524 (2011).

[66] Bekkby, T. et al. Red sea urchins (*Echinus esculentus*) and water flow influence epiphytic macroalgae density. *Mar. Biol. Res.* **11**, 375–384 (2015).

[67] Toohey, B. D. The relationship between physical variables on topographically simple and complex reefs and algal assemblage structure beneath an *Ecklonia radiata* canopy. *Estuar. Coast. Shelf Sci.* **71**, 232–240 (2007).

[68] Simons, R. D. & Catlett, D. Regulation of a surface chlorophyll hotspot by wind-driven upwelling and eddy circulation in the Santa Barbara Channel, Southern California. *Prog. Oceanogr.* **217**, 103096 (2023).

[69] Smale, D. A. et al. Patterns and drivers of understory macroalgal assemblage structure within subtidal kelp forests. *Biodivers. Conserv.* **29**, 4173–4192 (2020).

[70] Wernberg, T. et al. Biogenic habitat structure of seaweeds change along a latitudinal gradient in ocean temperature. *J. Exp. Mar. Biol. Ecol.* **400**, 264–271 (2011).

[71] Horn, M. H. & Allen, L. G. A distributional analysis of California coastal marine fishes. *J. Biogeogr.* **5**, 23 (1978).

[72] Reed, D. C. & Miller, R. J. SBC LTER: Reef: Annual time series of biomass for kelp forest species, ongoing since 2000 ver 18. *Environ. Data Initiat.*10.6073/pasta/cb45bf15430ba570828444b13dd8521f (2025).

[73] Reed, D. C. & Miller, R. J. SBC LTER: reef: bottom temperature: continuous water temperature, ongoing since 2000 ver 33. *Environ. Data Initiat.*10.6073/pasta/e565d409b63f20c768133c653ccdb2d7.(2026).

[74] Rassweiler, A. et al. Improved estimates of net primary production, growth, and standing crop of *Macrocystis pyrifera* in Southern California. *Ecology* **99**, 2132–2132 (2018). 29956835 10.1002/ecy.2440

[75] Reed, D. C. et al. Estimating biomass of benthic kelp forest invertebrates from body size and percent cover data. *Mar. Biol.* **163**, 101 (2016).

[76] Nelson, C. J. et al. SBC LTER: reef: coefficients for estimating biomass from body size or percent cover for kelp forest species ver 3. *Environ. Data Initiat.*10.6073/pasta/0fe9233dabe35df5d61fb3b07f8fb51e (2021).

[77] Schlegel, R. W. & Smit, A. J. heatwaveR: a central algorithm for the detection of heatwaves and cold-spells. *J. Open Source Softw.* **3**, 821 (2018).

[78] Hobday, A. J. et al. A hierarchical approach to defining marine heatwaves. *Prog. Oceanogr.* **141**, 227–238 (2016).

[79] Brooks, M. E. et al. glmmTMB balances speed and flexibility among packages for zero-inflated generalized linear mixed modeling. * R. J.* **9**, 378–400 (2017).

[80] Detmer, A. R. et al. Variation in disturbance to a foundation species structures the dynamics of a benthic reef community. *Ecology* **102**, e03304 (2021). 33565608 10.1002/ecy.3304

[81] Castorani, M. C. N. et al. Disturbance structures canopy and understory productivity along an environmental gradient. *Ecol. Lett.* **24**, 2192–2206 (2021). 34339096 10.1111/ele.13849 PMC8518717

[82] Oksanen, J. et al. *Vegan*. *Community Ecology Package* R package version 2.7-2. 10.32614/CRAN.package.vegan (2025).

[83] Anderson, M. J. & Willis, T. J. Canonical analysis of principal coordinates: a useful method of constrained ordination for ecology. *Ecology* **84**, 511–525 (2003).

[84] De Cáceres, M. & Legendre, P. Associations between species and groups of sites: indices and statistical inference. *Ecol.* **90**, 3566–3574 (2009). 10.1890/08-1823.1 20120823

[85] Abbott, I. A. & Hollenberg, G. J. *Marine Algae of California* (Stanford University Press, 1992).

[86] Hartig, F. *DHARMa: Residual Diagnostics for Hierarchical (Multi-Level / Mixed) Regression Models* R package version 0.4.7. 10.32614/CRAN.package.DHARMa (2024).

