# Supplementary material for: Macroalgal community transformation during successive marine heatwaves in southern California kelp forests
Source: Commun Earth Environ. 2026 May 16;7(1):632. doi: 10.1038/s43247-026-03599-5 (PMC13437228; doi:10.1038/s43247-026-03599-5)
Supplement: Supplementary file 1 [file 43247_2026_3599_MOESM1_ESM.pdf]

nature portfolio

Peer Review File

Macroalgal community transformation during successive marine heatwaves in southern California kelp forests

Corresponding Author: Ms Kristen Michaud

This file contains all editorial decision letters in order by version, followed by all author rebuttals in order by version.

Attachments originally included by the reviewers as part of their assessment can be found at the end of this file.

Version 0:

Decision Letter:

** Please ensure you delete the link to your author home page in this e-mail if you wish to forward it to your coauthors **

Dear Ms Michaud,

Your manuscript titled "Macroalgal community transformation during successive marine heatwaves in southern California kelp forests" has now been seen by 3 reviewers, and we include their comments at the end of this message. They find your work of interest, but some important points are raised. We are interested in the possibility of publishing your study in Communications Earth & Environment, but would like to consider your responses to these concerns and assess a revised manuscript before we make a final decision on publication.

We therefore invite you to revise and resubmit your manuscript, along with a point-by-point response that takes into account the points raised. Please highlight all changes in the manuscript text file.

Please submit your point-by-point responses as a separate file, distinct from your cover letter where you can add responses to the Editors' comments that you do not want to be made available to the reviewers. Word files are preferred. We recommend that any figures, tables or graphs that are included in the response to reviewers are also included in the main article or Supplementary Information.

We are committed to providing a fair and constructive peer-review process. Please don't hesitate to contact us if you wish to discuss the revision in more detail.

Please use the following link to submit your revised manuscript, point-by-point response to the referees' comments (which should be in a separate document to any cover letter), a tracked-changes version of the manuscript (as a PDF file) and the completed checklist:
Link Redacted
** This url links to your confidential home page and associated information about manuscripts you may have submitted or be reviewing for us. If you wish to forward this email to co-authors, please delete the link to your homepage first **

We hope to receive your revised paper within six weeks; please let us know if you aren't able to submit it within this time so that we can discuss how best to proceed. If we don't hear from you, and the revision process takes significantly longer, we may close your file. In this event, we will still be happy to reconsider your paper at a later date, as long as nothing similar has been accepted for publication at Communications Earth & Environment or published elsewhere in the meantime.

Please do not hesitate to contact us if you have any questions or would like to discuss these revisions further. We look forward to seeing the revised manuscript and thank you for the opportunity to review your work.

Best regards,

Alice Drinkwater, PhD
Associate Editor
Communications Earth & Environment

Consulting Editor
Communications Sustainability

EDITORIAL POLICIES AND FORMATTING

We ask that you ensure your manuscript complies with our editorial policies. Please ensure that the following formatting requirements are met, and any checklist relevant to your research is completed and uploaded as a Related Manuscript file type with the revised article.

For Manuscripts that fall into the following fields:
• Behavioural and social science
• Ecological, evolutionary & environmental sciences
• Life sciences
An updated and completed version of our Reporting Summary must be uploaded with the revised manuscript
You can download the form here:
https://www.nature.com/documents/nr-reporting-summary.pdf

Furthermore, please align your manuscript with our format requirements, which are summarized on the following checklist:
Communications Earth & Environment formatting checklist

and also in our style and formatting guide Communications Earth & Environment formatting guide .

*** DATA: Communications Earth & Environment endorses the principles of the Enabling FAIR data project (http://www.copdess.org/enabling-fair-data-project/). We ask authors to make the data that support their conclusions available in permanent, publically accessible data repositories. (Please contact the editor if you are unable to make your data available).

All Communications Earth & Environment manuscripts must include a section titled "Data Availability" at the end of the Methods section or main text (if no Methods). More information on this policy, is available at http://www.nature.com/authors/policies/data/data-availability-statements-data-citations.pdf.

In particular, the Data availability statement should include:
- Unique identifiers (such as DOIs and hyperlinks for datasets in public repositories)
- Accession codes where appropriate
- If applicable, a statement regarding data available with restrictions
- If a dataset has a Digital Object Identifier (DOI) as its unique identifier, we strongly encourage including this in the Reference list and citing the dataset in the Data Availability Statement.

DATA SOURCES: All new data associated with the paper should be placed in a persistent repository where they can be freely and enduringly accessed. We recommend submitting the data to discipline-specific, community-recognized repositories, where possible and a list of recommended repositories is provided at http://www.nature.com/sdata/policies/repositories.

If a community resource is unavailable, data can be submitted to generalist repositories such as figshare or Dryad Digital Repository. Please provide a unique identifier for the data (for example a DOI or a permanent URL) in the data availability statement, if possible. If the repository does not provide identifiers, we encourage authors to supply the search terms that will return the data. For data that have been obtained from publically available sources, please provide a URL and the specific data product name in the data availability statement. Data with a DOI should be further cited in the methods reference section.

Please refer to our data policies at http://www.nature.com/authors/policies/availability.html.

REVIEWER COMMENTS:

Reviewer #1 (Remarks to the Author):

This manuscript examines the impacts of two successive marine heatwaves on macroalgal understory community structure. This manuscript covers a highly relevant topic in the environmental sciences, as it focuses on the recovery of the understory macroalgal communities after repeated MHWs, as well as the shifts in subtropical, transitional and introduced species. Although the impacts of MHWs, particularly the “warm blob”, have been frequently studied, as well as the time series being used multiple times, this manuscript is novel in the way that the data is analysed and the conclusions presented. The paper is well written and clear throughout, and the statements are supported by the statistical analyses performed.

I would, however, like to request additional contextual information for the different sites, particularly regarding overstory macroalgal biomass and how this may have influenced understory biomass. I acknowledge that this study focuses on the understory community structure, but more information on Macrocystis pyrifera would be valuable, especially since you found that the understory biomass and richness was negatively associated with M pyrifera biomass (L101-102), for which Fig. S3 and S4 support this. Including an additional Supporting Information figure (similar to Fig. S7) showing M. pyrifera biomass for each site would help clarify the direct influence of the overstory on understory macrophytes.

Additionally, Fig. S7 shows substantial variation in P. californica biomass among sites that does not appear to be explained by the temperature gradient (L290-291). In particular, sites shown in Fig. S7B, F, and I exhibit consistently lower biomass prior to the MHW. The authors should clarify whether this pattern may be driven by higher M. pyrifera biomass and associated shading, or by other abiotic or biotic factors. While site-level variation is briefly acknowledged (L278-279), providing more contextual information for each site would help readers understand why macroalgal communities differ so strongly independent of temperature.

Reviewer #2 (Remarks to the Author):

Summary

This manuscript presents a valuable and impressively long-term dataset from the Santa Barbara Channel LTER, spanning 23 years and encompassing two major marine heatwave events. The temporal depth, spatial replication, and taxonomic resolution provide a strong foundation for assessing how understory macroalgal communities respond to acute and repeated thermal stress. The authors document notable shifts in community composition, particularly the decline and recovery of Pterygophora californica and increases in subtropical and geniculate coralline algae, despite relatively stable total biomass and richness. These results are timely and of broad interest given increasing marine heatwave frequency.

Overall, the study is well executed and data-rich. My main comments focus on framing, synthesis, and presentation, with the goal of helping the authors more clearly convey the ecological insights emerging from this extensive dataset.

Major Comments

1. Clarify framing and expectations
The introduction provides a thorough overview of marine heatwaves and macroalgal responses, but it would benefit from a clearer articulation of the ecological expectations guiding the analyses. In particular, it would help to clarify:
o Whether the study is primarily examining binary responses to heatwaves (presence/absence of events) or graded responses to heatwave duration and intensity.
o How differences among species or functional groups (e.g., thermal tolerance, nutrient sensitivity, life history traits, canopy–understory interactions) inform expectations for community change.
Introducing key taxa and functional groups earlier, especially Pterygophora californica and geniculate coralline algae, would help orient readers before the results are presented.

2. Balance scope and synthesis
The manuscript contains a wealth of analyses, figures, and models that collectively document multiple dimensions of community change. While this richness is a strength, it also makes the central narrative difficult to follow in places. The authors might consider:
o Streamlining the Results to emphasize the most important patterns.
o Integrating some of the interpretive context currently reserved for the Discussion earlier in the Results to aid synthesis.
Relatedly, some results appear at the end of the Introduction; relocating these to the Results section would improve flow and conventional structure.

3. Opportunities for deeper ecological interpretation

The Discussion highlights several compelling patterns, particularly the loss and recovery of Pterygophora and sustained increases in subtropical and coralline taxa. These findings could be strengthened by:
o Expanding on the ecological mechanisms that may underlie species-specific responses (e.g., temperature–nutrient interactions, canopy effects, site-level environmental variation).
o More explicitly comparing responses among taxa to help readers understand why some groups appear resilient while others respond strongly to heatwaves.
The long-term nature of the dataset offers an excellent opportunity to explore these mechanisms in greater depth.

4. Predictor and explanatory variables

Several figures emphasize proportional biomass changes among thermal groups. While informative, presenting absolute biomass (or explicitly justifying the focus on proportions) would help distinguish true increases from compositional shifts driven by declines in other taxa. Where possible, linking patterns directly to temperature metrics rather than geography would also strengthen interpretation.

5. Journal fit and scope

Finally, I note that the breadth of analyses, number of figures and models, and depth of ecological detail suggest that this work may benefit from a format that allows for a longer manuscript and more expansive discussion. A journal with fewer constraints on length could allow the authors to more fully develop the ecological context, mechanisms, and implications of their findings, which are clearly substantial.

Minor Comments

• Clarify terminology where first introduced (e.g., canopy effects, species insurance).
• Add missing figure labels and legends, and consider modest use of color to improve readability.
• Ensure consistency with journal style for references and formatting.
• Introduce species with brief ecological context where they first appear.

Recommendation

This is a strong and valuable contribution based on an exceptional long-term dataset. With clearer framing, streamlined presentation, and expanded ecological synthesis, potentially in a journal format that accommodates greater depth, the manuscript has the potential to make a significant impact on our understanding of macroalgal community responses to marine heatwaves.

Reviewer #3 (Remarks to the Author):

This study presents a long-term dataset of macroalgae community trends and compares these to multiple large marine heat wave events across the Californian coast. These data come from a range of locations across latitudes and biogeographic gradients, providing broad applicability to the responses observed. This study represents a complete and rigorous study that has been analysed and interpreted well. I can find little to fault this manuscript but recommend that more international context is added to the discussion.

I found this manuscript logical, easy to read, and commended the authors on their efforts.

Specific comments:
Lines 50-53: Life histories are also critical for determining the responses, recovery and resilience of species
Figure 2: no letter label for graph “C”, no legend label for sites.

Discussion:
Discussion requires more detail and exploration. One of the biggest findings of this study is the decline of Pterygophora. What are the implications of this decline? This is a productive species (Miller et al. 2011, L&O) and some of the species which have responded positively may be far less productive (e.g., Coralline algae). Alternatively, some of the invasive species may be more productive (but more disruptive to ecological processes and timing of productivity)?
Furthermore, other studies, particularly from Australia and New Zealand, show declines in Macrocystis associated with elevated temperatures and marine heat waves. While the same changes in this canopy species were not observed in this study, further interpretation of these trends and differences between other locations would be appropriate to elaborate on in the discussion.

** Visit Nature Portfolio's author and referees' website at www.nature.com/authors for information about policies, services and author benefits**

Communications Earth & Environment is committed to improving transparency in authorship. As part of our efforts in this direction, we are now requesting that all authors identified as ‘corresponding author’ create and link their Open Researcher and Contributor Identifier (ORCID) with their account on the Manuscript Tracking System prior to acceptance. ORCID helps the scientific community achieve unambiguous attribution of all scholarly contributions. You can create and link your ORCID from the home page of the Manuscript Tracking System by clicking on ‘Modify my Springer Nature account’ and following the instructions in the link below. Please also inform all co-authors that they can add their ORCIDs to their accounts and that they must do so prior to acceptance.
https://www.springernature.com/gp/researchers/orcid/orcid-for-nature-research

For more information please visit http://www.springernature.com/orcid

If you experience problems in linking your ORCID, please contact the Platform Support Helpdesk.

Version 1:

Decision Letter:

** Please ensure you delete the link to your author home page in this e-mail if you wish to forward it to your coauthors **

Dear Ms Michaud,

Your manuscript titled "Macroalgal community transformation during successive marine heatwaves in southern California kelp forests" has now been seen by the editorial team. We are delighted to say that we are happy, in principle, to publish a suitably revised version in Communications Earth & Environment.

We therefore invite you to revise your paper one last time to edit your manuscript to comply with our format requirements and to maximise the accessibility and therefore the impact of your work.

EDITORIAL REQUESTS:

Please review our specific editorial comments and requests regarding your manuscript in the attached "Editorial Requests Table".

*****Please take care to match our formatting and policy requirements. We will check revised manuscript and return manuscripts that do not comply. Such requests will lead to delays. *****

Please outline your response to each request in the right hand column. Please upload the completed table with your manuscript files as a Related Manuscript file.

If you have any questions or concerns about any of our requests, please do not hesitate to contact me.

SUBMISSION INFORMATION:

In order to accept your paper, we require the files listed at the end of the Editorial Requests Table; the list of required files is also available at https://www.nature.com/documents/commsj-file-checklist.pdf .

OPEN ACCESS:

Communications Earth & Environment is a fully open access journal. Articles are made freely accessible on publication. For further information about article processing charges, open access funding, and advice and support from Nature Portfolio, please visit https://www.nature.com/commsenv/open-access

At acceptance, you will be provided with instructions for completing the open access licence agreement on behalf of all authors. This grants us the necessary permissions to publish your paper. Additionally, you will be asked to declare that all required third party permissions have been obtained, and to provide billing information in order to pay the article-processing charge (APC).

Please use the following link to submit the above items:
Link Redacted
** This url links to your confidential home page and associated information about manuscripts you may have submitted or be reviewing for us. If you wish to forward this email to co-authors, please delete the link to your homepage first **

We hope to hear from you within two weeks; please let us know if you need more time.

Best regards,

Alice Drinkwater, PhD
Associate Editor
Communications Earth & Environment
Consulting Editor
Communications Sustainability

** Visit Nature Portfolio's author and referees' website at <a

href="http://www.nature.com/authors">www.nature.com/authors for information about policies, services and author benefits**

Open Access This Peer Review File is licensed under a Creative Commons Attribution 4.0 International License, which permits use, sharing, adaptation, distribution and reproduction in any medium or format, as long as you give appropriate credit to the original author(s) and the source, provide a link to the Creative Commons license, and indicate if changes were made.
In cases where reviewers are anonymous, credit should be given to 'Anonymous Referee' and the source.
The images or other third party material in this Peer Review File are included in the article's Creative Commons license, unless indicated otherwise in a credit line to the material. If material is not included in the article's Creative Commons license and your intended use is not permitted by statutory regulation or exceeds the permitted use, you will need to obtain permission directly from the copyright holder.
To view a copy of this license, visit https://creativecommons.org/licenses/by/4.0/

REVIEWER COMMENTS:

Reviewer #1 (Remarks to the Author):

This manuscript examines the impacts of two successive marine heatwaves on macroalgal understory community structure. This manuscript covers a highly relevant topic in the environmental sciences, as it focuses on the recovery of the understory macroalgal communities after repeated MHWs, as well as the shifts in subtropical, transitional and introduced species. Although the impacts of MHWs, particularly the "warm blob", have been frequently studied, as well as the time series being used multiple times, this manuscript is novel in the way that the data is analysed and the conclusions presented. The paper is well written and clear throughout, and the statements are supported by the statistical analyses performed.

We thank Reviewer 1 for their thoughtful assessment of our manuscript.

I would, however, like to request additional contextual information for the different sites, particularly regarding overstory macroalgal biomass and how this may have influenced understory biomass. I acknowledge that this study focuses on the understory community structure, but more information on *Macrocystis pyrifera* would be valuable, especially since you found that the understory biomass and richness was negatively associated with *M. pyrifera* biomass (L101-102), for which Fig. S3 and S4 support this. Including an additional Supporting Information figure (similar to Fig. S7) showing *M. pyrifera* biomass for each site would help clarify the direct influence of the overstory on understory macrophytes.

We have modified Fig. S4 to include biomass of *Macrocystis* (solid line) versus total understory biomass (dotted line) across individual sites to provide context for the effects of *Macrocystis* on the understory. Additionally, we have added language to the Results (lines 109-111) to explain that *Macrocystis* biomass is variable both spatially and temporally across sites.

Additionally, Fig. S7 shows substantial variation in *P. californica* biomass among sites that does not appear to be explained by the temperature gradient (L290-291). In particular, sites shown in Fig. S7B, F, and I exhibit consistently lower biomass prior to the MHW. The authors should clarify whether this pattern may be driven by higher *M. pyrifera* biomass and associated shading, or by other abiotic or biotic factors. While site-level variation is briefly acknowledged (L278-279), providing more contextual information for each site would help readers understand why macroalgal communities differ so strongly independent of temperature.

Pterygophora composes the vast majority of understory kelp biomass across study sites (Fig. S18), and *Macrocystis* biomass was not a significant negative predictor of understory kelp biomass in our analysis (Fig. S17, Table 1, Model 9). We have added language to the Results (lines 200-206) to clarify which functional groups were affected by *Macrocystis* biomass. We have also added language in the Discussion (lines 246-250) to explain that variability in environmental conditions across spatial scales may drive these site-level differences in *Pterygophora* biomass (which were explored by Lamy et al. 2018). We also note other potential abiotic and biotic drivers including grazer abundance, substrate availability etc. that may have driven differences in *Pterygophora* biomass prior to heatwave years, though evaluating these specific drivers of site-level variation was outside the scope of our analysis. Finally, we added

additional language in the Supplementary Information under “Site Descriptions” to discuss important contextual information regarding site level differences (line 349, Supplementary Information).

Reviewer #2 (Remarks to the Author):

Summary

This manuscript presents a valuable and impressively long-term dataset from the Santa Barbara Channel LTER, spanning 23 years and encompassing two major marine heatwave events. The temporal depth, spatial replication, and taxonomic resolution provide a strong foundation for assessing how understory macroalgal communities respond to acute and repeated thermal stress. The authors document notable shifts in community composition, particularly the decline and recovery of *Pterygophora californica* and increases in subtropical and geniculate coralline algae, despite relatively stable total biomass and richness. These results are timely and of broad interest given increasing marine heatwave frequency.

Overall, the study is well executed and data-rich.

We thank this reviewer for this assessment of our manuscript.

My main comments focus on framing, synthesis, and presentation, with the goal of helping the authors more clearly convey the ecological insights emerging from this extensive dataset.

Major Comments

1. Clarify framing and expectations
The introduction provides a thorough overview of marine heatwaves and macroalgal responses, but it would benefit from a clearer articulation of the ecological expectations guiding the analyses. In particular, it would help to clarify:
o Whether the study is primarily examining binary responses to heatwaves (presence/absence of events) or graded responses to heatwave duration and intensity.

We added text to the Introduction (lines 74-75) to clarify that we primarily assessed graded responses to heatwave duration and intensity. Linear and generalized linear mixed effects models were used to evaluate whether functional or thermal groups responded to heatwave duration (the proportion of days that were classified as heatwave days per year), which would be classified as a “graded” response. We used CAP and PERMANOVA analyses to evaluate whether there were shifts in community composition during discrete time periods: before the strong heatwave years, during the strong heatwave years, and after the strong heatwave years, which could be considered a more “binary” response.

o How differences among species or functional groups (e.g., thermal tolerance, nutrient sensitivity, life history traits, canopy–understory interactions) inform expectations for community change.
Introducing key taxa and functional groups earlier, especially Pterygophora californica and geniculate coralline algae, would help orient readers before the results are presented.

As suggested by Smale et al. (2020) and the review of macroalgal responses to heatwaves by Straub et al. (2019), the response of species and functional groups can vary in both space and time, making it difficult to predict precisely how species will respond to heatwave events. We added language to the Introduction (lines 53-56) to introduce key functional groups including geniculate coralline algae. The response of understory kelps was previously mentioned (lines 49-51). There are no prior assessments of *Pterygophora californica* responses to marine heatwaves or thermal tolerance to our knowledge, so our predictions regarding this species are based on the thermal tolerance and responses of other kelps.

2. Balance scope and synthesis
The manuscript contains a wealth of analyses, figures, and models that collectively document multiple dimensions of community change. While this richness is a strength, it also makes the central narrative difficult to follow in places. The authors might consider:
o Streamlining the Results to emphasize the most important patterns.

We have made the Results more readable by breaking it into relevant subsections that align with similar subsections in the Methods.

o Integrating some of the interpretive context currently reserved for the Discussion earlier in the Results to aid synthesis.

Although we do some of this, including substantially more interpretative context in the Results would require an integrated Results/Discussion section instead of separate Results and Discussion.

Relatedly, some results appear at the end of the Introduction; relocating these to the Results section would improve flow and conventional structure.

We highlight our most important findings in the concluding paragraph of the Introduction in lines 76-86, and we describe these findings in more detail in the Results. We provide additional context for these major findings in the Discussion to further emphasize their importance.

3. Opportunities for deeper ecological interpretation

The Discussion highlights several compelling patterns, particularly the loss and recovery of Pterygophora and sustained increases in subtropical and coralline taxa. These findings could be strengthened by:
o Expanding on the ecological mechanisms that may underlie species-specific responses (e.g., temperature–nutrient interactions, canopy effects, site-level environmental variation).

We added language to the Discussion (lines 246-250) to clarify the importance of site-level environmental variability in contributing to understory metapopulation dynamics as shown by Lamy et al. (2018).

o More explicitly comparing responses among taxa to help readers understand why some groups appear resilient while others respond strongly to heatwaves.

The long-term nature of the dataset offers an excellent opportunity to explore these mechanisms in greater depth.

We added a comparison of responses across functional groups and thermal groups of understory algae to the Discussion (lines 222-232).

4. Predictor and explanatory variables

Several figures emphasize proportional biomass changes among thermal groups. While informative, presenting absolute biomass (or explicitly justifying the focus on proportions) would help distinguish true increases from compositional shifts driven by declines in other taxa.

Figure 3 presents changes in proportional biomass among thermal groups to illustrate relative compositional shifts at each of the sites. This figure is included to show that community composition shifted across the thermal gradient, particularly at the warmest, easternmost sites. Because total understory biomass differs substantially among sites, it would be difficult to clearly compare compositional shifts across sites in plots of biomass. We did, however, present the absolute biomass changes over time of introduced (Fig. S15), subtropical (Fig. S12), and transitional (Fig. S11) groups to complement the inclusion of the proportional changes by thermal groups. Additionally, we also included temporal changes in absolute biomass of each of the thermal groups in Fig. S10 to further complement the proportional data presented in Figure 3.

Where possible, linking patterns directly to temperature metrics rather than geography would also strengthen interpretation.

We have assessed temperature as a predictor in all linear and generalized linear mixed effects models. Site-specific proportions of heatwave days per year was included as a continuous predictor of understory biomass responses across groups.

5. Journal fit and scope

Finally, I note that the breadth of analyses, number of figures and models, and depth of ecological detail suggest that this work may benefit from a format that allows for a longer manuscript and more expansive discussion. A journal with fewer constraints on length could allow the authors to more fully develop the ecological context, mechanisms, and implications of their findings, which are clearly substantial.

We note that this comment (along with others) appears to have been generated using AI. Unlike other suggestions, we did not think this one was useful and consequently we did not alter our manuscript to accommodate it.

Minor Comments

• Clarify terminology where first introduced (e.g., canopy effects, species insurance).

We did not use the term “canopy effects”. We clarified the species insurance concept in the Discussion (lines 240-242).

• Add missing figure labels and legends, and consider modest use of color to improve readability.

We added figure labels that were missing from Figure 1 and Figure 2. We used color where appropriate in Figures 1-3 and we think that Figure 4 would not benefit from color.

• Ensure consistency with journal style for references and formatting.

We used formatting that is consistent with Nature journals which is the format requested by *Communications Earth & Environment*.

• Introduce species with brief ecological context where they first appear.

It is unclear what the reviewer means by ecological context in this case.

Recommendation

This is a strong and valuable contribution based on an exceptional long-term dataset. With clearer framing, streamlined presentation, and expanded ecological synthesis, potentially in a journal format that accommodates greater depth, the manuscript has the potential to make a significant impact on our understanding of macroalgal community responses to marine heatwaves.

We thank this reviewer for their assessment, though we disagree that this manuscript requires a longer format.

Reviewer #3 (Remarks to the Author):

This study presents a long-term dataset of macroalgae community trends and compares these to multiple large marine heat wave events across the Californian coast. These data come from a range of locations across latitudes and biogeographic gradients, providing broad applicability to the responses observed. This study represents a complete and rigorous study that has been analysed and interpreted well. I can find little to fault this manuscript but recommend that more international context is added to the discussion.

I found this manuscript logical, easy to read, and commended the authors on their efforts.

We thank this reviewer for their thoughtful analysis.

Specific comments:
Lines 50-53: Life histories are also critical for determining the responses, recovery and resilience of species

Life history is now included as a driver of differential responses to heatwaves (line 51).

Figure 2: no letter label for graph “C”, no legend label for sites.

Thank you for catching this; we have updated this figure to include the proper labels.

Discussion:

Discussion requires more detail and exploration. One of the biggest findings of this study is the decline of Pterygophora. What are the implications of this decline? This is a productive species (Miller et al. 2011, L&O) and some of the species which have responded positively may be far less productive (e.g., Coralline algae). Alternatively, some of the invasive species may be more productive (but more disruptive to ecological processes and timing of productivity)?

We have added some implications of the decline of *Pterygophora* in contrast to other potentially less productive species (Lines 299-304). Additionally, we have expanded the discussion of the implications of increases in corallines for kelp forest grazers (lines 303-308).

Furthermore, other studies, particularly from Australia and New Zealand, show declines in *Macrocystis* associated with elevated temperatures and marine heat waves. While the same changes in this canopy species were not observed in this study, further interpretation of these trends and differences between other locations would be appropriate to elaborate on in the discussion.

To the Discussion we added a description of the response of *Macrocystis* within the SBC and how it differed from other locations within this species’ range including southern California, Baja California (Cavanaugh et al. 2019, Arafeh-Dalmau et al. 2019), Australia (Johnson et al. 2011, Layton et al. 2020) and New Zealand (Tait et al. 2015, Lines 233-237).

Macroalgal community transformation during successive marine heatwaves in southern California kelp forests

Kristen M. Michaud*, Daniel C. Reed, and Robert J. Miller

Marine Science Institute, University of California Santa Barbara, Santa Barbara, CA 93106

*Corresponding author: kmmichaud@ucsb.edu

Abstract

Understanding the acute and longer-term effects of marine heatwaves on coastal ecosystems is critical for predicting their response to future warming. We used a 23-year time series that included two marine heatwaves (the 2014-2015 "warm blob" and 2018) to investigate their effects on understory macroalgae at nine giant kelp forests in the Santa Barbara Channel. We observed little change in macroalgal biomass and species richness in response to the heatwaves, but significant changes in community composition, including the loss of the dominant kelp *Pterygophora californica* and shifts towards subtropical and introduced species. Understory communities were surprisingly resilient, as community composition largely returned to pre-heatwave conditions within a few years. However, there were some exceptions. In particular, subtropical species and geniculate coralline algae increased in abundance in the decade following the heatwaves, suggesting that macroalgal communities are likely to shift in the long term if marine heatwaves increase in frequency and intensity.

Main Text

Commented [A1]: Canopy is not introduced prior, make clear what this means

Commented [A2]: Make clear if your results speak to a binary response or a metered response (heatwave, yes-no or strength and duration of the heatwave)

Temperature is fundamentally important in determining the distribution and diversity of marine organisms [1–4] over ecological and evolutionary time scales [5]. As global ocean temperature has significantly increased over the last century [6,7], many species have shifted both poleward and deeper to remain within their thermal tolerance limits [8–10]. In conjunction with this longer-term trend, acute warming events (i.e., marine heatwaves) can cause abrupt and drastic shifts in marine communities [11,12]. Increases in mean ocean temperatures combined with regionally-specific climate drivers, including ocean-atmospheric processes and ENSO dynamics in the Pacific, have coincided with increased frequency and intensity of marine heatwaves over the last century [13,14]. These extreme events are projected to become more frequent in many regions under future warming scenarios [14,15], potentially impacting marine ecosystems and the goods and services they provide.

The ecological and socioeconomic consequences of marine heatwaves can be severe, threatening biodiversity and altering ecosystem structure and function [11,16–18]. Such impacts can be driven by both range expansions and species losses [10,19–21]. Increases in warm-tolerant invasive species have caused significant changes to community composition following heatwave disturbances [22,23]. By contrast, declines of fished species during periods of acute warming have caused substantial losses in commercial and recreational fisheries [24,25]. Marine heatwaves can be particularly damaging when extended warming threatens coastal foundation species such as kelps, seagrasses, mangroves, and hermatypic corals, which increase habitat complexity, biodiversity, productivity, and ecosystem stability (reviewed in [11,12]). For example, mass coral bleaching events have been accompanied by functional homogenization of fish communities as coral-associated species are lost [26]. Such shifts in species abundances may subsequently alter interspecific interactions, with unexpected consequences for marine communities [27].

Commented [A3]: Check journal style for references with or without spaces

Commented [A4]: What kind of species

Commented [A5]: What kind of shifts?

Commented [A6]: Unusual to refer to ecosystems as providing goods

Commented [A7]: In general I would appreciate more specifics from these types of statements (who, what, where). Even just one example

Commented [A8]: What is the contrast? Increase vs decrease? But they both result in a negative outcome. Perhaps the topic sentence can be more direct. Consequences of marine heatwaves can both increase and decrease population size

Commented [A9R8]: Also explain why you focus only on fished species and not others, are they not impacted?

Commented [A10]: Just say corals

Shallow temperate reefs dominated by macroalgae are highly productive ecosystems, hosting a diverse suite of species that provide important ecosystem goods and services to coastal communities [28–30]. The response of macroalgae to marine heatwaves varies among species and functional groups; some are resistant while others, including many large canopy-forming kelps, display decreased physiological performance or abundance declines (reviewed in [31]). Differences in thermal tolerance may confer advantages to some species and functional groups, resulting in predictable shifts in benthic community structure during heatwaves. Species-specific responses can also vary over both time and space [32–34], making it difficult to predict how temperate macroalgal communities will respond to future heatwaves.

Commented [A11]: Marine heatwaves as hot spots of climate change and impacts on biodiversity and ecosystem services

See also

Commented [A12]: Such as

Commented [A13]: But you just said they were predictable

Commented [A14R13]: Also try and inject the problem statement here. It is alluded to in this sentence but needs to be expanded on given the previous contradicting sentence

Long-term observations are crucial for identifying both abrupt and gradual changes in community composition in response to marine heatwaves [12]. The Santa Barbara Coastal Long Term Ecological Research (SBC LTER) program has measured species-specific biomass of macroalgae annually at nine rocky reef sites off the mainland coast of the Santa Barbara Channel since 2001 to detect such patterns and identify the processes driving them. Two recent marine heatwaves, the first and strongest in 2014-2015 (the warm blob) and the second in the summer of 2018, manifested as strong positive temperature anomalies along coastal California including the Santa Barbara Channel [35–37]. During the 2014-2015 heatwave, which occurred across the entire northeast Pacific [38], intense surface stratification limited upwelling efficiency, allowing anomalously high ocean temperatures to persist for two years, from the winter 2014 to the winter of 2016 [35]. Three years later, another marine heatwave occurred off southern California and Baja California, bringing anomalously warm waters to the coastal ocean in the summer and fall of 2018 [36].

Commented [A15]: This section oculd be condensed if you add text to the other sections as suggested

We used long-term monitoring data from the SBC LTER to explore the initial impact of these two marine heatwaves on subtidal macroalgal community structure and the legacy left by them over the past decade. We evaluated whether the response of macroalgae was consistent across taxonomic and functional groups and assessed whether warm-tolerant taxa and introduced species increased and persisted after the heatwaves. We show dramatic shifts in community composition during heatwave years including rapid declines in understory kelps and increases in subtropical and introduced species. Macroalgal communities, however, were resilient, and understory kelps recovered quickly after heatwave conditions subsided. Contrary to predictions from laboratory studies [39], the calcareous species *Corallina officinalis* increased during the heatwaves, and has remained more abundant since, highlighting the importance of *in situ* assessments of thermal tolerance under realistic heatwave conditions. Both acute and gradual increases in multiple species of geniculate coralline algae (GCA) including *Corallina officinalis, Amphiroa beauvoisii*, and *Calliarthron cheilosporioides,* suggest that this functional group may be particularly successful under warmer conditions with more frequent marine heatwaves.

Commented [A16]: The intro needs some basis for the hypothesis. You mention different things change differently but we need some mechanisms to make and test predictions. Can be elaborated on in the preceeding paragraphs

Commented [A17]: Unusual to have the results at the end of the introduction? You would normally set up your research q's and how you tested them (partly done already)

Results

Identifying Thermal Anomalies and Marine Heatwaves

Both the 2014-2015 and 2018 heatwave periods sustained large positive bottom temperature anomalies, particularly during the summer and fall months (Fig. 1A). Anomalies were highest in fall of 2015 when the average monthly anomaly exceed +4°C (Fig. 1A). On average across sites, 49.6% (July 2014 - June 2015), 41.6% (July 2015 - June 2016), and 20.6% of days (July 2018 - June 2019) were classified as heatwave days (Fig. S1A). The longest continuous stretches of heatwave conditions were observed from October 2014 to February 2015

Commented [A18]: Good to define the standard definition of a heatwave

(146 days) and from August to October 2015 (71 days). All years since 2013 have had at least one heatwave event.

Commented [A19]: Important for later discussion.

Are you testing these two heatwaves or chronic heatwaves?

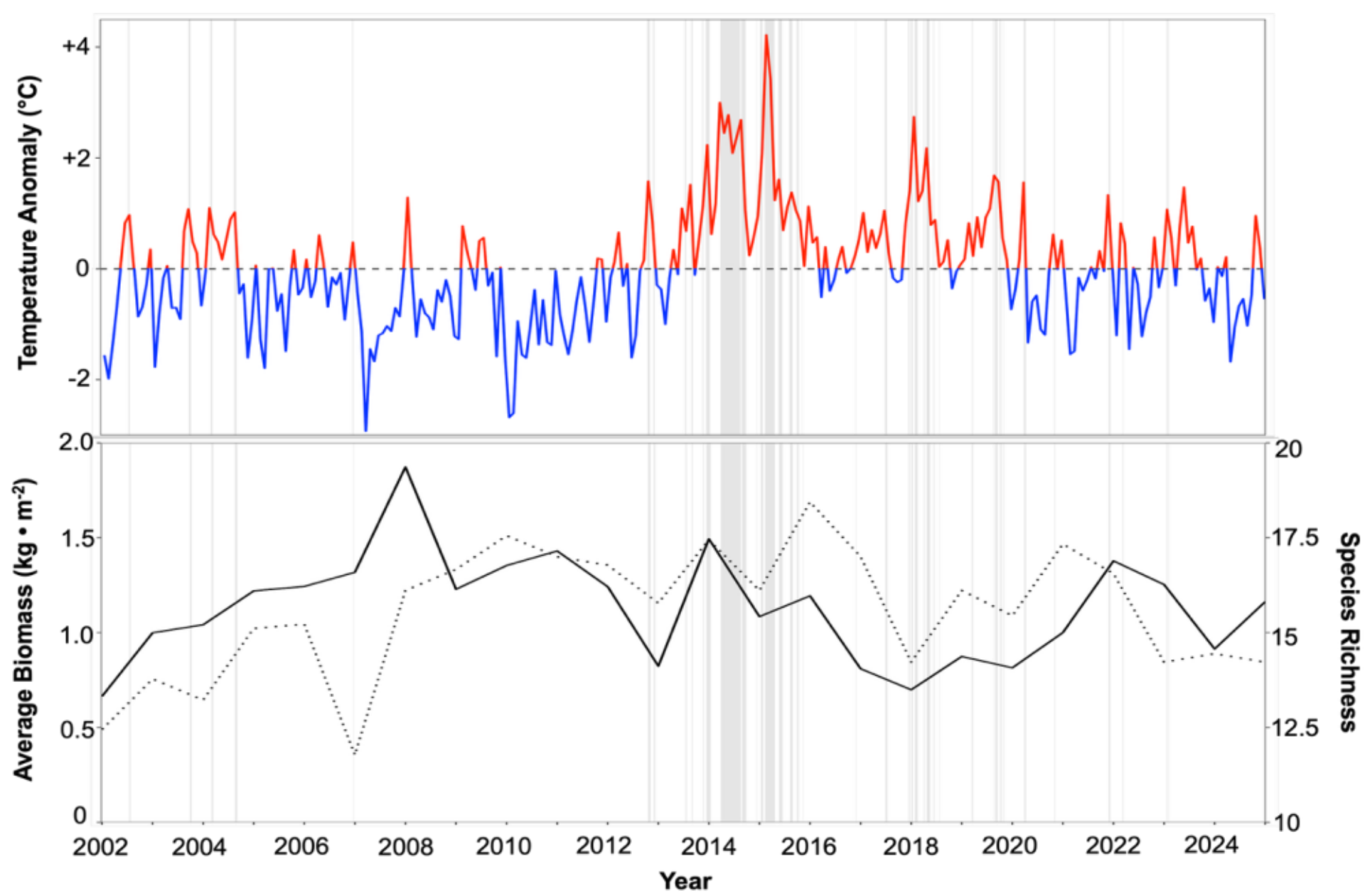

Figure 1. A) Average monthly bottom temperature anomaly across the nine sites. Positive anomalies are shown in red, while negative anomalies are shown in blue. B) Average total understory macroalgal biomass across sites is shown in the solid black line, while average species richness is shown in the dashed black line. Heatwave periods are shown in grey shading. Tick marks on the x-axis signify July (the month in which sampling occurred).

Commented [A20]: Please add a legend

Reef-Wide Shifts in Community Composition During Marine Heatwaves

Total understory macroalgal biomass and species richness during and after the 2014-2015 heatwave remained within or above the range observed before the heatwave (2002-2014) and increased from 2018 to 2022 following the 2018 heatwave (Fig. 1B). Heatwave duration was not a significant predictor of understory biomass or richness over time. Instead, understory biomass

and richness were negatively associated with the biomass of the major overstory canopy species, *Macrocystis pyrifera* (Table 1, Models 1 & 2, Figs. S2 & S3), which was also unresponsive to heatwave duration (Table 1, Model 3, Figs. S4 & S5).

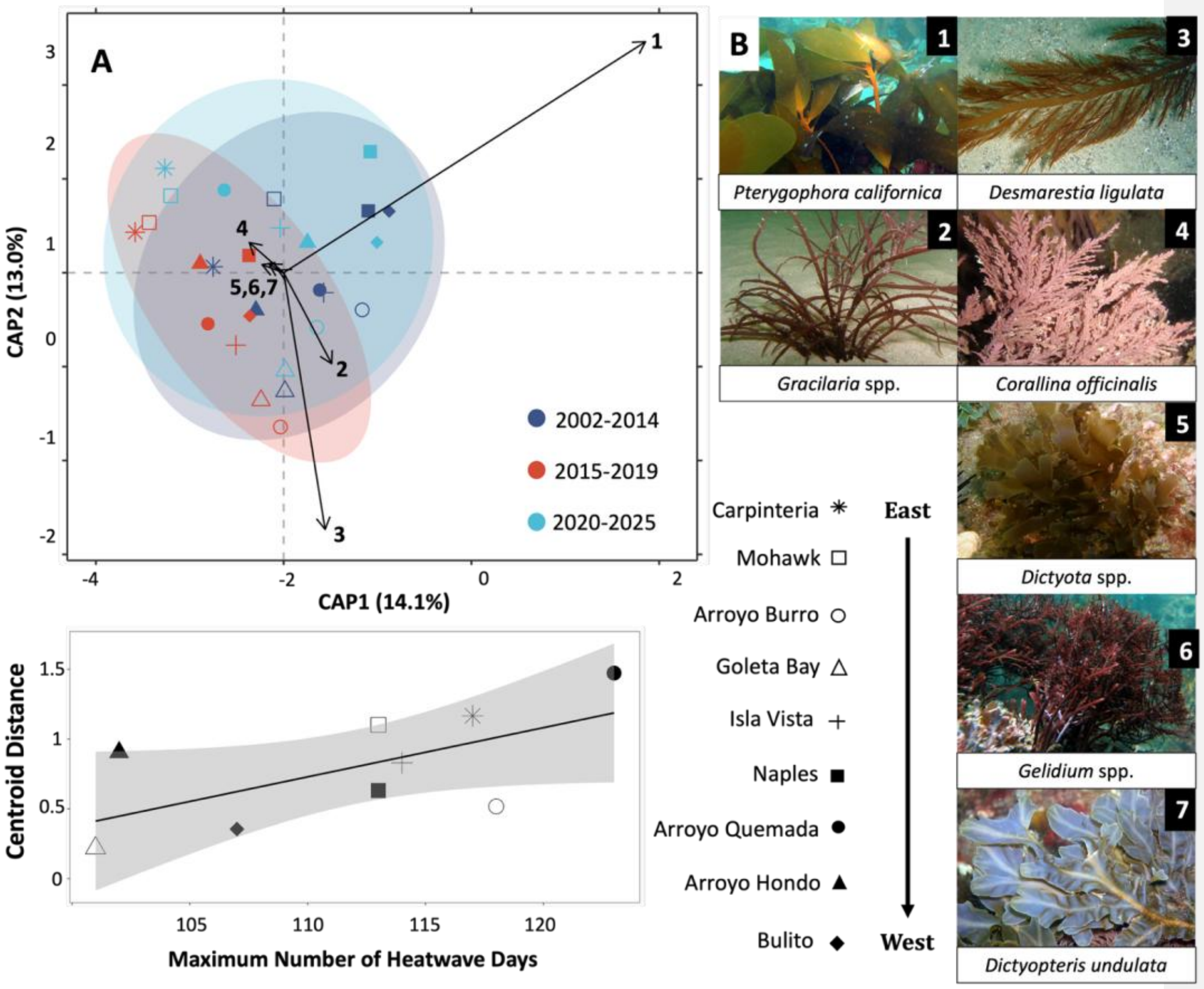

Figure 2. A) Canonical Analysis of Principal Coordinates for community composition across the nine sites and three time periods (R^2=0.488). The ordination was constrained by the interaction between site and time period (F=5.916, p<0.001) and points represent centroids for each site and time period interaction. The ellipses represent the spread of all points during each time period across all sites and years (Fig. S6). Shapes represent sites.

Numbered arrows correspond to the species that contributed the most to community dissimilarity over time with $r>0.20$ which are shown in (B). C) Correlation between the difference in average community composition at a site before (2002 to 2014) vs. after (2020 to 2025) the heatwave years (2015-2019) against the maximum number of days per year when bottom temperature exceeded the site-specific 90th percentile temperature threshold. Community differences were estimated using the distances in the average community centroids for each site from A) for each time period. The solid line represents the linear fit and 95% confidence interval in gray shading (Supplemental Table 1).

Although total understory biomass remained high, there were significant differences in community composition before (2002-2014), during (2015-2019), and after (2020-2025) the strong heatwave years (PERMANOVA, $F=9.8326$, $p<0.001$; Fig. 2A). Biomass of the dominant understory kelp *Pterygophora californica* was negatively associated with heatwave conditions, while biomass of *Corallina officinalis, Dictyota* spp., *Dictyopteris undulata,* and *Gelidium* spp. were positively associated with heatwave and post-heatwave conditions, particularly at the warmest sites (Fig. 2A).

Pterygophora biomass declined sharply to near zero at all sites during the 2014-2015 heatwave, reaching a 23-year minimum in 2016 (Fig. 4B, Fig. S7; IndVal=0.971, $p<0.001$). Biomass of this species was consistently high at the coolest, westernmost site, Bulito, until the 2014-2015 heatwave (Fig. S7A). Its recovery began in 2018 at the coolest sites (e.g., Bulito and Naples) before progressing to the warmer sites beginning in 2020 (Fig. S7). The loss of *Pterygophora* accounted for the largest shifts in community composition during heatwave years (Fig. 2A).

Commented [A21]: Perhaps this text is better located in the main text as opposed to a caption?

Commented [A22R21]: Or large parts of it

Commented [A23R21]: C is also not labeled

Commented [A24R21]: It may be helpful to add a sub label that reads “community difference/heterogenity” - to centroid distance

Commented [A25]: Please double check me here but I do not believe we should infer increases/decreases from the directional arrows on the PC analysis

Commented [A26]: I understand you are writing for a general audience in a wide scope journal but I feel the ecology has been lost. At no point before this do we get an understanding of Pterygophora or how we might expect it to react to heatwaves.

I would like to see a bit more ecology to contexualize these results

Commented [A27]: Skipped figure 3?

There was a positive correlation between the extent of warming and the degree to which community composition shifted over time as sites with more anomalously warm days in a given year exhibited larger shifts than those with fewer anomalously warm days (Fig. 2C, Table S1). Site specific shifts in species composition were also positively correlated with heatwave intensity from July of 2018 to June of 2019 (Table S1).

Community Homogenization During and Following Marine Heatwaves

Prior to heatwave conditions, sites differed significantly in composition across the thermal gradient (ANOSIM R=0.497, p=0.001, Fig. 2A), though the two easternmost sites exhibited the highest proportion of subtropical species (Fig. 3H-I). The proportional biomass of subtropical species increased across most sites during the heatwave years, but this increase was the highest at the easternmost sites where their biomass increased from 7.0% to 25.7% at Mohawk and from 19.1% to 37.7% at Carpinteria (Fig. 3).

Commented [A28]: Why proportional and not absolute

Commented [A29R28]: Or ideally both

Commented [A30]: Relate to temp, not geography if possible

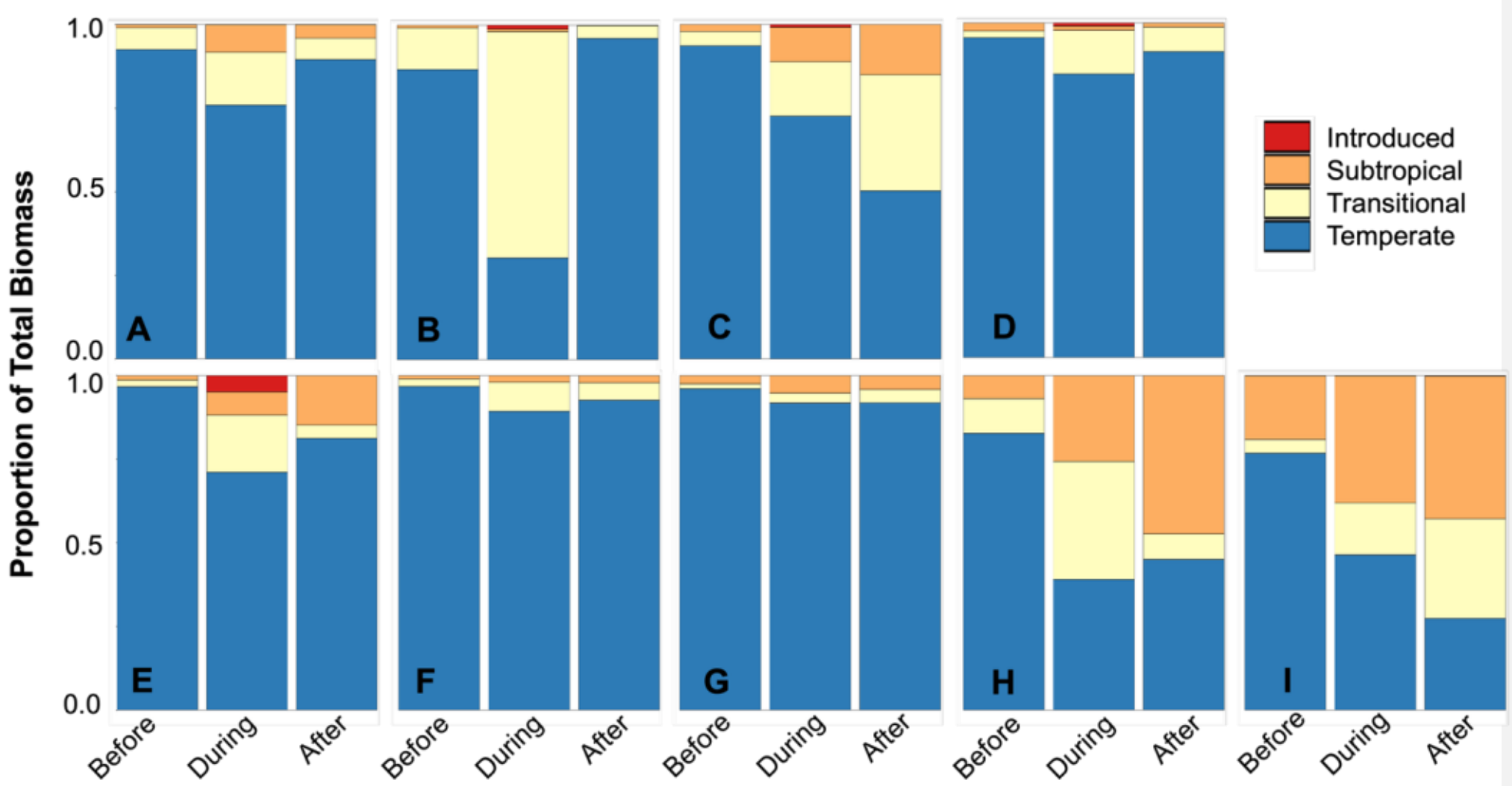

Figure 3. Average proportion of total understory biomass within each thermal group by time period, before the heatwave years (2002-2014), during the heatwave years (2015-

2019), and after (2020-2023), across the thermal gradient from West to East at A) Bulito, B) Arroyo Hondo, C) Arroyo Quemada, D) Naples, E) Isla Vista, F) Goleta Bay, G) Arroyo Burro, H) Mohawk, and I) Carpinteria reefs.

Sites remained significantly different from one another in composition during (ANOSIM, R=0.580, p=0.001) and following (ANOSIM, R=0.663, p=0.001, Figs. 2A & 3) the strong heatwave years, suggesting that community composition did not converge across the thermal gradient in response to more frequent heatwaves. However, composition became more similar at three sites: Carpinteria, Mohawk, and Arroyo Quemada (Fig. 2A). Carpinteria and Mohawk exhibited the highest proportion of subtropical species in total biomass during and after the heatwave periods (44.7% and 50.7%, respectively), while the proportion of transitional species increased during and after the heatwave years at Arroyo Quemada (16.2% to 41.9%) and Carpinteria (15.6% to 26.9%, Fig. 3). The contribution of temperate species returned to pre-heatwave levels at most sites except for Arroyo Quemada, Mohawk and Carpinteria, where temperate species only contributed 41.5%, 40.1%, and 28.3%, respectively, after the strong heatwave years (Fig. 3).

Thermal Group Shifts During Marine Heatwaves

Heatwave duration was a significant positive predictor of subtropical species biomass over time (Table 1, Model 4, Fig. S8). Transitional species biomass increased with increasing heatwave duration, though this effect was not significant (Table 1, Model 6, Fig. S9). Both groups increased in biomass steadily during the strongest heatwave years (Fig. S10), though increases of transitional species beginning in 2015 were driven largely by the dominant brown fucoid *Stephanocystis osmundacea* and the GCA species *Calliarthron cheilosporioides* (Fig.

Commented [A31]: Better to represent visually here and put the table in the supplement?

Commented [A32R31]: There is also just a lot going on in that table, is there some way you can make it more intuitive (colour code, symbols, etc)

Table 1. Model results for all generalized linear models, generalized linear mixed effects models, and linear mixed effects models. Degrees of freedom reported are residual degrees of freedom. Significant effects are bolded. * signifies effects with p-values between 0.05 and 0.10.

Model	Response	Predictors	DF	β	95% CI	*SE*	*z*	*p*
1	Total understory biomass	(Intercept)	179	33.188	[29.487, 36.888]	1.888	17.577	**<0.001**
		Macrocystis biomass		-1.635	[-3.061, -0.209]	0.728	-2.248	**0.025**
	**Random Effects**		**Groups**	**Correlation**	**Observations**			
		Year|Site	8	0.89	184			
2	Total understory richness	(Intercept)	178	16.004	[14.105, 17.904]	0.969	16.514	**<0.001**
		Proportion of Heatwave Days		2.782	[-1.964, 7.528]	2.422	1.149	0.251
		Macrocystis biomass		-0.990	[-1.608, -0.372]	0.315	-3.141	**0.002**
	**Random Effects**		**Groups**	**Correlation**	**Observations**			
		Year|Site	8	0.90	184			
3	*Macrocystis* biomass	(Intercept)	179	-1.321	[-1.793, -0.868]	0.232	-5.694	**<0.001**
		Proportion of Heatwave Days		0.944	[-0.215, 2.101]	0.563	1.677	0.094 *
	**Random Effects**		**Groups**	**Correlation**	**Observations**			
		Year|Site	8	0.95	184			
4	Subtropical species biomass	(Intercept)	177	3.737	[3.043, 4.430]	0.354	10.560	**<0.001**
		Proportion of Heatwave Days		1.305	[0.341, 2.269]	0.492	2.653	**0.008**
		Macrocystis biomass		-0.294	[-0.429, -0.160]	0.069	-4.279	**<0.001**
	**Random Effects**		**Groups**	**Correlation**	**Observations**			
		Year|Site	8	0.96	184			
5	Subtropical species biomass	(Intercept)	179	-135.098	[-170.253, -99.942]	17.937	-7.532	**<0.001**
		Year		0.069	[0.052, 0.086]	0.009	7.759	**<0.001**
	**Random Effects**		**Groups**	**Observations**				
		Site	8	184				
6	Transitional species biomass	**Conditional Model**	177					
		(Intercept)		-2.041	[-2.649, -1.434]	0.310	-6.584	**<0.001**
		Proportion of Heatwave Days		1.323	[-0.073, 2.719]	0.712	1.857	0.063 *
		Macrocystis biomass		0.171	[-0.046, 0.389]	0.111	1.542	0.123
		**Dispersion Model**						
		(Intercept)		2.514	[2.067, 2.961]	0.228	11.019	**<0.001**
		Macrocystis biomass		-0.191	[-0.490, 0.108]	0.153	-1.252	0.211
	**Random Effects**		**Groups**	**Correlation**	**Observations**			
		Year|Site	8	0.86	184			
7	Introduced species biomass	(Intercept)	179	-6.560	[-12.921, -0.200]	3.245	-2.022	**0.043**
		Proportion of Heatwave Days		11.109	[6.827, 15.390]	2.184	5.086	**<0.001**
	**Random Effects**		**Groups**	**Observations**				
		Site	8	184				
8	Temperate species biomass	(Intercept)	179	-1.240	[-1.603, -0.779]	0.198	-6.268	**<0.001**
		Macrocystis biomass		-0.120	[-0.265, 0.031]	0.072	-1.666	0.096 *
	**Random Effects**		**Groups**	**Correlation**	**Observations**			
		Year|Site	8	0.87	184			

Table 1. Continued

Model	Response	Predictors	DF	β	95% CI	*SE*	*z*	*p*
9	Understory kelp biomass	(Intercept)		5.132	[4.320, 5.944]	0.414	12.388	**<0.001**
		Proportion of Heatwave Days	177	-2.562	[-4.118, -1.005]	0.794	-3.225	**0.001**
		Macrocystis biomass		0.153	[-0.022, 0.327]	0.089	1.718	0.086 *
	**Random Effects**		**Groups**	**Correlation**	**Observations**			
		Year|Site	8	0.87	184			
10	GCA biomass	(Intercept)	179	-188.496	[-223.861, -153.131]	18.044	-10.45	**<0.001**
		Year		0.095	[0.078, 0.113]	0.009	10.65	**<0.001**
	**Random Effects**		**Groups**	**Observations**				
		Site	8	184				
11	GCA biomass	(Intercept)		3.222	[2.322, 4.122]	0.459	7.014	**<0.001**
		Proportion of Heatwave Days	177	0.526	[-0.543, 1.594]	0.545	0.964	0.335
		Macrocystis biomass		-0.180	[-0.318, -0.041]	0.071	-2.546	**0.011**
	**Random Effects**		**Groups**	**Correlation**	**Observations**			
		Year|Site	8	0.96	184			
12	Non-kelp brown biomass	(Intercept)	179	5.653	[5.245, 6.060]	0.208	27.205	**<0.001**
		Proportion of Heatwave Days		1.711	[0.686, 2.736]	0.523	3.271	**0.001**
	**Random Effects**		**Groups**	**Observations**				
		Site	8	184				
13	Non-coralline red biomass	(Intercept)	179	-1.629	[-2.082, -1.176]	0.231	-7.049	**<0.001**
		Macrocystis biomass		-0.165	[-0.316, -0.013]	0.077	-2.125	**0.034**
	**Random Effects**		**Groups**	**Correlation**	**Observations**			
		Year|Site	8	0.89	184			
14	Green biomass	(Intercept)		0.482	[-0.342, 1.307]	0.421	1.146	0.252
		Proportion of Heatwave Days	177	1.847	[-0.179, 3.873]	1.034	1.787	0.074 *
		Macrocystis biomass		-1.053	[-1.501, -0.605]	0.229	-4.608	**<0.001**
	**Random Effects**		**Groups**	**Correlation**	**Observations**			
		Year|Site	8	0.86	184			

S11). Subtropical species increased in biomass by 140.0% (2014 to 2016) and 64.5% (2018 to 2019) following the strong heatwave years in 2016 and 2019 (Fig. S12). Biomass subsequently declined in 2017 and 2020 but has increased significantly over time across the thermal gradient (Table 1, Model 5, Fig. S13), predominantly due to increases in the GCA species *Corallina officinalis* (Fig. S12). Increases in two brown algae, *Dictyota* spp. and *Dictyopteris undulata,* and two red algae, *Sarcodiotheca furcata* and *Acrosorium ciliolatum*, were pronounced in 2016 and 2019 (Fig. S12), though these increases were largely restricted to the warmest sites. *Dictyopteris undulata* (IndVal=0.366, p=0.020), *Acrosorium ciliolatum* (IndVal=0.639, p<0.001), *Sarcodiotheca furcata* (IndVal=0.498, p=0.002), and the GCA species *Amphiroa beauvoisii* (IndVal=0.366, p=0.002, Fig. S12) were significantly more abundant during and after heatwave years compared to the period prior to heatwave years.

Heatwave duration was also a significant positive predictor of introduced species biomass (Table 1, Model 7, Fig. S14), which peaked in 2015 during the 2014-2015 heatwave (Figs. S10 & S15). Biomass of the Asian fucoid *Sargassum muticum* increased during the heatwave years (Fig. S15) and was significantly more abundant from 2015-2019 than before or after the strong heatwave years (IndVal=0.457, p<0.001). Its less abundant congener *Sargassum horneri* was only observed at the warmest site, Carpinteria, where its biomass was significantly higher after the strong heatwave years compared to before and during heatwave years (IndVal=0.445, p<0.001, Fig. S15). There was no effect of heatwave duration on temperate species, which comprise the majority of surveyed species (Table 1, Model 8, Fig. S16).

Functional Group Susceptibility to Marine Heatwaves

Biomass within some, but not all, functional groups shifted during strong heatwave years (Fig. 4A). Heatwave duration was a significant negative predictor of understory kelp biomass

Commented [A33]: Of what?

Commented [A34]: At this point I am feeling a bit overwhelmed with information. We are up to supplemental figure 16. And as mentioned, the introduction has not introduced these different species and the expected outcomes. As a result, I am finding it difficult to make sense of the results and their implications.

(Table 1, Model 9, Fig. S17), which is composed primarily of the dominant species *Pterygophora californica* (Fig. S18). Biomass declined during the 2014-2015 heatwave,

Commented [A35]: Maybe add colour to help differentiate?

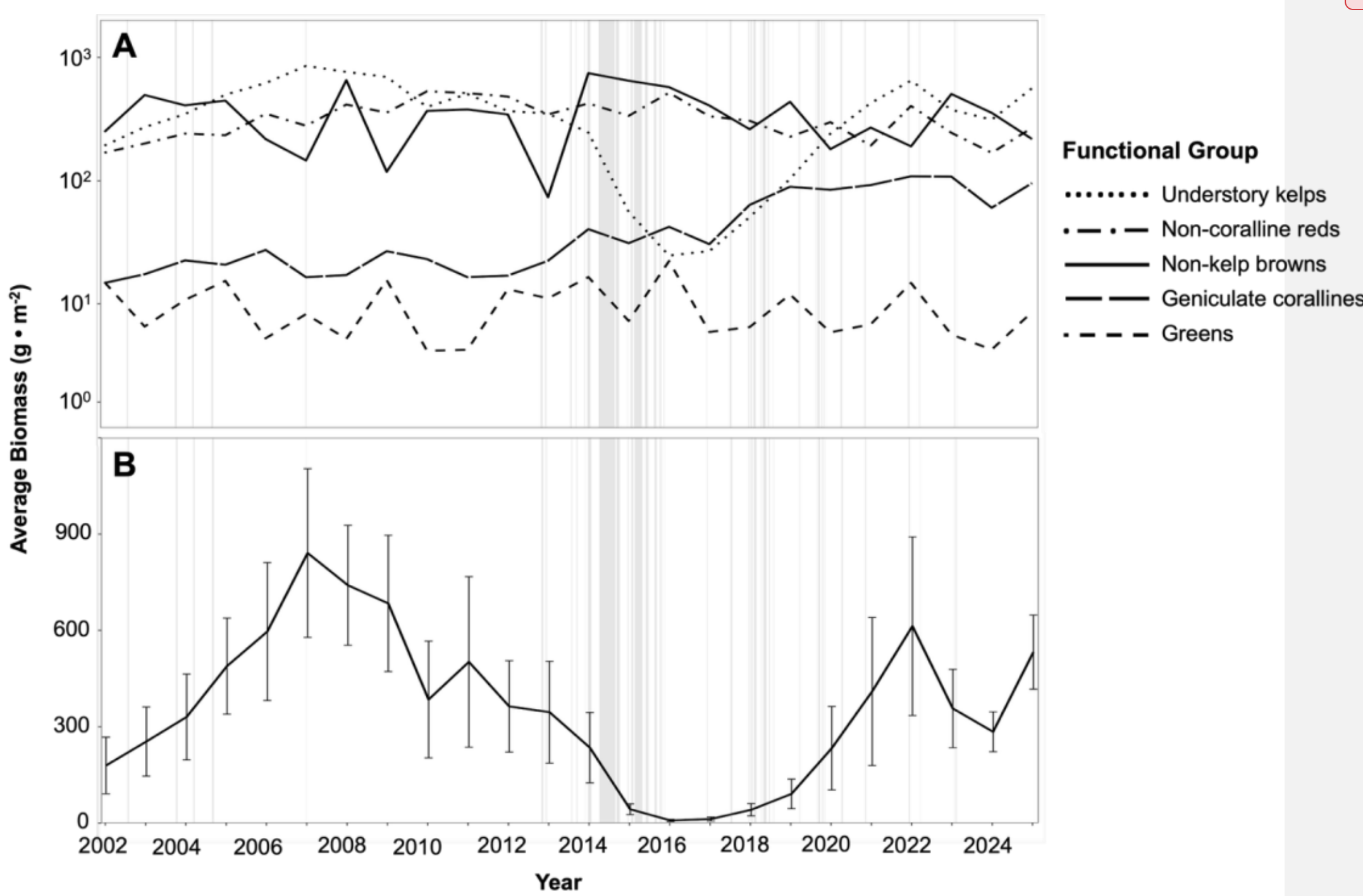

Figure 4. Mean annual biomass of understory macroalgae averaged across sites by A) functional group and B) *Pterygophora californica* only. Biomass is represented on a log scale for panel A. Error bars represent standard error. Heatwave days are shown in grey shading. Tick marks on the x-axis signify July (the month in which sampling occurred).

reaching a minimum in 2016 and remaining low until after the 2018 heatwave (Fig. 4B, Fig. S18). The biomass of geniculate coralline algae (GCA), particularly *Corallina officinalis, Calliarthron cheilosporioides,* and *Amphiroa beauvoisii,* increased significantly over time, particularly during and after 2018, though this was not affected by heatwave duration (Table 1, Models 10 & 11, Figs. 4A, S19, S20). Heatwave duration was a significant positive predictor of

non-kelp brown algal biomass but was unrelated to the biomass of non-coralline red or green algae (Table 1, Models 12-14, Figs. S21 – S23).

Commented [A36]: Perhaps personal opinion but I do not think a paper should have 23 supplemental figures alongside 14 models. I love what you are presenting and your study but it is too much to ingest in such a short review.

Perhaps the paper would be better suited as a longer, experimental paper as opposed to a communication piece?

Discussion

There were significant shifts in understory macroalgal community composition across the mainland coast of the Santa Barbara Channel during the severe and prolonged 2014-2015 heatwave and the subsequent 2018 heatwave. Though richness and biomass remained relatively unchanged during the most severe heatwave conditions, community composition shifted considerably across sites and increases in geniculate coralline and subtropical species persisted over time. The greatest changes in composition occurred at sites where thermal anomalies were most persistent and where bottom temperatures were the highest in 2018, suggesting that the duration of heatwaves, particularly during the warmest months, will affect the degree to which communities respond to future disturbances.

Commented [A37]: Some sites?

The most striking impact of the 2014-2015 heatwave was the loss of the dominant understory kelp, *Pterygophora californica*. During that period, we observed the crowns of this species turning black and disintegrating (Miller, pers. obs.). High species insurance in this system that involved subsequent increases in other understory macroalgal species, particularly non-kelp browns, compensated for the loss of *Pterygophora,* resulting in little net change in total understory biomass and richness during strong heatwave years [40]. In contrast to other locations where losses of canopy forming kelps persisted following heatwave conditions [33,41,42], *Pterygophora* populations recovered rapidly within five years following the heatwave period.

Commented [A38]: Have not heard this term before. Is it common? If not, perhaps rephrase

Commented [A39]: Is it maybe a canopy vs sub effect? Or species specific, good to elaborate here in the discussion

Commented [A40R39]: Also what and where again

Pterygophora recovery was strongest at Bulito and Naples, the sites with the largest and least variable pre-heatwave populations (Fig. S6). The subsequent 2018 heatwave, although it may have slowed recovery, was not associated with net declines at these sites (Fig. S6), perhaps

due to differences in the intensity or duration of thermal stress or nutrient availability during the growth season between the two heatwaves. In contrast to the 2014-2015 heatwave where positive temperature anomalies began during the spring months, limiting mixing and nutrient delivery to surface waters [35], the 2018 heatwave began during summer months [36,43] when wind driven upwelling is generally less important for nutrient delivery in the Santa Barbara Channel [44]. Water column chlorophyll-a concentrations were lower during the spring of 2014 and 2015 compared to 2018 [45], suggesting that nutrients may not have been as limiting during the 2018 heatwave. Both the timing and duration of future heatwaves and their subsequent effects on nutrient availability will affect the capacity of *Pterygophora* populations to resist declines or recover from biomass losses, particularly as this species has demonstrated limited reproductive capacity at elevated temperatures [46,47].

Commented [A41]: What about other species?

Also you note elevated temperatuers but previously talk about nutrient limitation.

You need to draw a link between nutrients and Pterygophora

There were significant shifts in the composition of understory macroalgal communities during and after the heatwave years, particularly increases in the abundance of GCA. Local increases in *Corallina officinalis,* the dominant species of GCA at our sites, during strong heatwave years suggest high thermal tolerance in this species. However, laboratory experiments present conflicting evidence depending on the physiological metric measured and thermal treatments; growth rates of *Corallina officinalis* remained unaffected under conditions of up to 28°C in one manipulation [48], while *Corallina officinalis* exhibited decreased physiological performance above a 23°C threshold following 7 weeks of thermal stress in another experiment [39], suggesting drastically different thermal limits. Calcification rates across measured coralline species decline, on average, at thermal extremes +5°C above ambient [49]. During the warmest month of the 2014-2015 heatwave, temperatures reached an average of 21.1°C across the mainland of the Santa Barbara Channel, about +4.2°C above the monthly average. Increases in

Commented [A42]: Different in what context

photosynthetic rate under warmer conditions may have given *Corallina* a competitive advantage in recent years, though future heatwaves above the species' thermal tolerance limits may hinder its success. The subtropical species *Amphiroa beauvoisii* also increased in abundance, particularly during the strongest heatwave years. Similar increases in this species and other subtropical species of GCA occurred in Baja California following the 1997-1998 El Niño [50] and during the 2009-2010 El Niño in the southern Mexican Pacific where *Amphiroa* was observed overgrowing live corals [51]. Thus far, populations of *Amphiroa* have remained restricted to the warmest site in the SBC, though increases in geniculate corallines suggest this functional group may be particularly successful during future heatwaves either due to high thermal tolerance, high phenotypic plasticity or life history traits that support rapid colonization [49].

Commented [A43]: How does this response compare to other species?

Commented [A44R43]: What are their thermal tolerances

Commented [A45]: Given the structure of the paper, I feel we also did not get a good sense of the sites. Where are warmest, highest nutrients, wave exposure, etc. How do these differences influence the expected results.

Increases in introduced species were mostly restricted to strong heatwave years. Prior observations in La Jolla, CA during the 1992-1993 El Niño revealed an exceptionally high recruitment of the annual fucoid *Sargassum muticum* after the loss of two Laminarian kelps, *Egregia* and *Eisenia* [52], suggesting *S. muticum* was able to colonize during periods with high temperatures and with competitive release of less thermal tolerant understory kelps. Laboratory experiments have shown the success of *S. muticum* over native species during heatwave conditions [53], while increases in other nonnative *Sargassum* species have occurred and persisted following the 2014-2015 MHW in Baja California [22] and following the 1997-1998 El Niño in Southern Japan [54]. In contrast, biomass of *Sargassum muticum* peaked in 2015 and subsequently declined, suggesting that this species may have opportunistically established during years with favorable conditions but died back concomitantly with recovery of understory kelps or the return of cooler waters.

Commented [A46]: Species names

Understory communities shifted across sites in the Santa Barbara Channel in response to heatwave conditions; however, high species richness and distinct community composition across the sites suggests there was not a simplification or homogenization of community composition. Site level variation in composition has persisted, and differences in abiotic conditions, reef structure, or biotic interactions across sites likely caused dissimilarity at this scale [55–57]. Understory communities were relatively resilient during strong heatwave years, and rapid recovery of the dominant understory kelp, *Pterygophora,* drove average community composition back towards pre-heatwave conditions. However, increases in biomass of subtropical and GCA species over time suggests that macroalgal communities will continue to shift in response to more frequent and intense marine heatwaves.

Commented [A47]: Lets dig into this! This is the interesting part

Commented [A48R47]: We need causal mechanisms.

Currently the work reads as too descriptive

Commented [A49]: Good to have a bigger picture conclusion

Methods

Study Sites

The mainland coast of the Santa Barbara Channel is characterized by a longitudinal gradient in upwelling intensity and bottom temperature (Fig. 5A), which is largely due to its southward facing coastline and the convergence of the southward flowing California Current and the northward flowing Southern California Countercurrent [58]. This gradient contributes significantly to the variability in composition of macroalgal communities within the SBC LTER domain [56]. Though shifts in macroalgal community composition are more commonly observed at wider temperature gradients (e.g., 2-3°C over spatial scales of hundreds to thousands of kilometers [59,60]), the Santa Barbara Channel offers a unique opportunity to observe the degree to which marine heatwaves alter the community structure of understory macroalgal communities that exist along a narrow thermal gradient of ~1°C (Figs. 5C & S1B). The convergence of

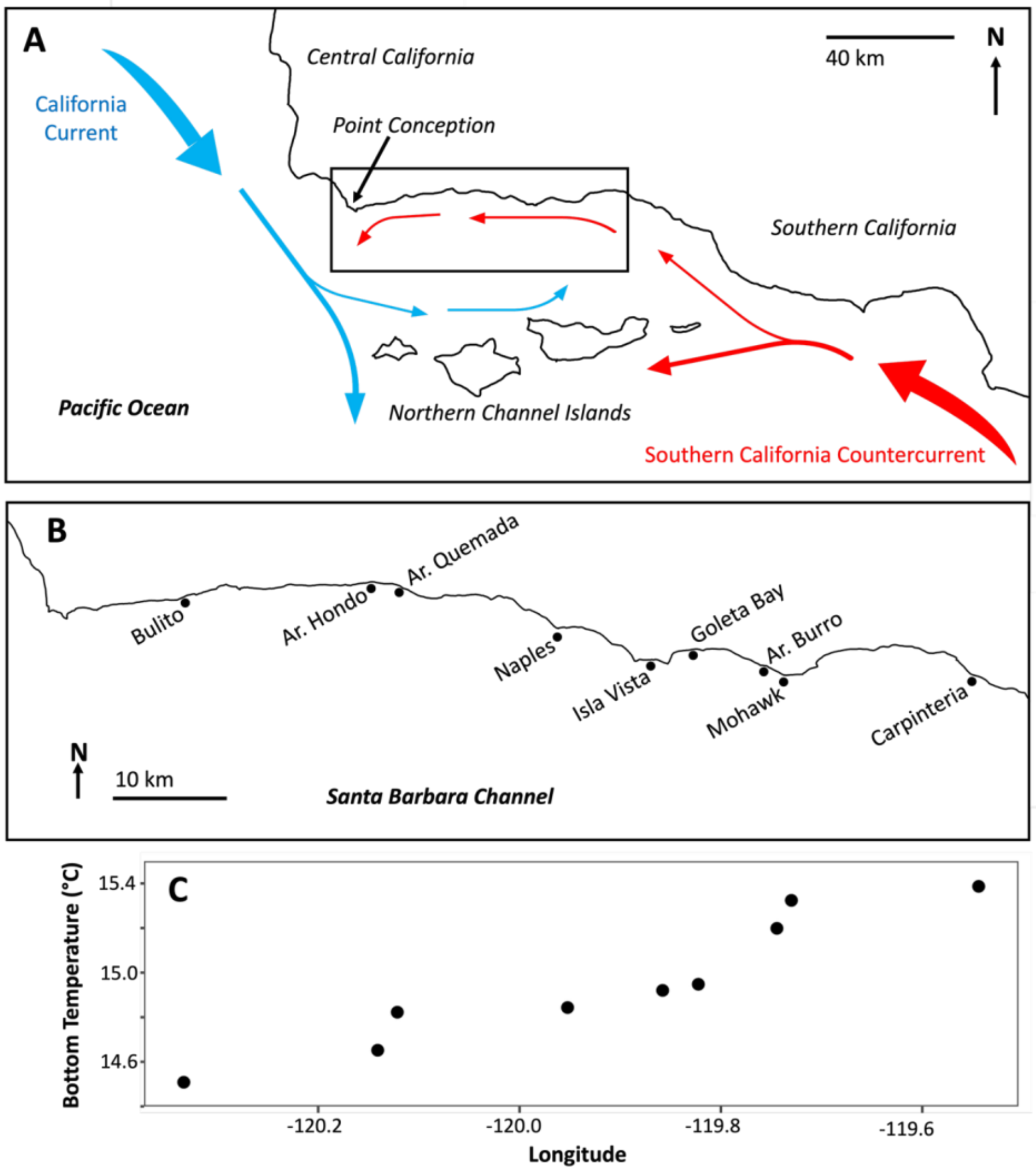

Figure 5. A) Map of the convergence of two major currents flowing within the Santa Barbara Channel. Arrows represent the direction of flow of the northward flowing Southern California Countercurrent (red arrows) and the southward flowing California Current (blue arrows). The rectangular inset shows the locations of sites represented in panel B. B) Map of the 9 sites along the mainland coast of the Santa Barbara Channel

surveyed by the SBC LTER. C) Average annual bottom temperature across the longitudinal gradient from 2003 to 2023 in the Santa Barbara Channel. Points represent the average bottom temperature at each site shown in panel B. The longitudinal scale in panel C is consistent with panel B.

currents forms a strong biogeographic barrier at Point Conception that bounds the flow of propagules from north of the point southward and eastward towards the mainland coast [61]. This boundary may contribute to homogenization of the macroalgal community from east to west following disturbance or in response to gradual warming as colder-affinity species are replaced by subtropical species moving westward.

The SBC LTER program has been monitoring community structure along permanent transects at nine mainland kelp forest reefs annually in July since 2001 [62]. Sites were located across ~ 78 km of the mainland coast of the Santa Barbara Channel (Bulito: 34.46 °N, 120.33 °W, Arroyo Hondo: 34.47 °N, 120.14 °W, Arroyo Quemada: 34.47 °N, 120.12 °W, Naples: 34.42 °N, 119.95 °W, Isla Vista: 34.40 °N, 119.86 °W, Goleta Bay: 34.41 °N, 119.82 °W, Arroyo Burro: 34.40 °N, 119.74 °W, Mohawk: 34.39 °N, 119.73 °W, Carpinteria: 34.39 °N, 119.54 °W; Fig. 5B). The number of permanent transects varied by site from 2 to 8 depending on the total area of the rocky reef [62]. Average transect depth varied between 2.5 m and 11 m below mean lower low water (MLLW) across sites with a mean of 7 m.

Commented [A50]: Key factor influencing composition, how is this controlled for?

Data Collection

Bottom temperature was recorded every 15 minutes at each site since August of 2002 [63] by fixed loggers (Stowaway Onset tidbits, Onset Computer, Bourne, Massachusetts, USA) at an average depth of 7 m MLLW.

Divers used a combination of swath, quadrat, and uniform point contact surveys to estimate abundance and biomass of 62 taxa of understory and canopy forming macroalgae (Supplemental Table 2). Laminarian kelps including *Macrocystis pyrifera, Laminaria farlowii, Pterygophora californica, Eisenia arborea* and the fucoid *Stephanocystis osmundacea* were counted and sized along fixed 40m x 2m transects by divers. The abundances of juvenile kelps and the non-native fucoids *Sargassum horneri* and *Sargassum muticum* were measured by counting and sizing individuals within 6 fixed $1m^2$ quadrats uniformly spaced along the transect. The abundance of smaller species and those that are difficult to distinguish as individuals were measured using percent cover estimates from 80 uniformly spaced points along the transect.

Commented [A51]: This is really quite impressive!

For *Macrocystis pyrifera*, we converted measurements of frond density (no. per transect) to estimates of wet biomass density ($g{\cdot}m^{-2}$) using allometric relationships developed for each month of the year [64]. For all other species, we used taxon-specific allometric relationships to convert measurements of size-specific density or percent cover to wet biomass [62,65,66]. Wet biomass was then averaged across transects to obtain wet biomass by species at each site per year from 2003 to 2025.

Identifying Thermal Anomalies and MHWs

To estimate deviations in bottom temperature from the climatological mean and to identify periods when bottom temperatures were significantly higher than the climatological mean for prolonged periods, we calculated monthly bottom temperature anomalies for each site. Average monthly temperatures were calculated for each site from 2002 to 2025. For each site, we subtracted the average monthly temperature in a given year from the 23-year average temperature for that month. We then averaged the monthly anomalies across all nine sites to produce a monthly anomaly for the SBC region (Fig. 1A).

Commented [A52]: Would this give you positive values for cooler periods and vice versa?

To identify marine heatwave events and estimate the severity of warming in a given year, the number of heatwave days per year was calculated for each site using the *detect()* function in the **heatwaveR** package [67]. The climatological mean for each site was calculated using bottom temperature data from August 2002 to July of 2025 [63]. Heatwave days were considered to be days when the average daily temperature exceeded the site-specific 90th percentile of the climatological mean for five or more consecutive days [68]. We estimated the degree of warming in the year preceding a benthic community survey, as the number of heatwave days in the calendar year beginning July 1st of the previous year through June 30th of the sampled year. The proportion of days in the year prior to sampling that were classified as heatwave days was calculated by dividing the number of heatwave days by the total number of days in the year (Fig. S1A).

To evaluate whether heatwave severity or duration were related to the degree to which community composition shifted during the strong heatwave years from 2015-2019 (see *Reef-Wide Shifts in Community Composition During Marine Heatwaves* below*),* we calculated the maximum annual number of heatwave days, the cumulative number of heatwave days from 2015-2019, and the cumulative intensity of heatwave conditions by site from 2015-2019. We also identified periods when bottom temperature exceeded the 90th percentile for a given site, excluding the climatological mean, using the *exceedance()* function. This estimate allowed us to assess whether the number of days that exceeded an absolute temperature threshold was an important indicator of thermal stress. For each site we calculated the total number of days per year that bottom temperature exceeded the 90th percentile and identified the year during the heatwave period (2015-2019) with the most days that exceeded this threshold.

Reef-Wide Shifts in Community Composition During Marine Heatwaves

Commented [A53]: How does the detect function work?

Commented [A54]: Good info to have earlier

We used linear mixed effects models and generalized linear mixed effects models in the **glmmTMB** package [69] to evaluate whether the proportion of heatwave days in the year prior to sampling was a significant predictor of total understory macroalgal biomass and species richness. The proportion of heatwave days per year and the biomass of the overstory, canopy forming giant kelp, *Macrocystis pyrifera,* were included in each model as fixed effects, as *Macrocystis* biomass has been shown to inhibit understory biomass and productivity at the study sites through its effect on light attenuation on the benthos [70–72]. A temporal autocorrelation term was included within site as a grouping variable to account for repeated measures.

We used *capscale*() in the **vegan** package [73] to perform a Canonical Analysis of Principal Coordinates (CAP) [74] to assess whether average community composition per site in July varied over time. The ordination was constrained by the interaction between site and time period: before the heatwave years (2002-2014), during the strong heatwave years (2015-2019), or after the heatwave years (2020-2025).

We performed a Permutational Multivariate Analysis of Variance (PERMANOVA) using the function *adonis2*() in the **vegan** package [73] to assess whether there were significant differences in species-based community composition as a function of time period. Prior to PERMANOVA, we evaluated whether groups exhibited homogeneity of group dispersions using the *betadisper*() function. Homogeneity of group dispersions between time periods did not differ significantly. Permutations were limited within each site as blocks.

We used the function *lm*() to evaluate whether linear correlations existed between heatwave severity from 2015-2019 and the shift in community composition by site from before the heatwave years (2002-2014) to after the heatwave years (2020-2025). We assessed multiple linear models with individual predictors estimating heatwave severity including the cumulative

Commented [A55]: Specify the factors

Commented [A56]: Unsure if a standard lm is appropriate given the response data and correlation of sites

and maximum annual heatwave intensity (estimated from the *detect*() function in **heatwaveR)**, the cumulative and maximum annual duration of heatwave conditions from 2015-2019, and the cumulative and maximum annual duration of days that exceeded the absolute 90th percentile temperature threshold for a given site. Average community composition shifts were estimated by calculating the distance in community centroids for each site and from before and after the heatwave years from the CAP (Fig. 2A).

To identify which species were significantly more abundant during a given time period, we used the **indicspecies** package [75] to perform an Indicator Species Analysis by time period across all sites.

Commented [A57]: How does that work?

Community Homogenization During and Following Marine Heatwaves

We used the function anosim() in the **vegan** package to perform multiple Analyses of Similarity (ANOSIM) to evaluate whether community composition between sites became more similar during or following heatwave events. Community similarity was evaluated across sites during three time periods: before the strong heatwave years (2002-2014), during the strong heatwave years (2015-2019), and after the strong heatwave years (2020-2025). We also evaluated whether sites that shifted considerably in composition after the heatwave years (Fig. 2C) became more similar to the warmest sites, Mohawk and Carpinteria.

Commented [A58]: Why not disper again?

Thermal Group Shifts During Marine Heatwaves

To assess whether warm-tolerant species have become more abundant over time or during years with many heatwave days, species were assigned to a thermal group corresponding to a historic range as defined by Abbott and Hollenberg [76] (Supplemental Table 2). Most species surveyed by the SBC LTER have a broad geographical distribution that extends from either

British Columbia or Alaska through Baja California, Mexico, and were classified as temperate. Species with ranges either extending beyond Baja California into central America or whose distribution was limited to the Santa Barbara Channel as the northernmost range limit were classified as subtropical. Non-native species to the Santa Barbara Channel were classified as introduced. Temperate species with a narrower thermal range bounded by southern Oregon and extending through Baja California were classified as transitional species. Biomass of species classified as northern-temperate where the Santa Barbara Channel is at the southern-most edge of their range were excluded from analyses as their biomass was negligible.

The changes in biomass of subtropical, introduced, transitional, and temperate species were evaluated in separate models against the proportion of heatwave days per year and the biomass of *Macrocystis pyrifera* using generalized linear mixed effects models. A temporal autocorrelation term was included within site to account for repeated measures for all models except for the model evaluating introduced species biomass. Inclusion of the temporal autocorrelation term prevented model convergence, and this term was excluded from the final model. To evaluate whether the change in biomass of subtropical species was significant over time, a separate model was fit with year as a predictor and site as a random effect.

For all linear and generalized linear mixed effects models presented, the **DHARMa** package [77] was used to evaluate model fit and residuals. All final models were accepted if the model Akaike Information Criterion (AIC) was significantly lower than that of the null model (unless specified in Supplementary Table 3) and there were no significant issues within the distribution of model residuals. Fixed effects were removed from the final model if they did not significantly improve model AIC. The best-fitting model for temperate species biomass was not significantly better than the null model, and the model with the lowest AIC value is presented.

The distributions of all response variables and model parameters for generalized and linear mixed effects models are included in Supplemental Table 3.

Susceptibility to Marine Heatwaves by Functional Group

We used generalized linear mixed effects models to evaluate whether the proportion of heatwave days in the year prior to sampling was a significant predictor of functional group biomass. Species were assigned to one of the following functional groups: understory kelps, non-kelp brown algae, green algae, GCA, and non-coralline red algae based on taxonomic relatedness (Supplemental Table 2). Initial models included the proportion of heatwave days in the year prior to sampling and *Macrocystis pyrifera* biomass (standardized to a mean of 0 and a standard deviation of 1) as fixed effects. A temporal autocorrelation term was included within site as a grouping variable to account for repeated measures.

There was a general positive trend in GCA biomass over time (Fig. 4A), and a separate model with year as a fixed effect and site as a random effect was fit to evaluate whether the biomass of GCA changed significantly over time.

References

1. Hoek, C. V. den. The distribution of benthic marine algae in relation to the temperature regulation of their life histories. *Biol. J. Linn. Soc.* **18**, 81–144 (1982).

2. Harley, C. D. G., Smith, K. F. & Moore, V. L. Environmental variability and biogeography: the relationship between bathymetric distribution and geographical range size in marine algae and gastropods. *Glob. Ecol. Biogeogr.* **12**, 499–506 (2003).

3. Tomanek, L. & Helmuth, B. Physiological ecology of rocky intertidal organisms: a synergy of concepts. *Integr. Comp. Biol.* **42**, 771–775 (2002).

4. Tittensor, D. P. *et al.* Global patterns and predictors of marine biodiversity across taxa. *Nature* **466**, 1098–1101 (2010).

5. Adey, W. H. & Steneck, R. S. Thermogeography over time creates biogeographic regions: a temperature/space/time-integrated model and an abundance-weighted test for benthic marine algae. *J. Phycol.* **37**, 677–698 (2001).

6. Cheng, L. *et al.* Past and future ocean warming. *Nat. Rev. Earth Environ.* **3**, 776–794 (2022).

7. Cheng, L. *et al.* Improved estimates of ocean heat content from 1960 to 2015. *Sci. Adv.* **3**, e1601545 (2017).

8. Poloczanska, E. S. *et al.* Global imprint of climate change on marine life. *Nat Clim Change* **3**, 919–925 (2013).

9. Burrows, M. T. *et al.* Ocean community warming responses explained by thermal affinities and temperature gradients. *Nat. Clim. Chang.* **9**, 959–963 (2019).

10. Pinsky, M. L., Selden, R. L. & Kitchel, Z. J. Climate-driven shifts in marine species ranges: scaling from organisms to communities. *Annu Rev Mar Sci* **12**, 1–27 (2019).

11. Smith, K. E. *et al.* Biological impacts of marine heatwaves. *Annu. Rev. Mar. Sci.* **15**, 119–145 (2022).

12. Reed, D. C. *et al.* Responses of coastal ecosystems to climate change: insights from long-term ecological research. *Bioscience* **72**, 871–888 (2022).

13. Holbrook, N. J. *et al.* A global assessment of marine heatwaves and their drivers. *Nat. Commun.* **10**, 2624 (2019).

14. Oliver, E. C. J. *et al.* Longer and more frequent marine heatwaves over the past century. *Nat Commun* **9**, 1324 (2018).

15. Frölicher, T. L., Fischer, E. M. & Gruber, N. Marine heatwaves under global warming. *Nature* **560**, 360–364 (2018).

16. Smith, K. E. *et al.* Socioeconomic impacts of marine heatwaves: Global issues and opportunities. *Science* **374**, eabj3593 (2021).

17. Reed, D. C. *et al.* Responses of coastal ecosystems to climate change: insights from long-term ecological research. *Bioscience* **72**, 871–888 (2022).

18. Smale, D. A. *et al.* Marine heatwaves threaten global biodiversity and the provision of ecosystem services. *Nat Clim Change* **9**, 306–312 (2019).

19. Sunday, J. M., Bates, A. E. & Dulvy, N. K. Thermal tolerance and the global redistribution of animals. *Nat Clim Change* **2**, 686–690 (2012).

20. Cheung, W. W. L. *et al.* Projecting global marine biodiversity impacts under climate change scenarios. *Fish Fish* **10**, 235–251 (2009).

21. Morley, J. W. *et al.* Projecting shifts in thermal habitat for 686 species on the North American continental shelf. *PLoS ONE* **13**, e0196127 (2018).

22. Félix-Loaiza, A. C. *et al.* Marine heatwaves facilitate invasive algae takeover as foundational kelp. *Bot Mar* **65**, 315–319 (2022).

23. Montie, S. & Thomsen, M. S. Long-term community shifts driven by local extinction of an iconic foundation species following an extreme marine heatwave. *Ecol. Evol.* **13**, e10235 (2023).

24. Free, C. M. *et al.* Impact of the 2014–2016 marine heatwave on US and Canada West Coast fisheries: Surprises and lessons from key case studies. *Fish Fish.* **24**, 652–674 (2023).

25. Villaseñor-Derbez, J. C., Arafeh-Dalmau, N. & Micheli, F. Past and future impacts of marine heatwaves on small-scale fisheries in Baja California, Mexico. *Commun. Earth Environ.* **5**, 623 (2024).

26. Richardson, L. E., Graham, N. A. J., Pratchett, M. S., Eurich, J. G. & Hoey, A. S. Mass coral bleaching causes biotic homogenization of reef fish assemblages. *Glob. Chang. Biol.* **24**, 3117–3129 (2018).

27. Harley, C. D. G. Climate change, keystone predation, and biodiversity loss. *Science* **334**, 1124–1127 (2011).

28. Teagle, H., Hawkins, S. J., Moore, P. J. & Smale, D. A. The role of kelp species as biogenic habitat formers in coastal marine ecosystems. *J Exp Mar Biol Ecol* **492**, 81–98 (2017).

29. Graham, M. H. Effects of local deforestation on the diversity and structure of Southern California giant kelp forest food webs. *Ecosystems* **7**, 341–357 (2004).

30. Ronnback, P. *et al.* Ecosystem goods and services from Swedish coastal habitats: identification, valuation, and implications of ecosystem shifts. *AMBIO: A J. Hum. Environ.* **36**, 534–544 (2007).

31. Straub, S. C. *et al.* Resistance, extinction, and everything in between – the diverse responses of seaweeds to marine heatwaves. *Front. Mar. Sci.* **6**, 763 (2019).

32. Smale, D. A. & Wernberg, T. Extreme climatic event drives range contraction of a habitat-forming species. *Proc. R. Soc. B: Biol. Sci.* **280**, 20122829 (2013).

33. Wernberg, T. *et al.* Climate-driven regime shift of a temperate marine ecosystem. *Science* **353**, 169–172 (2016).

34. Cavanaugh, K. C., Reed, D. C., Bell, T. W., Castorani, M. C. N. & Beas-Luna, R. Spatial variability in the resistance and resilience of giant kelp in Southern and Baja California to a multiyear heatwave. *Frontiers Mar Sci* **6**, 413 (2019).

35. Zaba, K. D. & Rudnick, D. L. The 2014–2015 warming anomaly in the Southern California Current System observed by underwater gliders. *Geophys. Res. Lett.* **43**, 1241–1248 (2016).

36. Wei, X., Li, K., Kilpatrick, T., Wang, M. & Xie, S. Large-scale conditions for the record-setting Southern California marine heatwave of August 2018. *Geophys. Res. Lett.* **48**, (2021).

37. Reed, D. *et al.* Extreme warming challenges sentinel status of kelp forests as indicators of climate change. *Nat Commun* **7**, 13757 (2016).

38. Lorenzo, E. D. & Mantua, N. Multi-year persistence of the 2014/15 North Pacific marine heatwave. *Nat Clim Change* **6**, 1042–1047 (2016).

39. Rendina, F. *et al.* Physiological response of the coralline alga *Corallina officinalis* L. to both predicted long-term increases in temperature and short-term heatwave events. *Mar. Environ. Res.* **150**, 104764 (2019).

40. Lamy, T. *et al.* Species insurance trumps spatial insurance in stabilizing biomass of a marine macroalgal metacommunity. *Ecology* **100**, e02719 (2019).

41. Thomsen, M. S. *et al.* Local extinction of bull kelp (*Durvillaea* spp.) due to a marine heatwave. *Front. Mar. Sci.* **6**, 84 (2019).

42. Rogers-Bennett, L. & Catton, C. A. Marine heat wave and multiple stressors tip bull kelp forest to sea urchin barrens. *Sci. Rep.* **9**, 15050 (2019).

43. Amaya, D. J., Miller, A. J., Xie, S.-P. & Kosaka, Y. Physical drivers of the summer 2019 North Pacific marine heatwave. *Nat. Commun.* **11**, 1903 (2020).

44. Winant, C. D., Dever, E. P. & Hendershott, M. C. Characteristic patterns of shelf circulation at the boundary between central and southern California. *J. Geophys. Res.: Oceans* **108**, (2003).

45. Michaud, K. M., Reed, D. C. & Miller, R. J. The Blob marine heatwave transforms California kelp forest ecosystems. *Commun Biology* **5**, 1143 (2022).

46. Matson, P. G. & Edwards, M. S. Effects of ocean temperature on the southern range limits of two understory kelps, *Pterygophora californica* and *Eisenia arborea*, at multiple life-stages. *Mar. Biol.* **151**, 1941–1949 (2007).

47. Muth, A. F., Graham, M. H., Lane, C. E. & Harley, C. D. G. Recruitment tolerance to increased temperature present across multiple kelp clades. *Ecology* **100**, e02594 (2019).

48. Kim, J.-H., Min, J., Kang, E. J. & Kim, K. Y. Elevated temperature and changed carbonate chemistry: effects on calcification, photosynthesis, and growth of *Corallina officinalis* (Corallinales, Rhodophyta). *Phycologia* **57**, 280–286 (2017).

49. Cornwall, C. E., Diaz-Pulido, G. & Comeau, S. Impacts of ocean warming on coralline algal calcification: meta-analysis, knowledge gaps, and key recommendations for future research. *Front. Mar. Sci.* **6**, 186 (2019).

50. Carballo, J. L., Olabarria, C. & Osuna, T. G. Analysis of four macroalgal assemblages along the Pacific Mexican Coast during and after the 1997–98 El Niño. *Ecosystems* **5**, 0749–0760 (2002).

51. Lopez-Perez, A. *et al.* Reef community changes associated with the 2009-2010 El Niño in the Southern Mexican Pacific. *Pac. Sci.* **70**, 175–190 (2016).

52. Gunnill, F. C. Population fluctuations of seven macroalgae in Southern California during 1981–1983 including effects of severe storms and an El Niño. *J. Exp. Mar. Biol. Ecol.* **85**, 149–164 (1985).

53. Atkinson, J., King, N. G., Wilmes, S. B. & Moore, P. J. Summer and winter marine heatwaves favor an invasive over native seaweeds. *J Phycol* **56**, 1591–1600 (2020).

54. Tanaka, K., Taino, S., Haraguchi, H., Prendergast, G. & Hiraoka, M. Warming off southwestern Japan linked to distributional shifts of subtidal canopy-forming seaweeds. *Ecol. Evol.* **2**, 2854–2865 (2012).

55. Bekkby, T., Angeltveit, G., Gundersen, H., Tveiten, L. & Norderhaug, K. M. Red sea urchins (*Echinus esculentus*) and water flow influence epiphytic macroalgae density. *Mar. Biol. Res.* **11**, 375–384 (2015).

56. Lamy, T. *et al.* Scale-specific drivers of kelp forest communities. *Oecologia* **186**, 217–233 (2018).

57. Toohey, B. D. The relationship between physical variables on topographically simple and complex reefs and algal assemblage structure beneath an *Ecklonia radiata* canopy. *Estuar., Coast. Shelf Sci.* **71**, 232–240 (2007).

58. Simons, R. D. & Catlett, D. Regulation of a surface chlorophyll hotspot by wind-driven upwelling and eddy circulation in the Santa Barbara Channel, Southern California. *Prog. Oceanogr.* **217**, 103096 (2023).

59. Smale, D. A., Epstein, G., Hughes, E., Mogg, A. O. M. & Moore, P. J. Patterns and drivers of understory macroalgal assemblage structure within subtidal kelp forests. *Biodivers Conserv* **29**, 4173–4192 (2020).

60. Wernberg, T., Thomsen, M. S., Tuya, F. & Kendrick, G. A. Biogenic habitat structure of seaweeds change along a latitudinal gradient in ocean temperature. *J. Exp. Mar. Biol. Ecol.* **400**, 264–271 (2011).

61. Horn, M. H. & Allen, L. G. A distributional analysis of California coastal marine fishes. *J. Biogeogr.* **5**, 23 (1978).

62. Reed, D. C. & Miller, R. J. SBC LTER: Reef: Annual time series of biomass for kelp forest species, ongoing since 2000 ver 18. *Environmental Data Initiative* (2025). https://doi.org/10.6073/pasta/cb45bf15430ba570828444b13dd8521f.

63. Reed, D. C. & Miller, R. J. SBC LTER: Reef: Bottom Temperature: Continuous water temperature, ongoing since 2000 ver 33. *Environmental Data Initiative* (*In Prep*).

64. Rassweiler, A., Reed, D. C., Harrer, S. L. & Nelson, J. C. Improved estimates of net primary production, growth, and standing crop of *Macrocystis pyrifera* in Southern California. *Ecology* **99**, 2132–2132 (2018).

65. Reed, D. C., Nelson, J. C., Harrer, S. L. & Miller, R. J. Estimating biomass of benthic kelp forest invertebrates from body size and percent cover data. *Mar Biol* **163**, 101 (2016).

66. Nelson, C. J., Reed, D. C., Harrer, S. & Miller, R. J. SBC LTER: Reef: Coefficients for estimating biomass from body size or percent cover for kelp forest species ver 3. *Environmental Data Initiative* (2021).

67. Schlegel, R. W. & Smit, A. J. heatwaveR: A central algorithm for the detection of heatwaves and cold-spells. *Journal of Open Source Software* **3**, 821 (2018).

68. Hobday, A. J. *et al.* A hierarchical approach to defining marine heatwaves. *Prog Oceanogr* **141**, 227–238 (2016).

69. Brooks, M. E. *et al.* glmmTMB balances speed and flexibility among packages for zero-inflated generalized linear mixed modeling. *The R Journal* **9**, 378–400 (2017).

70. Castorani, M. C. N., Reed, D. C. & Miller, R. J. Loss of foundation species: disturbance frequency outweighs severity in structuring kelp forest communities. *Ecology* **99**, 2442–2454 (2018).

71. Detmer, A. R. *et al.* Variation in disturbance to a foundation species structures the dynamics of a benthic reef community. *Ecology* **102**, e03304 (2021).

72. Castorani, M. C. N., Harrer, S. L., Miller, R. J. & Reed, D. C. Disturbance structures canopy and understory productivity along an environmental gradient. *Ecol. Lett.* **24**, 2192–2206 (2021).

73. Oksanen, J. *et al. Vegan: Community Ecology Package*. (2020).

74. Anderson, M. J. & Willis, T. J. Canonical analysis of principal coordinates: a useful method of constrained ordination for ecology. *Ecology* **84**, 511–525 (2003).

75. Cáceres, M. D. & Jansen, F. *Indicspecies: Relationship between Species and Groups of Sites*. (2020).

76. Abbott, I. A. & Hollenberg, G. J. *Marine Algae of California*. (Stanford University Press, Stanford, CA, 1992).

77. Hartig, F. *DHARMa: Residual Diagnostics for Hierarchical (Multi-Level / Mixed) Regression Models.* (2022).
