# Supplementary material for: Macroalgal community transformation during successive marine heatwaves in southern California kelp forests
Source: Commun Earth Environ. 2026 May 16;7(1):632. doi: 10.1038/s43247-026-03599-5 (PMC13437228; doi:10.1038/s43247-026-03599-5)
Supplement: Supplementary file 2 [file 43247_2026_3599_MOESM2_ESM.pdf]

Macroalgal community transformation during successive marine heatwaves in southern California kelp forests

Kristen M. Michaud*, Daniel C. Reed, and Robert J. Miller

Marine Science Institute, University of California Santa Barbara, Santa Barbara, CA 93106

*Corresponding author: kmmichaud@ucsb.edu

Supplementary Information

Supplementary Figure 1

Supplementary Figure 2

Supplementary Figure 3

Supplementary Figure 4

Supplementary Figure 5

Supplementary Figure 6

Supplementary Table 1

Supplementary Figure 7

Supplementary Figure 8

Supplementary Figure 9

Supplementary Figure 10

Supplementary Figure 11

Supplementary Figure 12

Supplementary Figure 13

Supplementary Figure 14

Supplementary Figure 15

Supplementary Figure 16

Supplementary Figure 17

Supplementary Figure 18

Supplementary Figure 19

Supplementary Figure 20

Supplementary Figure 21

Supplementary Figure 22

Supplementary Figure 23

Supplementary Note 1

Supplementary Table 2

Supplementary Table 3

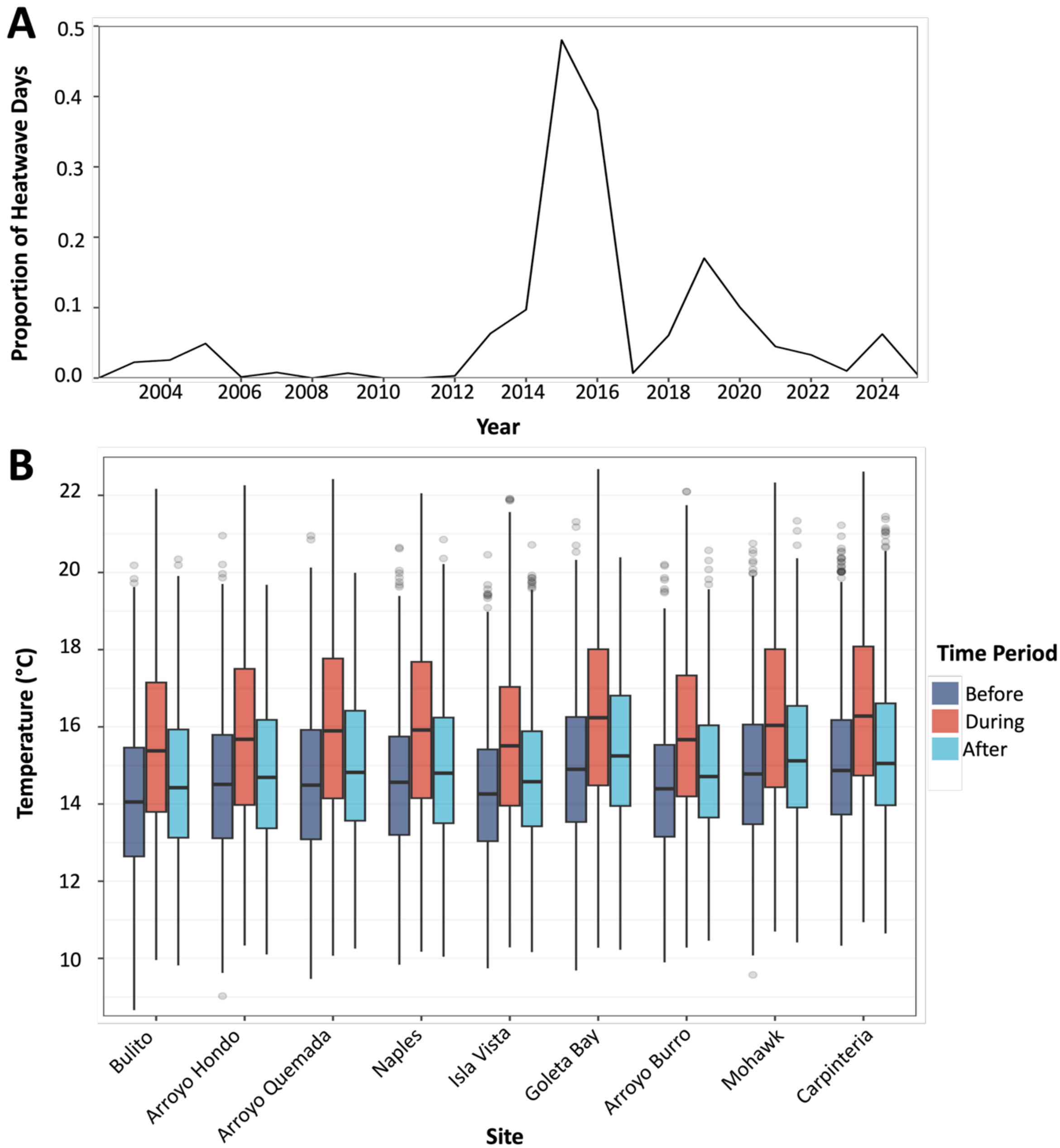

Supplementary Figure 1. A) Mean proportion of heatwave days averaged across the 9 sites by year. B) Daily bottom temperature across sites before (Aug 2002 - June 2014), during (July 2014 – June 2019), and after (July 2019 – June 2025) the strong heatwave years. Sites are arranged from west to east.

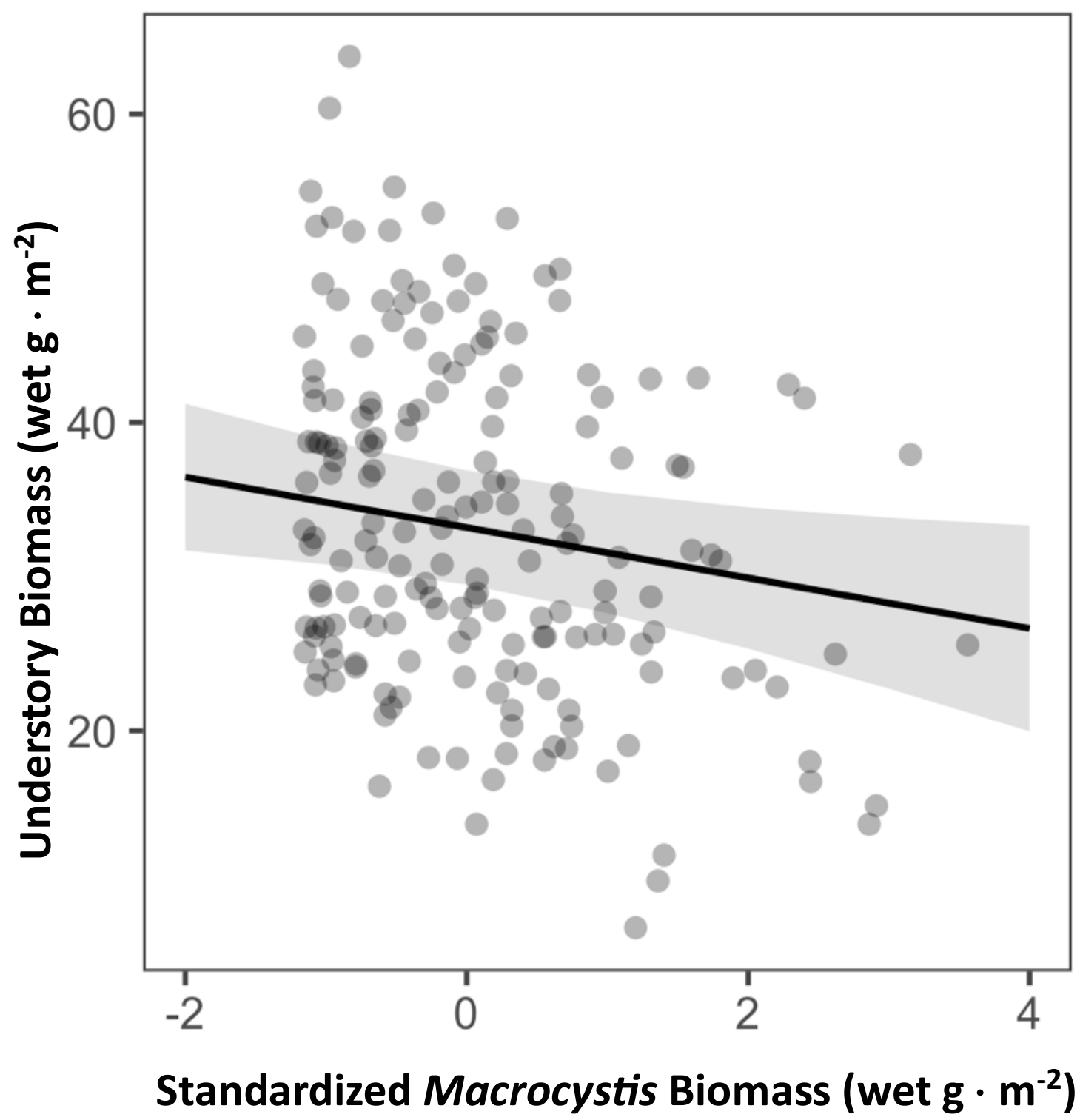

Supplementary Figure 2. Results of Model 1. Total understory biomass (mean wet g/m^2 by site) as a function of *Macrocystis pyrifera* biomass (mean wet g · m^{-2} by site) which has been standardized to a mean of 0 and standard deviation of 1. The solid line represents the results of linear mixed effects modeling while the grey shading indicates the 95% confidence interval. Understory biomass has been square root transformed.

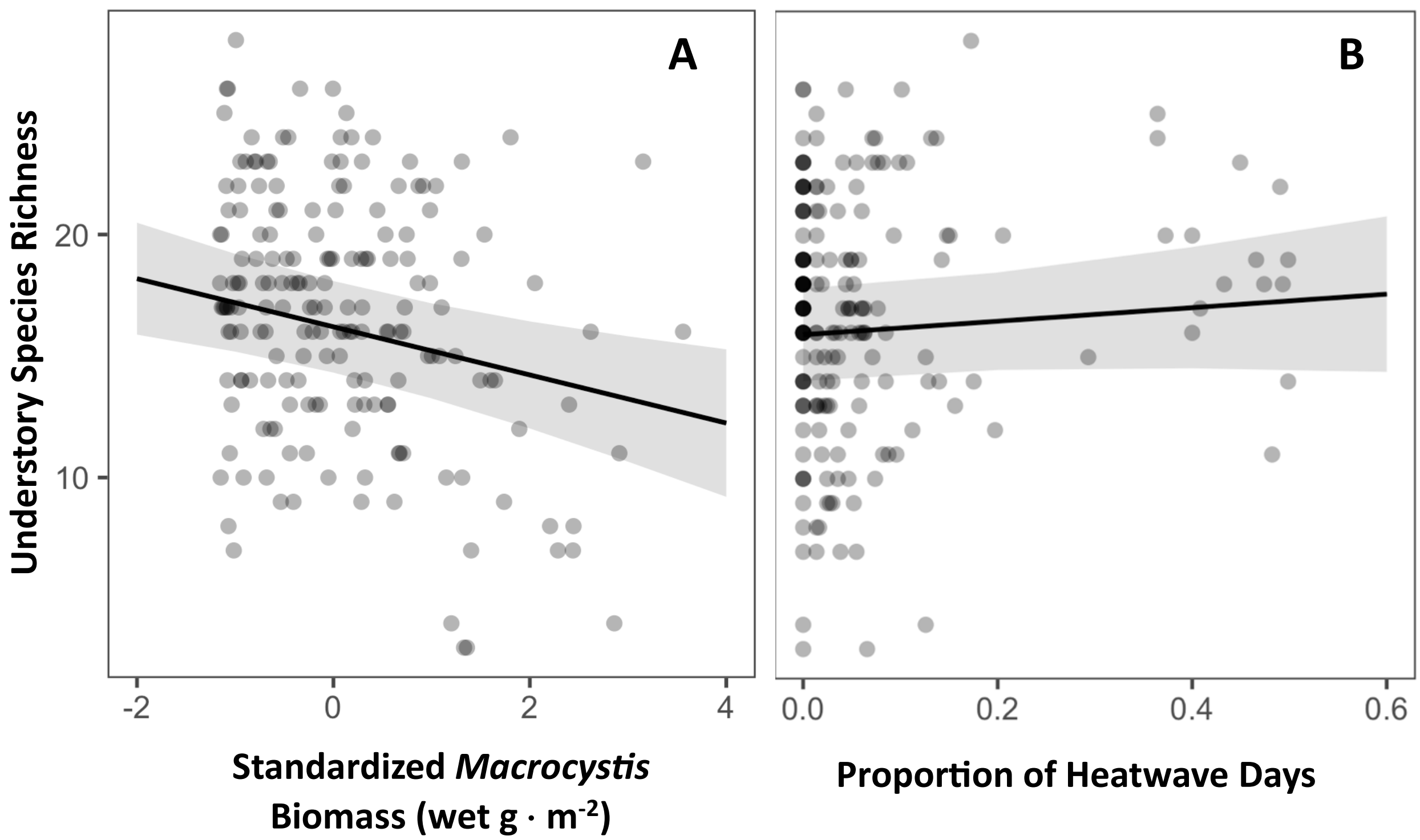

Supplementary Figure 3. Results of Model 2. Understory species richness (mean number of species per site) as a function of A) *Macrocystis pyrifera* biomass (mean wet g · m^{-2} by site) which has been standardized to a mean of 0 and standard deviation of 1 and B) the proportion of heatwave days. The solid line represents the results of linear mixed effects modeling while the grey shading indicates the 95% confidence interval.

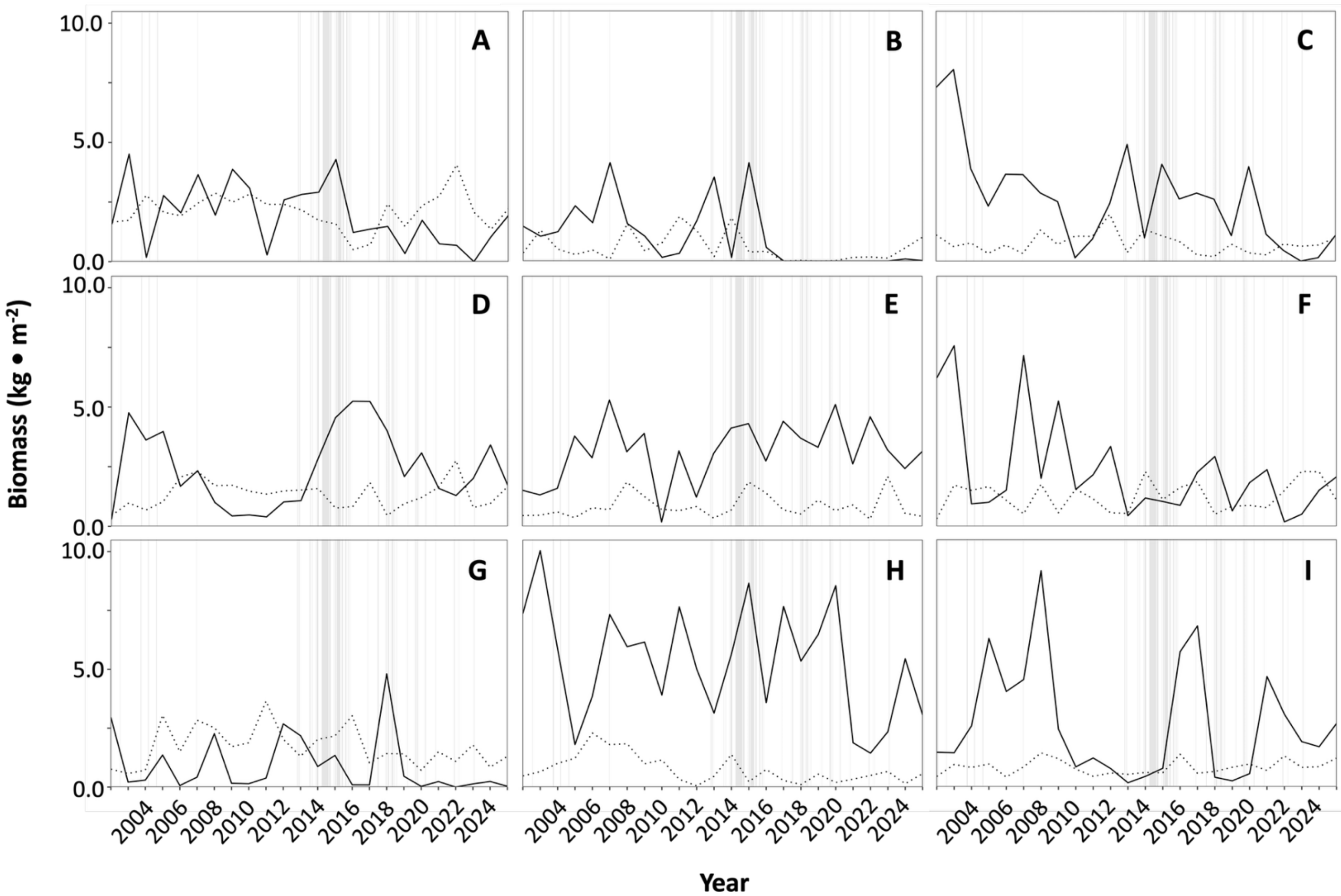

Supplementary Figure 4. Biomass of *Macrocystis pyrifera* (wet kg/m^2) in the solid black line and total understory biomass in the dotted line from west to east at A) Bulito, B) Arroyo Hondo, C) Arroyo Quemada, D) Naples, E) Isla Vista, F) Goleta Bay, G) Arroyo Burro, H) Mohawk, and I) Carpinteria reefs. Heatwave periods are represented with gray shading. Tick marks on the x-axis signify July (the month in which sampling occurred).

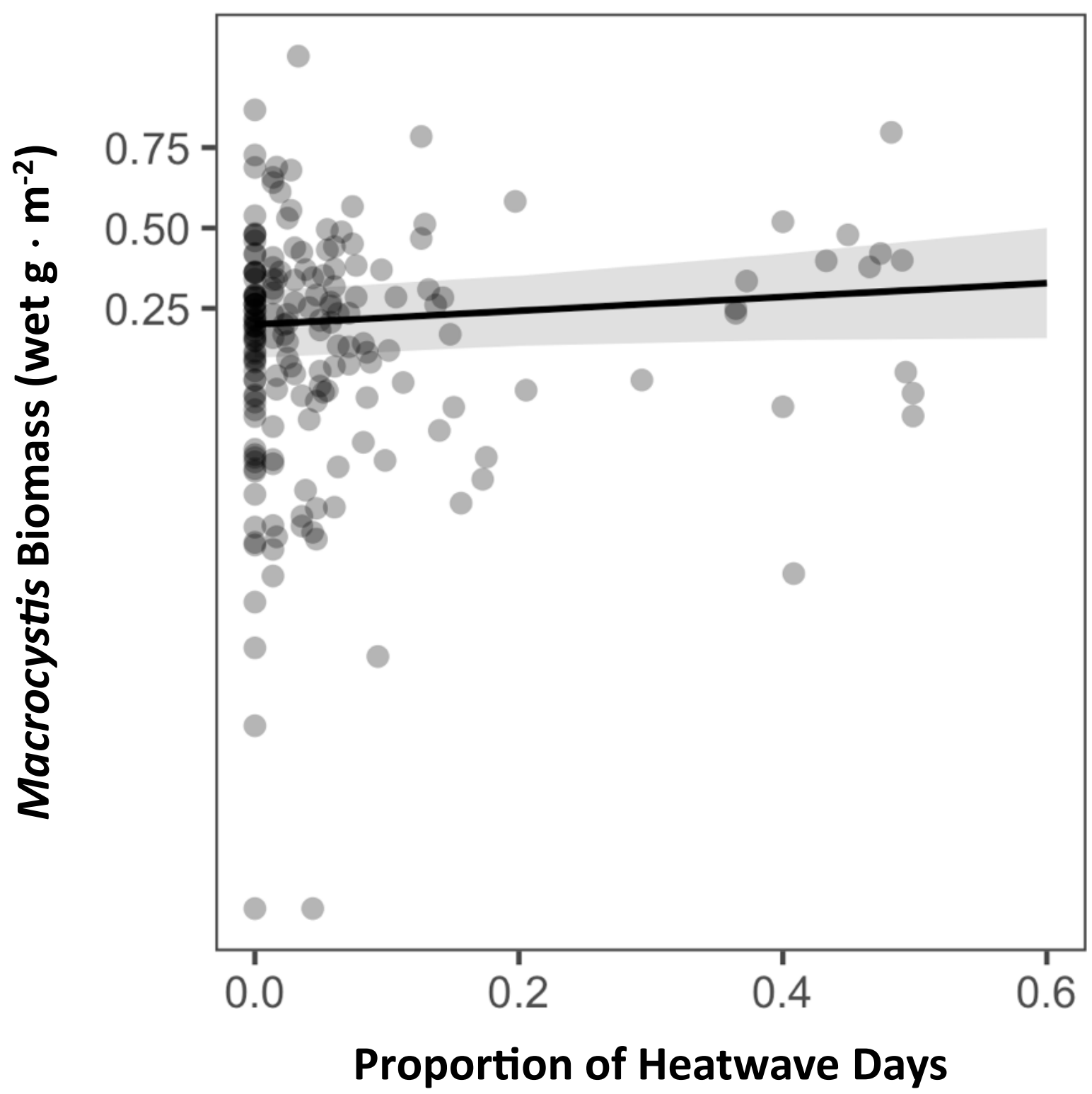

Supplementary Figure 5. Results of Model 3. *Macrocystis* biomass (mean wet g · m^{-2} by site) as a function of the proportion of heatwave days per year. The solid line represents the results of generalized linear mixed effects modeling while the grey shading indicates the 95% confidence interval. *Macrocystis* biomass has been transformed to fall between 0 and 1 in compliance with the beta distribution and is represented along the logit scale.

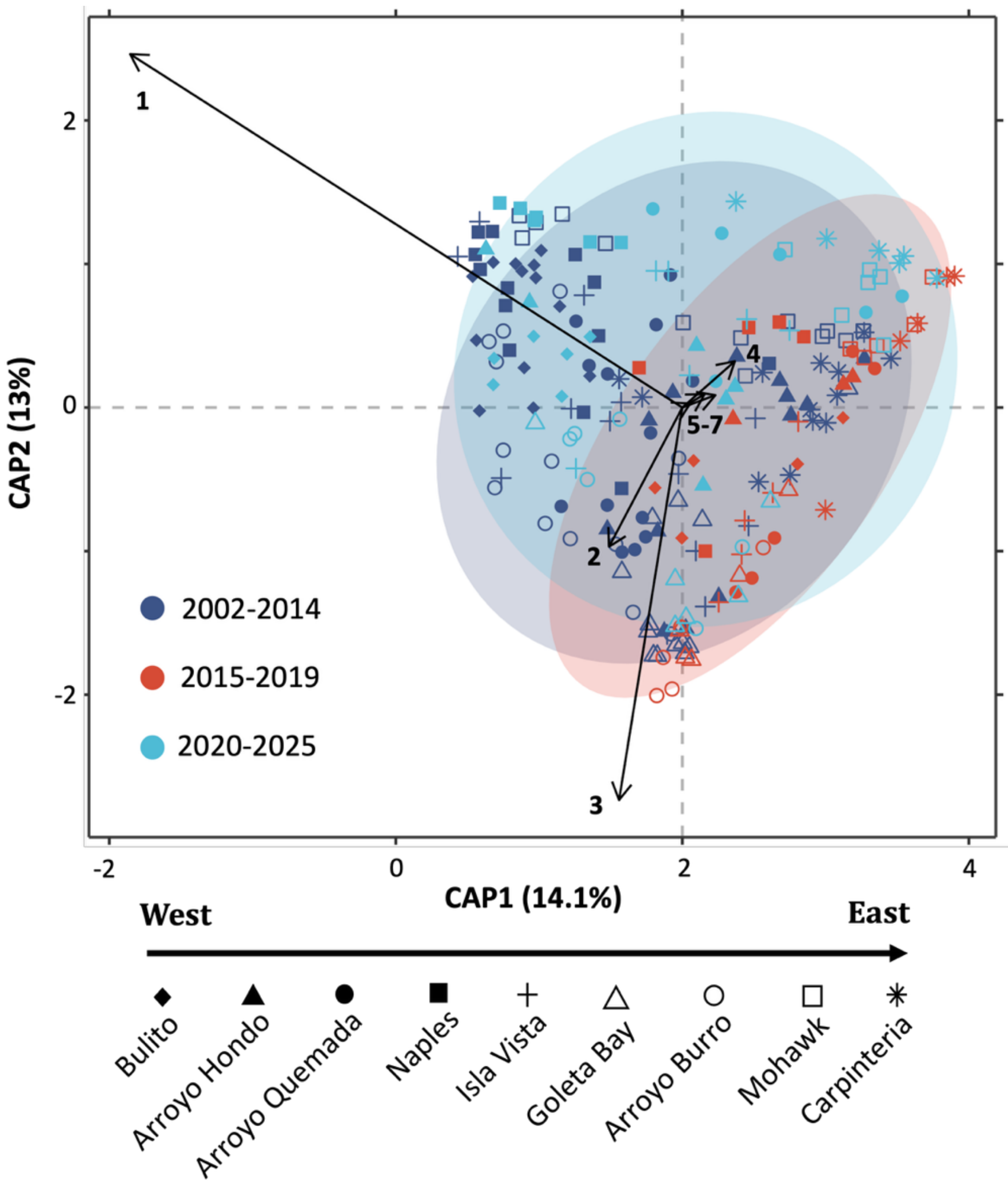

Supplementary Figure 6. Canonical Analysis of Principal Coordinates for community composition across the 9 sites and three time periods (R^2=0.488). The ordination was constrained by the interaction between site and time period (F=5.916, p<0.001) and points represent community composition at each site during each year. The ellipses represent the spread of all points during each time period across all sites and years. Symbols correspond to the nine sites. Numbered arrows correspond to the species that contributed the most to community dissimilarity over time with r>0.20: 1) *Pterygophora californica* (understory kelp), 2) *Gracilaria* spp. (non-coralline red alga) 3) *Desmartestia ligulata* (non-kelp brown alga), 4) *Corallina officinalis* (geniculate coralline alga), 5) *Dictyota* spp. (non-kelp brown alga), 6) *Gelidium* spp. (non-coralline red alga) and 7) *Dictyopteris undulata* (non-kelp brown alga).

Supplementary Table 1. Model results for all linear models with centroid distance as a response variable. * signifies p-values between 0.05 and 0.10. The two strongest predictors are bolded.

**Predictor**	***F***	**DF**	R^2	β	**95% CI**	***SE***	***t***	***p***
(Intercept)<br>Cumulative MHW Intensity	1.288	7	0.155	-1.961<br>0.003	[-7.722, 3.801]<br>[-0.003, 0.008]	2.436<br>0.002	-0.805<br>1.135	0.447<br>0.294
(Intercept)<br>Cumulative 2014-15 MHW Intensity	0.125	7	0.018	-0.016<br>0.001	[-5.482, 5.449]<br>[-0.005, 0.007]	2.311<br>0.003	-0.007<br>0.354	0.995<br>0.734
(Intercept)<br>**Cumulative 2018 MHW Intensity**	5.460	7	0.438	-1.187<br>0.012	[-3.215, 0.840]<br>[-0.0001, 0.025]	0.858<br>0.005	-1.385<br>2.337	0.209<br>0.052 *
(Intercept)<br>Cumulative MHW Duration	0.902	7	0.116	-1.842<br>0.007	[-8.364, 4.679]<br>[-0.010, 0.023]	2.758<br>0.007	-0.668<br>0.959	0.526<br>0.369
(Intercept)<br>Max Annual MHW Duration	0.058	7	0.008	-0.053<br>0.005	[-8.437, 8.331]<br>[-0.043, 0.053]	3.546<br>0.020	-0.015<br>0.241	0.988<br>0.817
(Intercept)<br>Cumulative Threshold Exceedance	0.281	7	0.039	-0.272<br>0.003	[-5.062, 4.519]<br>[-0.010, 0.016]	2.026<br>0.006	-0.134<br>0.530	0.897<br>0.612
(Intercept)<br>**Max Annual Threshold Exceedance**	4.746	7	0.404	-3.143<br>0.035	[-7.432, 1.145]<br>[-0.003, 0.073]	1.814<br>0.016	-1.733<br>2.178	0.127<br>0.066 *

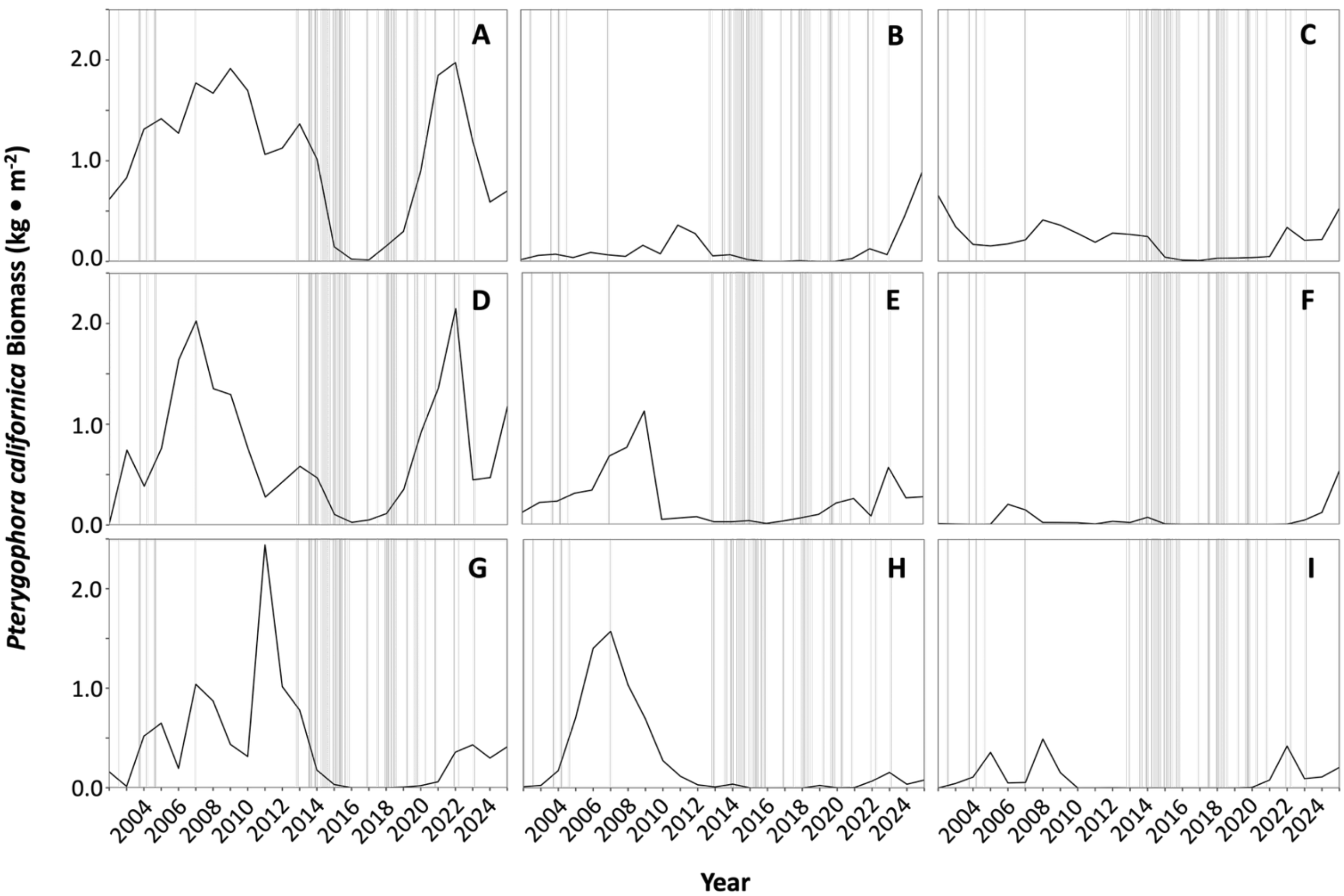

Supplementary Figure 7. Biomass (wet kg · m^{-2}) of *Pterygophora californica* across the thermal gradient from west to east at A) Bulito, B) Arroyo Hondo, C) Arroyo Quemada, D) Naples, E) Isla Vista, F) Goleta Bay, G) Arroyo Burro, H) Mohawk, and I) Carpinteria reefs. Heatwave periods are represented with gray shading. Tick marks on the x-axis signify July (the month in which sampling occurred).

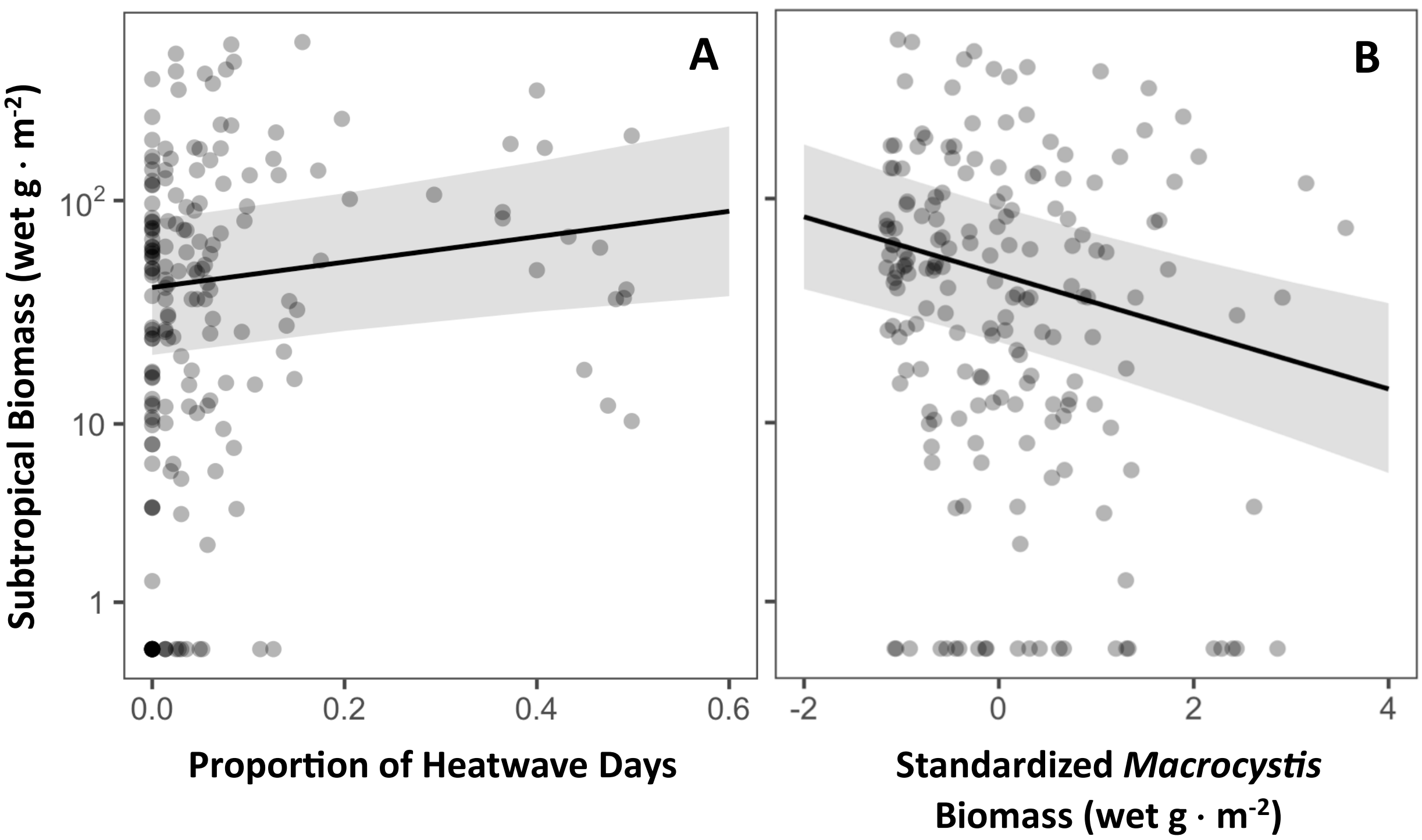

Supplementary Figure 8. Results of Model 4. Subtropical species biomass (mean wet g · m^{-2} by site) as a function of A) the proportion of heatwave days per year by site and B) *Macrocystis pyrifera* biomass (wet g · m^{-2}) which has been standardized to a mean of 0 and standard deviation of 1. The solid line represents the results of generalized linear mixed effects modeling while the grey shading indicates the 95% confidence interval. Subtropical species biomass is represented on a log scale.

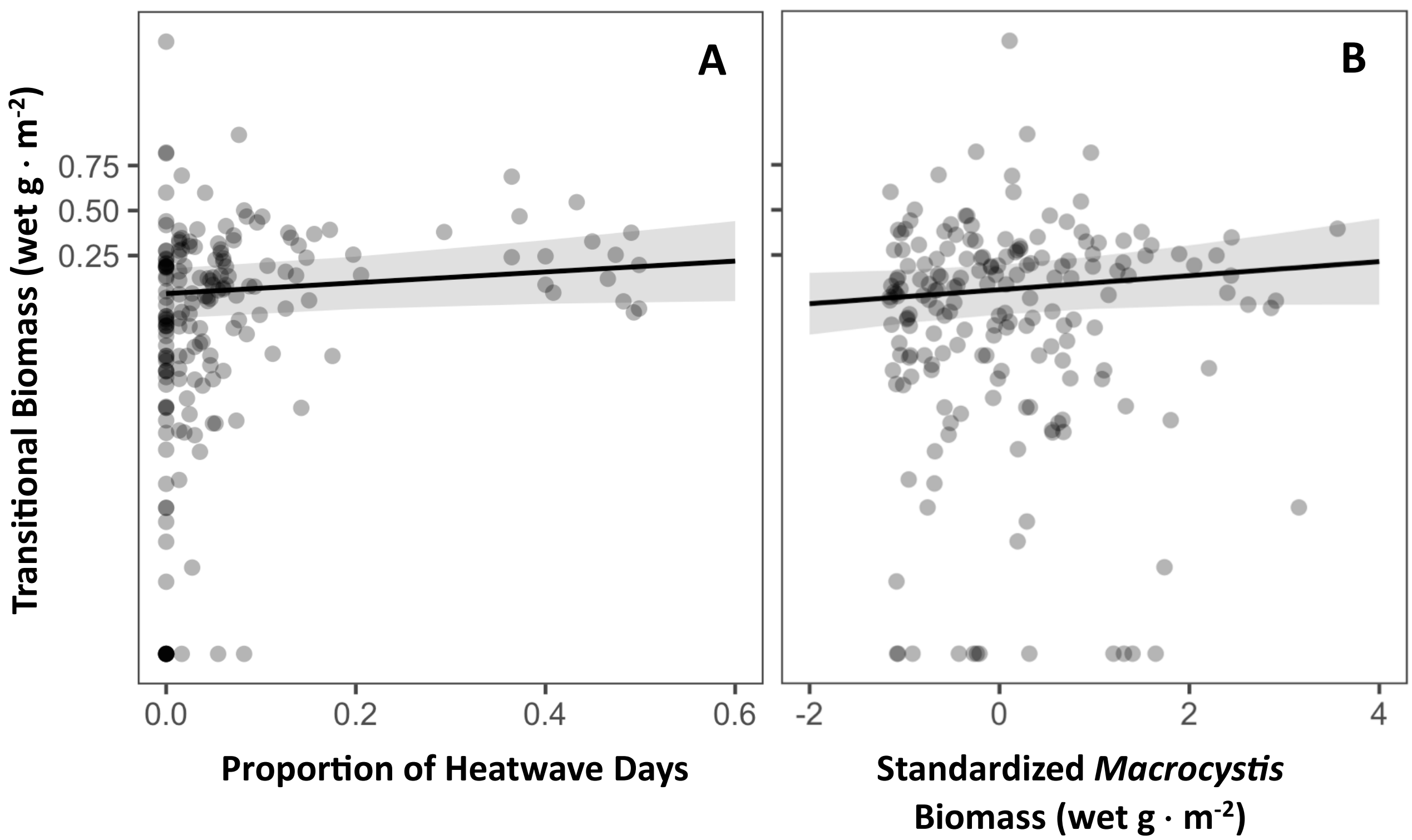

Supplementary Figure 9. Results of Model 6. Transitional species biomass (mean wet g · m^{-2} by site) as a function of A) the proportion of heatwave days per year and B) *Macrocystis pyrifera* biomass (wet g · m^{-2}) which has been standardized to a mean of 0 and standard deviation of 1. The solid line represents the results of generalized linear mixed effects modeling while the grey shading indicates the 95% confidence interval. Transitional species biomass has been transformed to fall between 0 and 1 in compliance with the beta distribution and is represented on the logit scale.

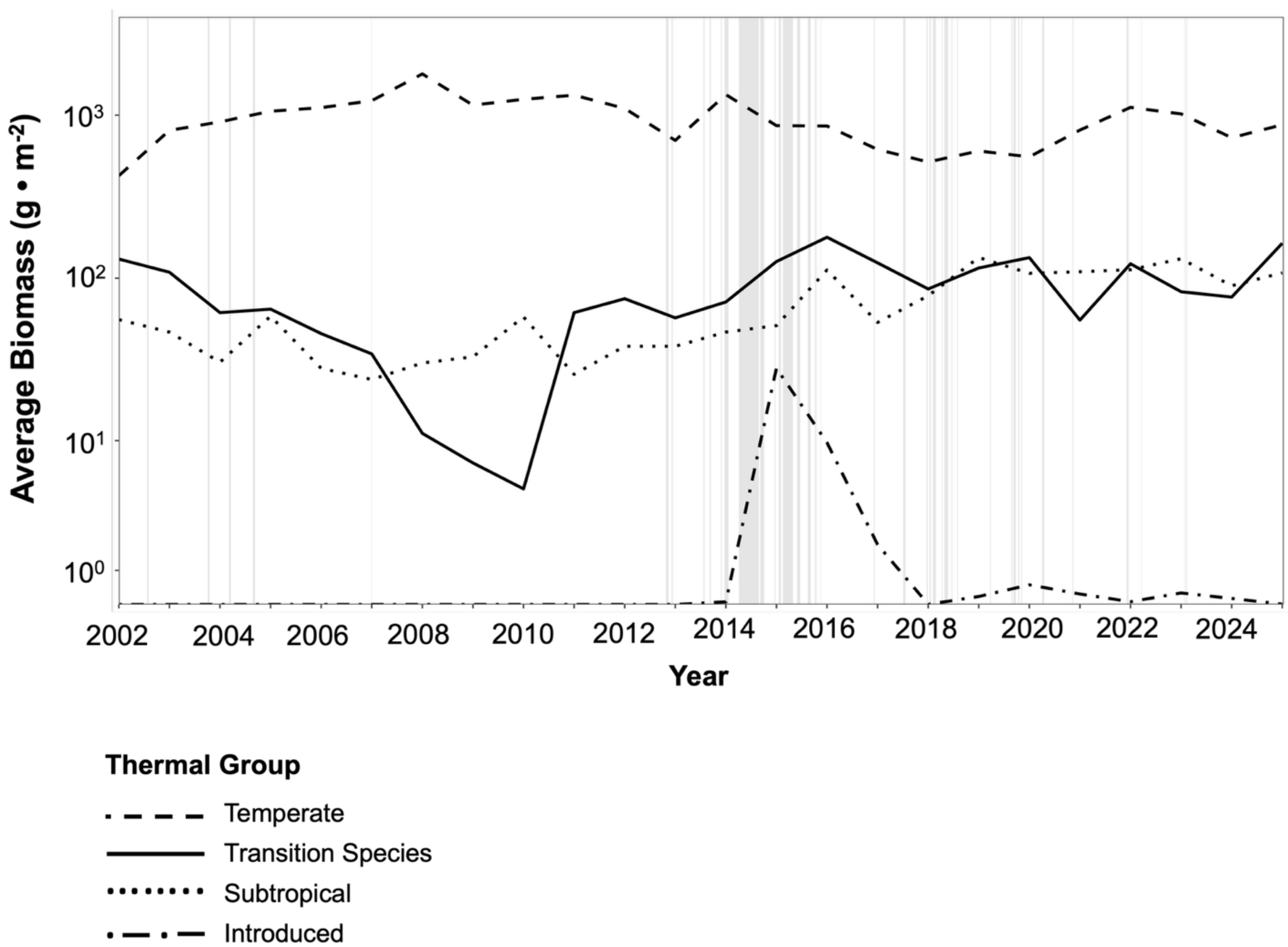

Supplementary Figure 10. Mean annual biomass of understory macroalgae averaged across sites by thermal group. Biomass (wet g · m^{-2}) is represented on a log scale. Error bars represent standard error. Heatwave days are shown in grey shading. Tick marks on the x-axis signify July (the month in which sampling occurred).

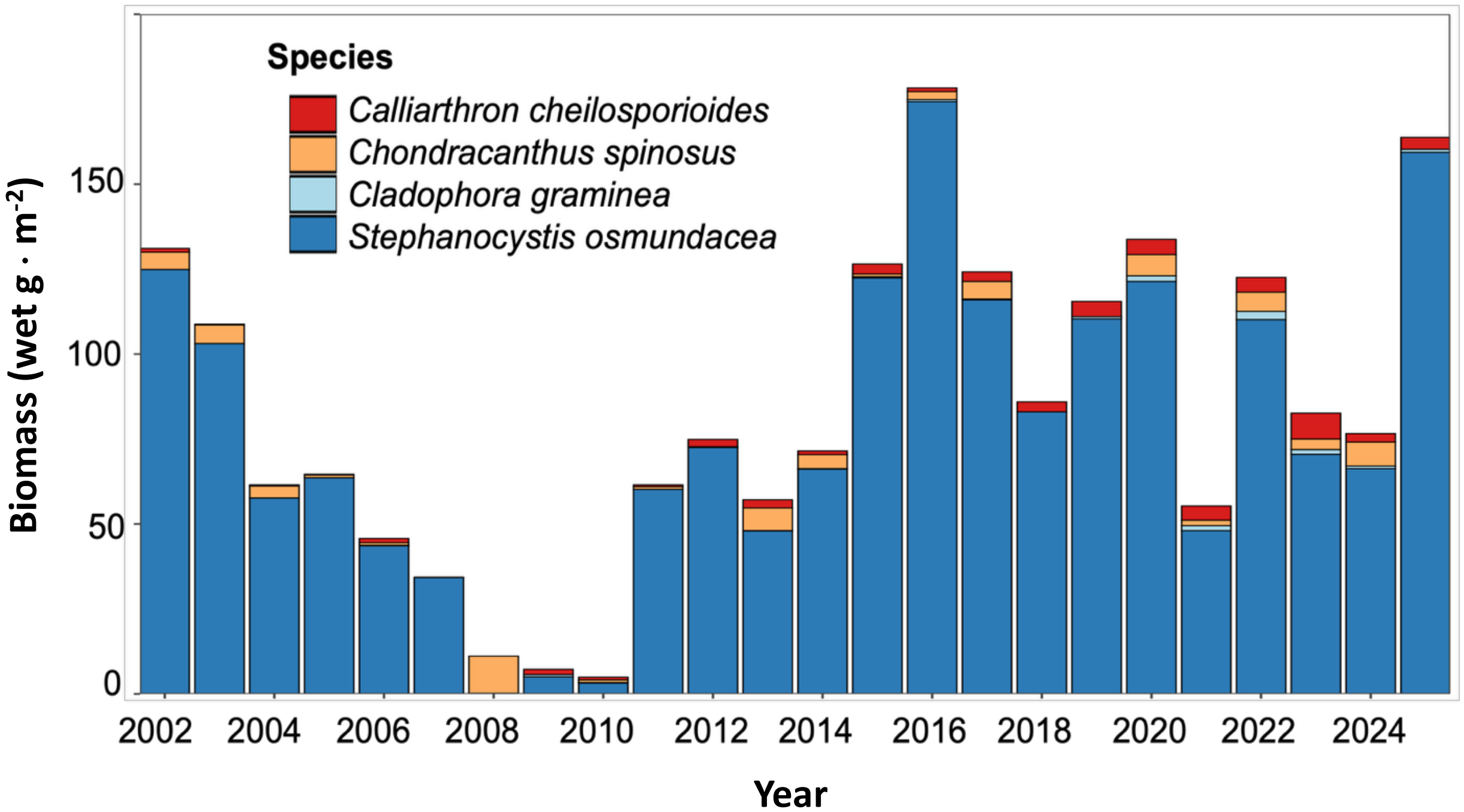

Supplementary Figure 11. Mean biomass (wet g · m^{-2}) of transitional species averaged across the 9 sites. *Calliarthron cheilosporioides* is a geniculate coralline alga, *Chondracanthus spinosus* is a non-coralline red alga, *Cladophora graminea* is a green alga, and *Stephanocystis osmundacea* is a non-kelp brown alga.

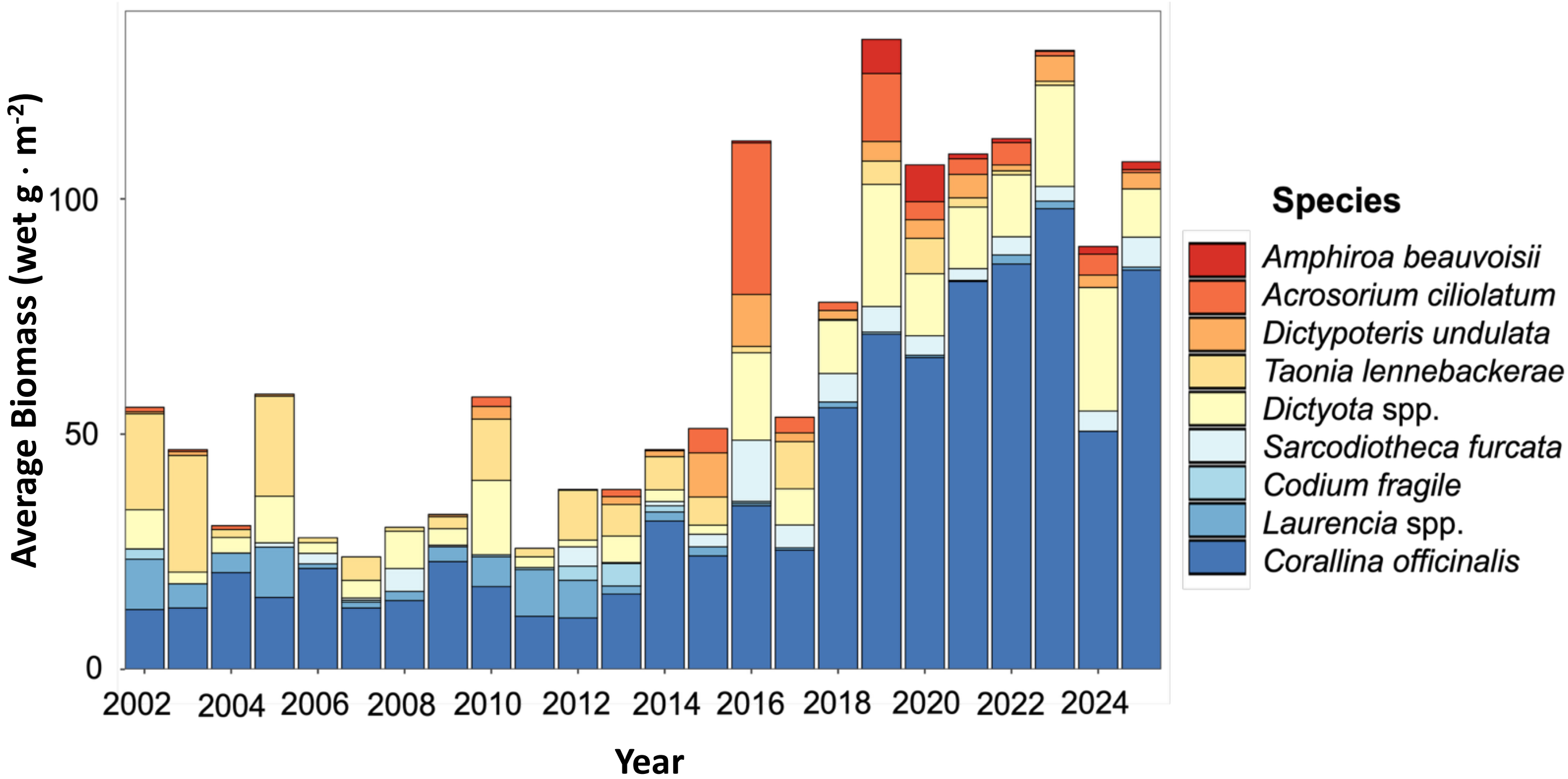

Supplementary Figure 12. Mean biomass (wet g · m^{-2}) of subtropical species averaged across the 9 sites. *Amphiroa beauvoisii* and *Corallina officinalis* are geniculate coralline algae, *Acrosorium ciliolatum, Sarcodiotheca furcata,* and *Laurencia* spp. are non-coralline red algae, *Dictyopteris undulata, Taonia lennebackerae,* and *Dictyota* spp. are non-kelp brown algae, and *Codium fragile* is a green alga.

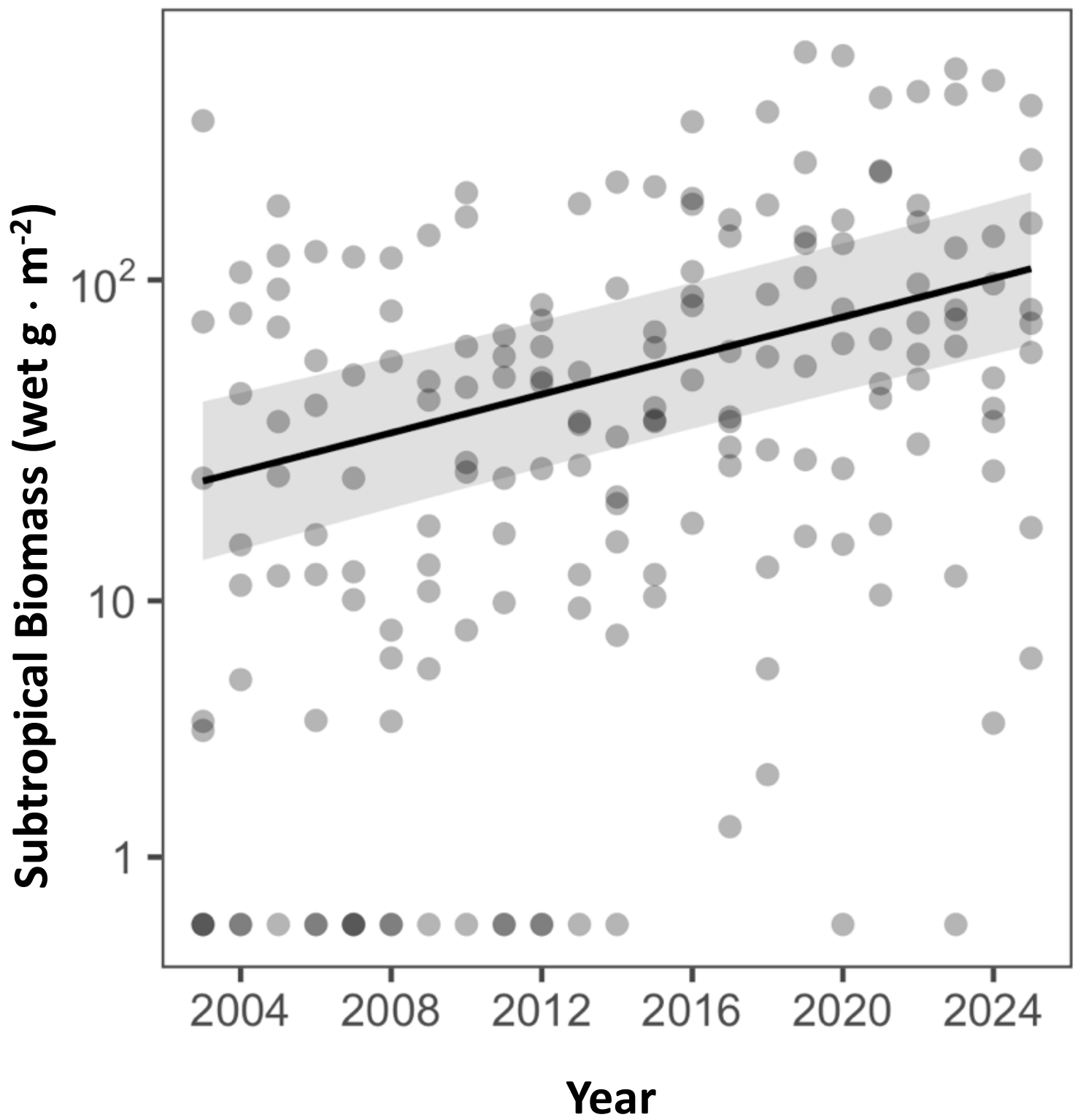

Supplementary Figure 13. Results of Model 5. Subtropical species biomass (mean wet g · m^{-2} by site) as a function of year. The solid line represents the results of generalized linear mixed effects modeling while the grey shading indicates the 95% confidence interval. Subtropical species biomass is represented on a log scale.

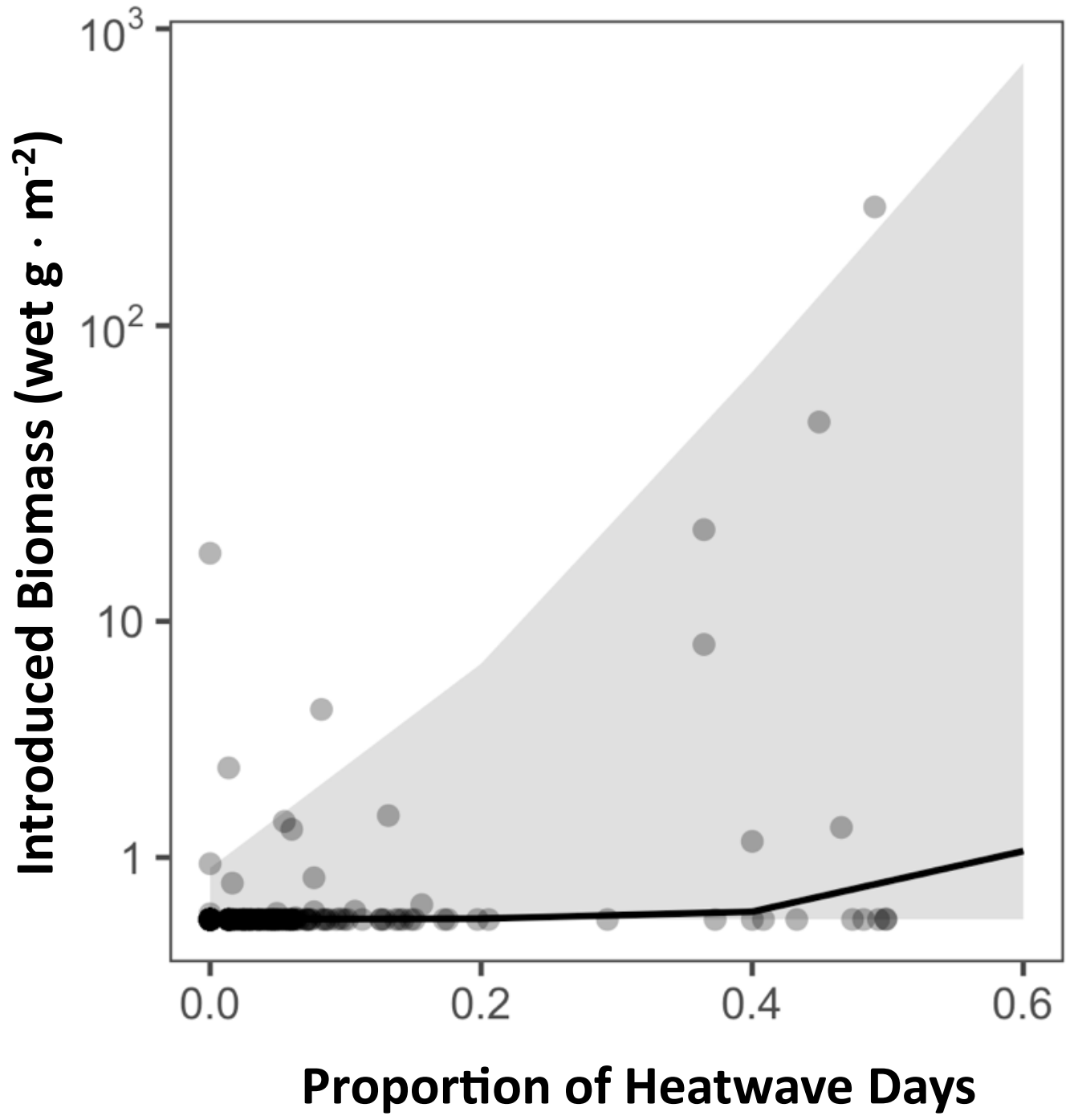

Supplementary Figure 14. Results of Model 7. Introduced species biomass (mean wet g · m^{-2} by site) as a function of the proportion of heatwave days per year. The solid line represents the results of generalized linear mixed effects modeling while the grey shading indicates the 95% confidence interval. Introduced species biomass is represented on the log scale.

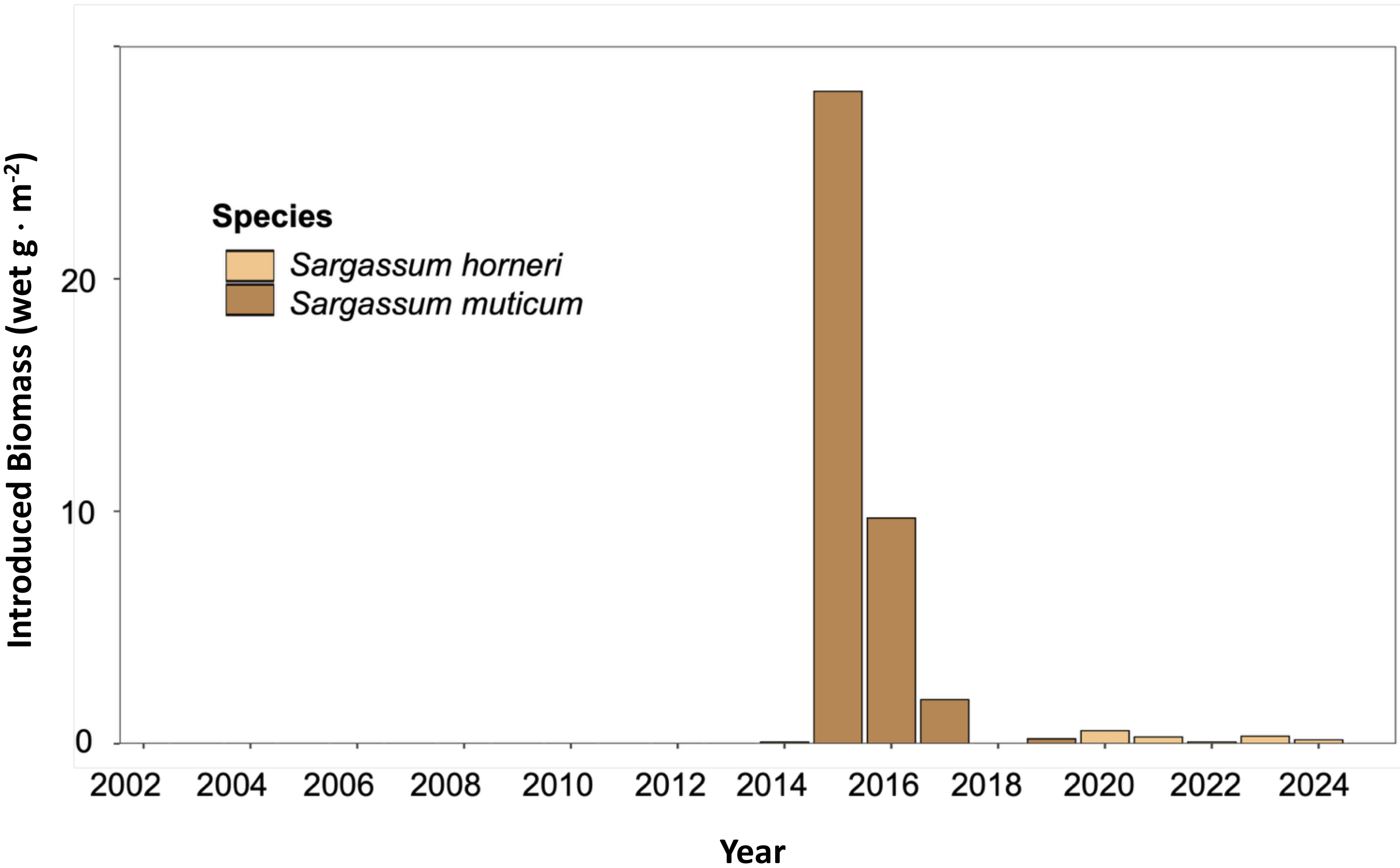

Supplementary Figure 15. Mean biomass (wet g · m^{-2}) of introduced species of *Sargassum* averaged across the 9 sites.

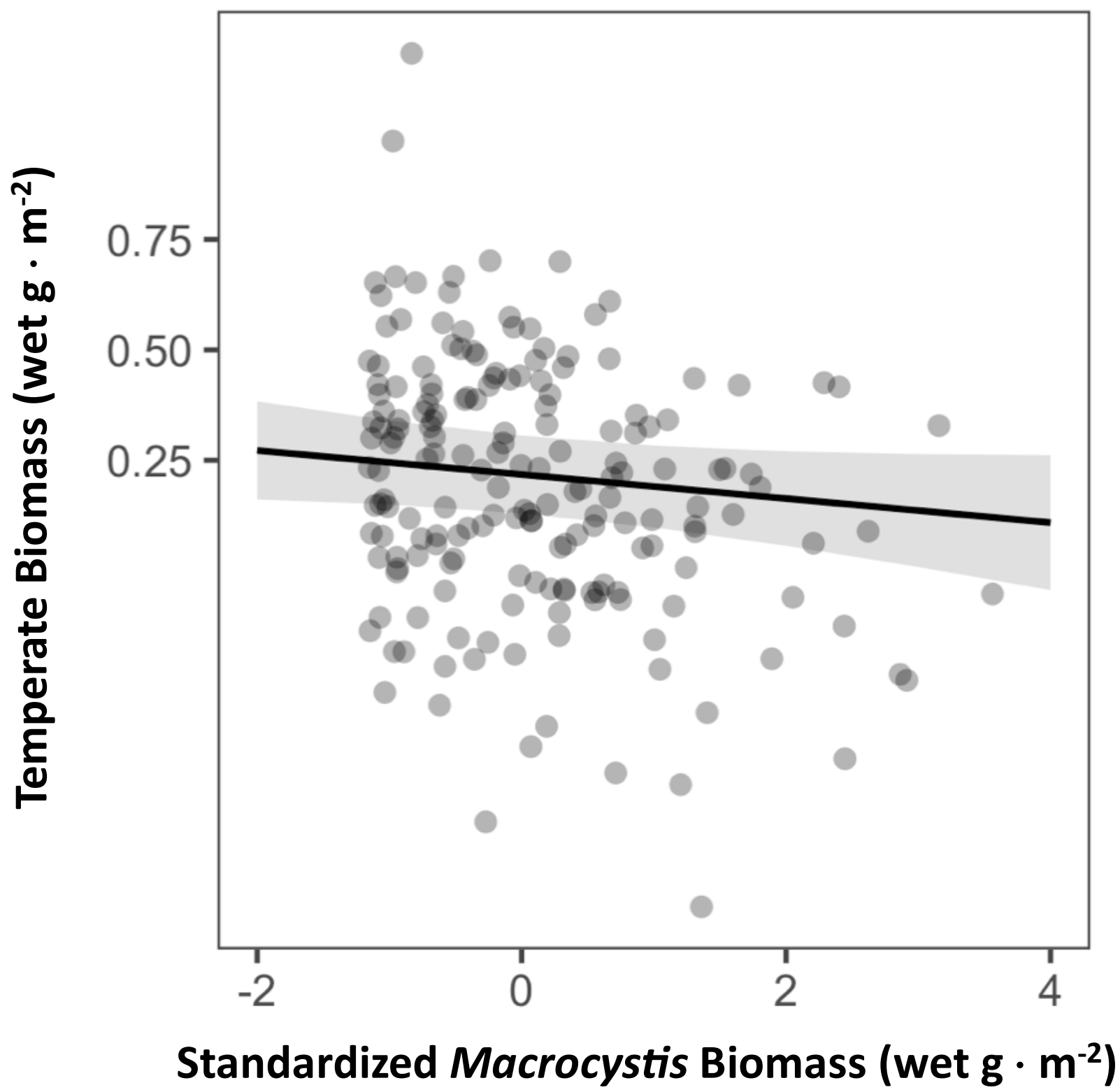

Supplementary Figure 16. Results of Model 8. Temperate species biomass (mean wet g · m^{-2} by site) as a function of *Macrocystis pyrifera* biomass (wet g · m^{-2}) which has been standardized to a mean of 0 and standard deviation of 1. The solid line represents the results of generalized linear mixed effects modeling while the grey shading indicates the 95% confidence interval. Temperate species biomass has been transformed to fall between 0 and 1 in compliance with the beta distribution and is represented along the logit scale.

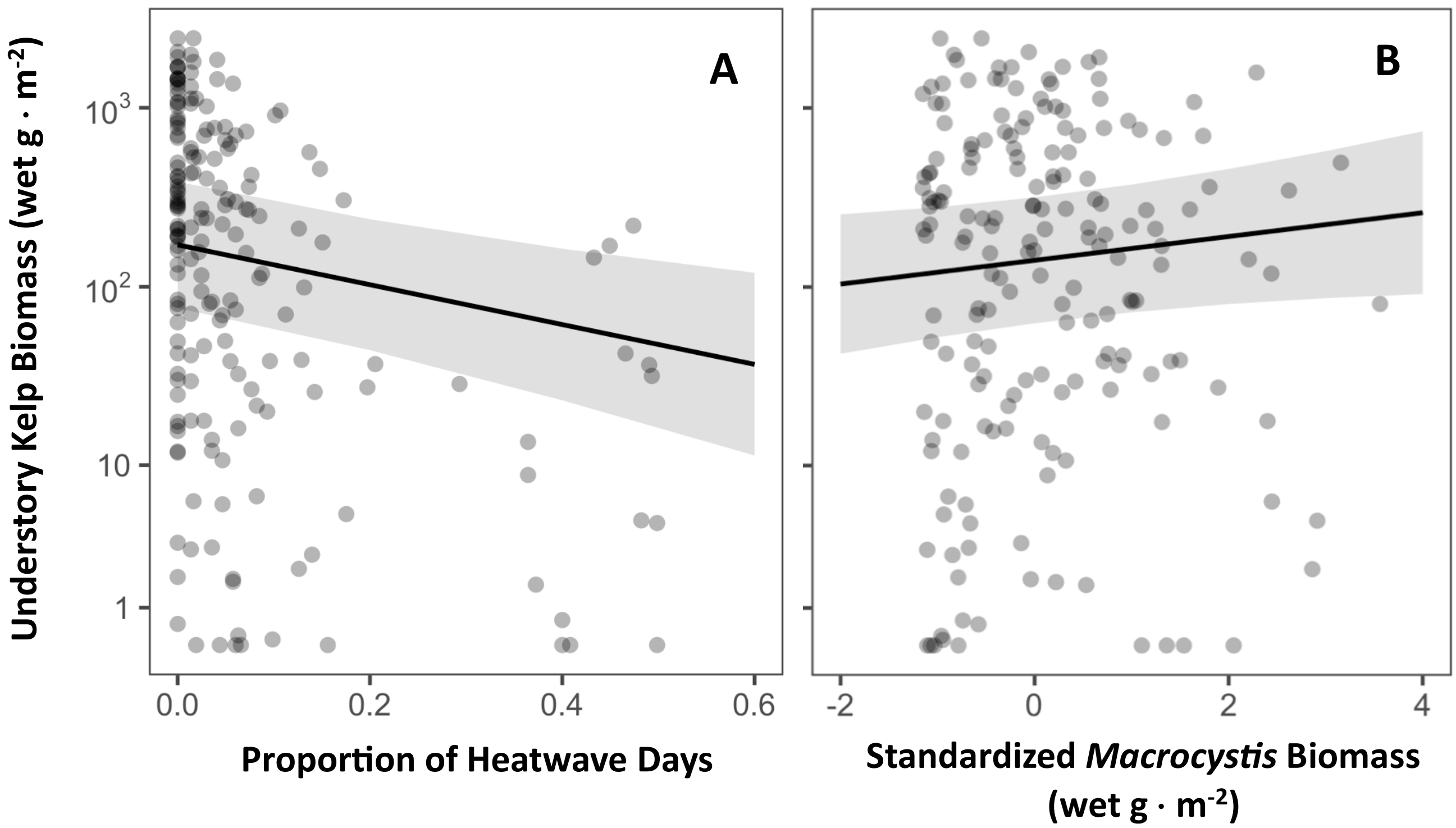

Supplementary Figure 17. Results of Model 9. Understory kelp biomass (mean wet g · m^{-2} by site) as a function of A) the proportion of heatwave days per year and B) *Macrocystis pyrifera* biomass (wet g · m^{-2}) which has been standardized to a mean of 0 and standard deviation of 1. The solid line represents the results of generalized linear mixed effects modeling while the grey shading indicates the 95% confidence interval. Understory kelp biomass is represented on the log scale.

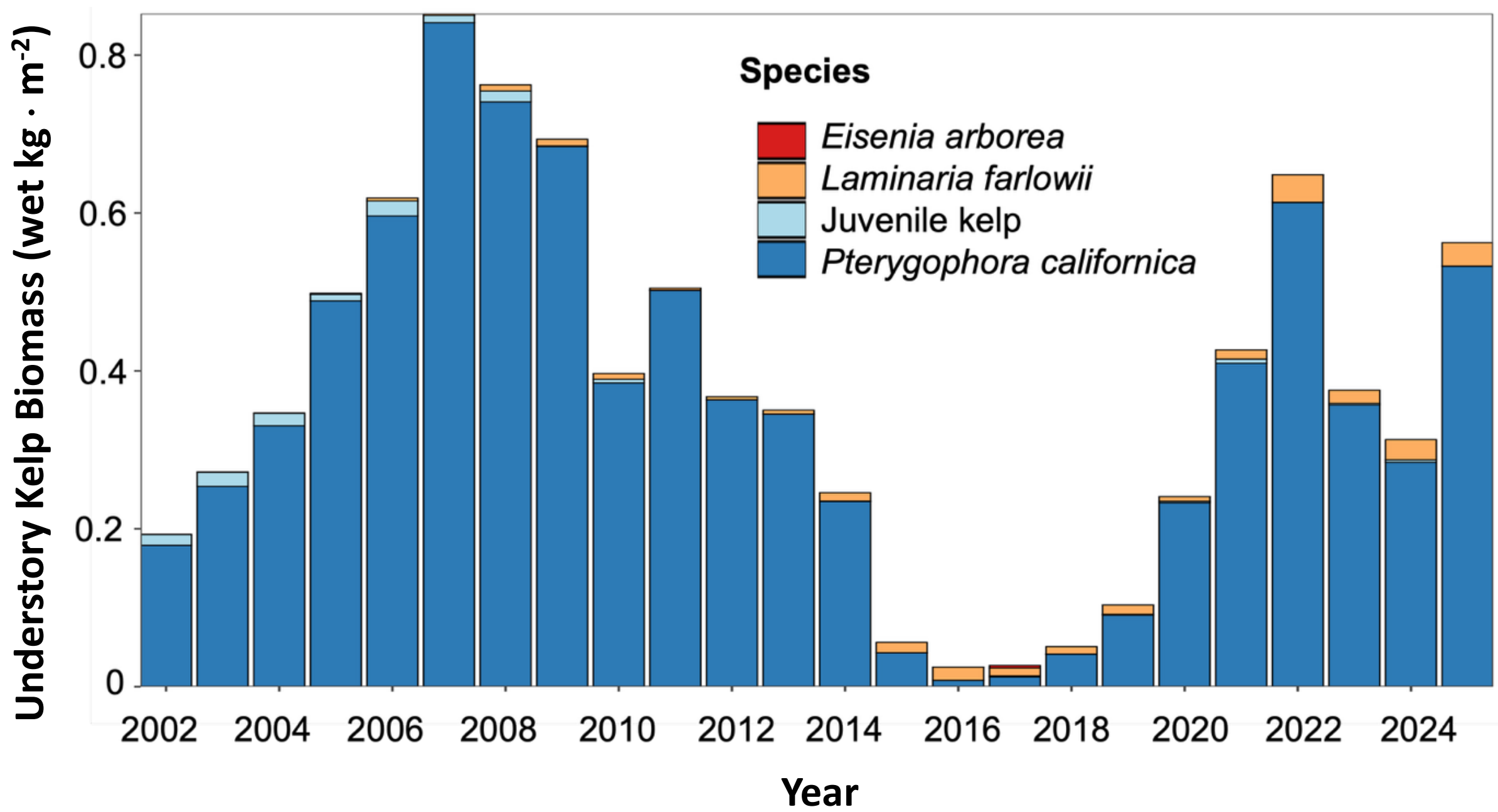

Supplementary Figure 18. Mean biomass (wet kg · m^{-2}) of understory kelp species averaged across the 9 sites.

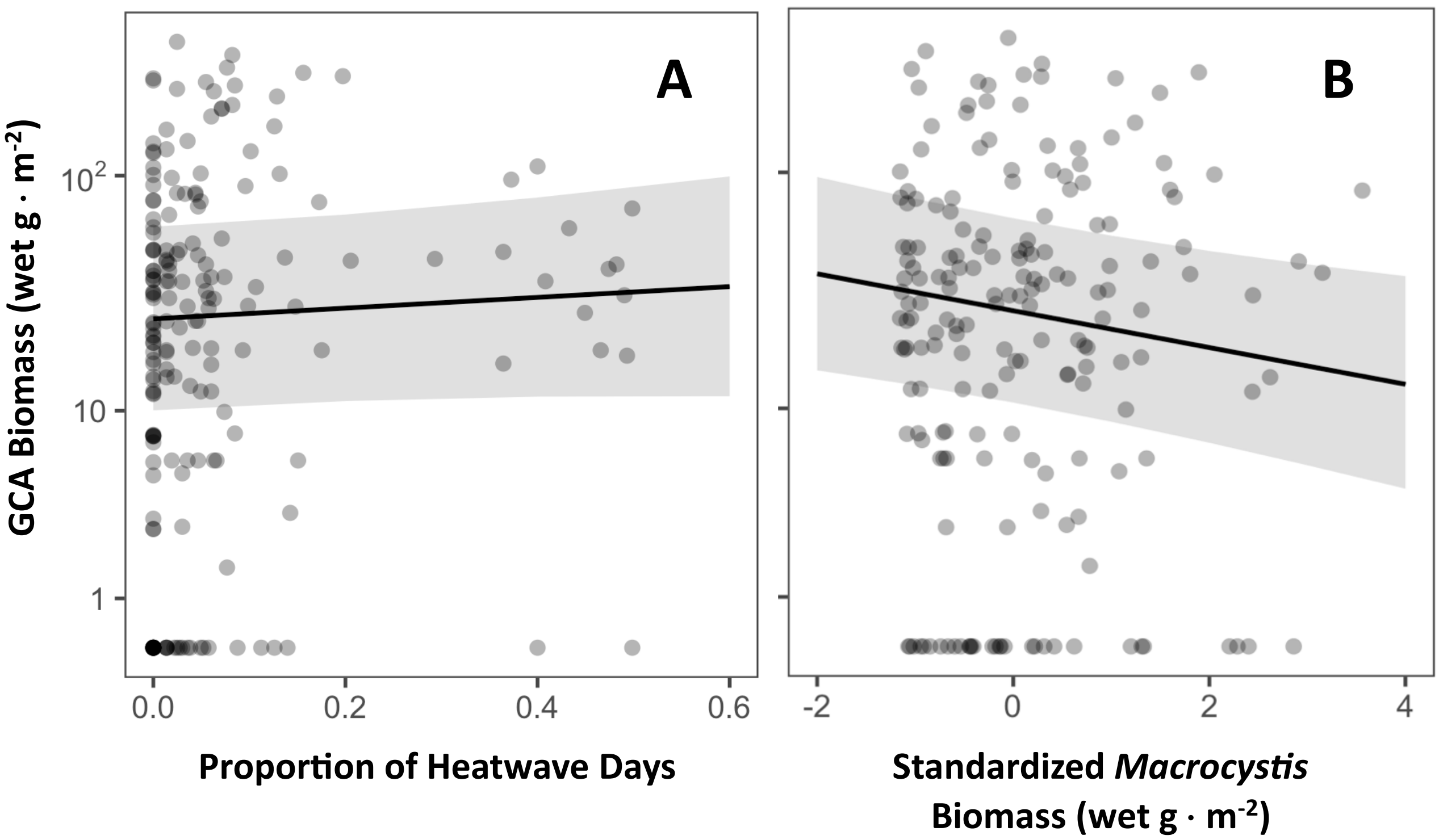

Supplementary Figure 19. Results of Model 6. GCA biomass (mean wet g · m^{-2} by site) as a function of A) the proportion of heatwave days and B) *Macrocystis pyrifera* biomass (wet g · m^{-2}) which has been standardized to a mean of 0 and standard deviation of 1. The solid line represents the results of generalized linear mixed effects modeling while the grey shading indicates the 95% confidence interval. GCA biomass is represented on the log scale.

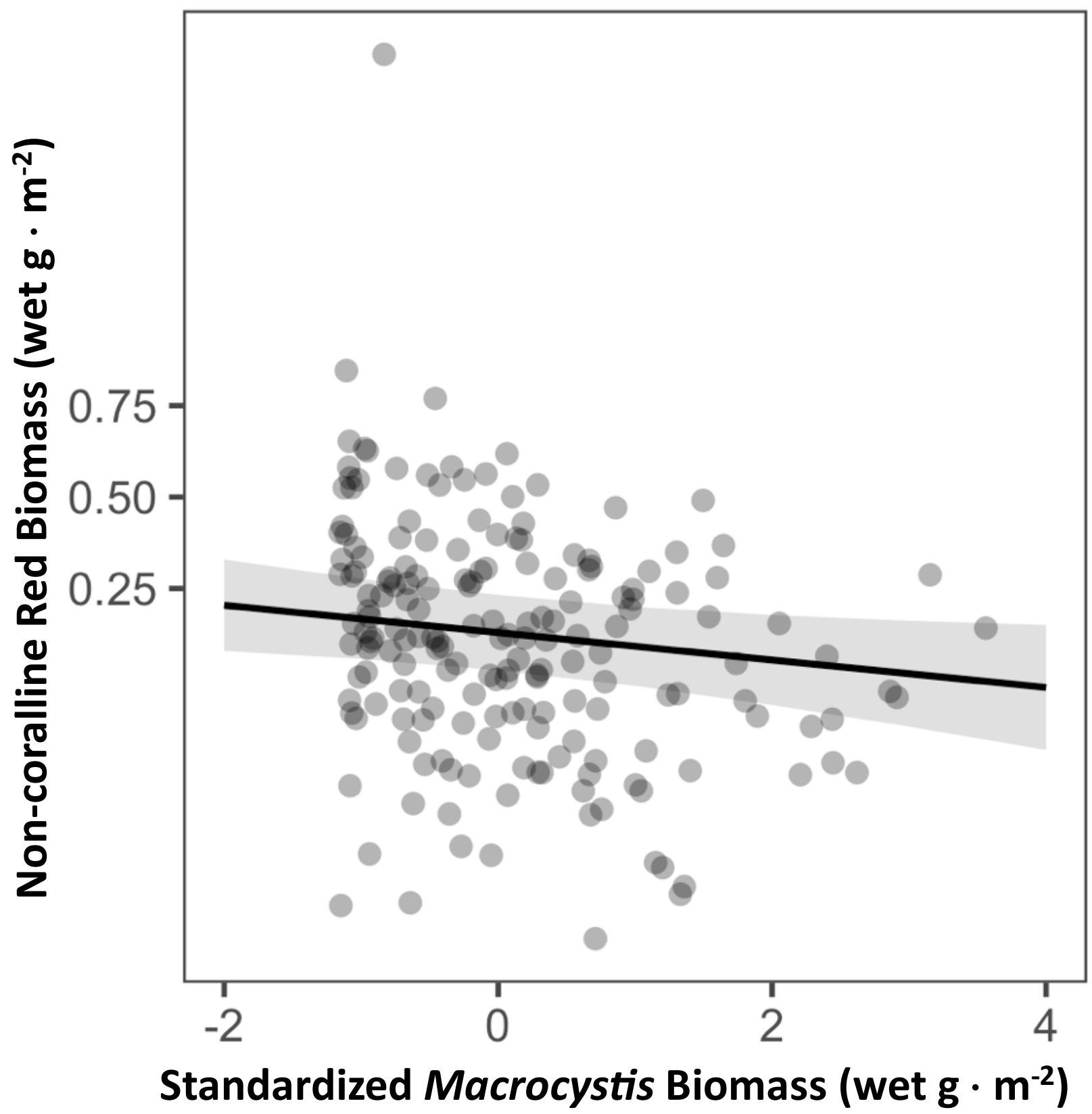

Supplementary Figure 20. Results of Model 13. Non-coralline red algal biomass (mean wet g · m^{-2} by site) as a function of *Macrocystis pyrifera* biomass (wet g · m^{-2}) which has been standardized to a mean of 0 and standard deviation of 1. The solid line represents the results of generalized linear mixed effects modeling while the grey shading indicates the 95% confidence interval. Non-coralline red algal biomass has been transformed to fall between 0 and 1 in compliance with the beta distribution and is represented on the logit scale.

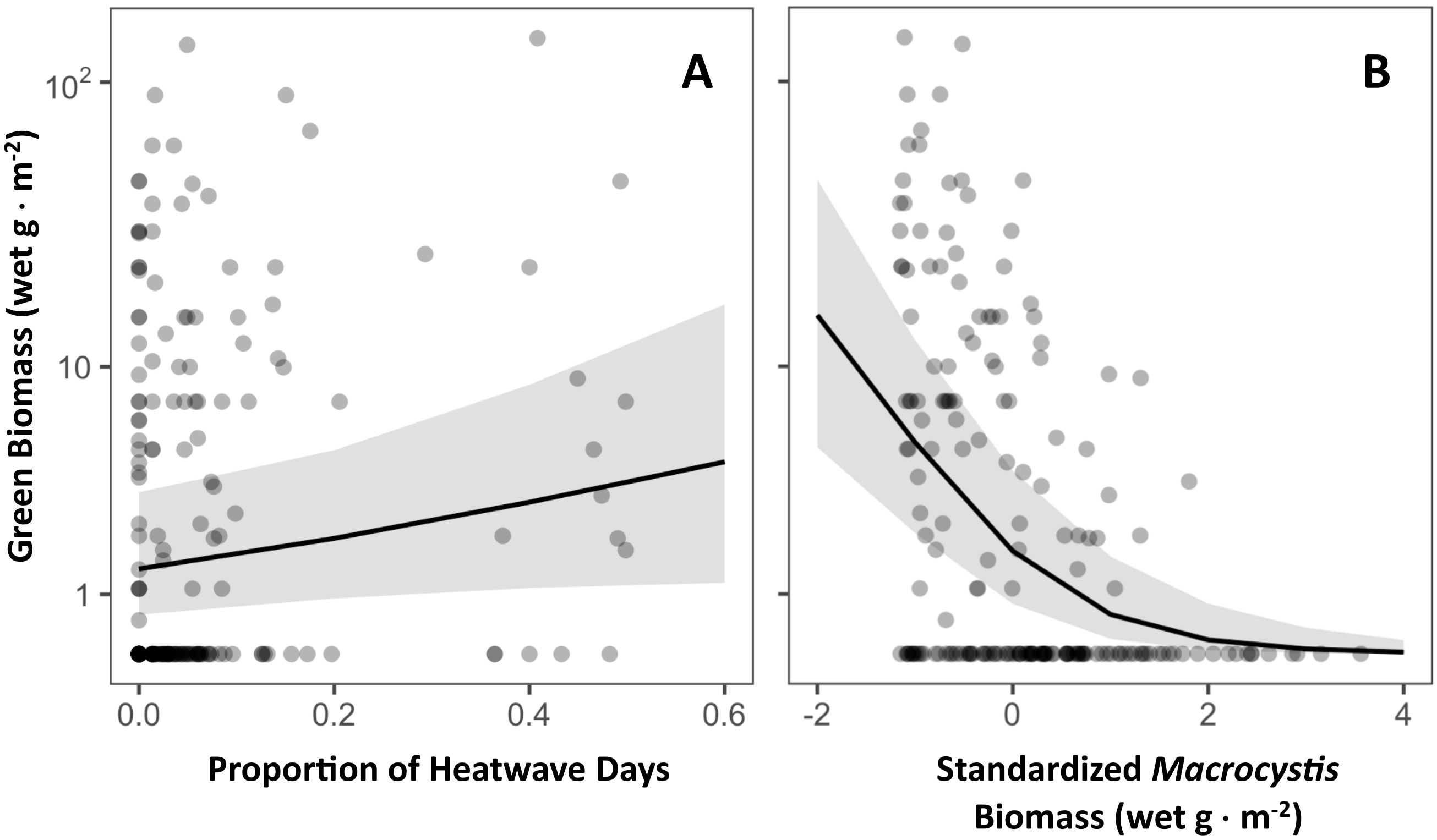

Supplementary Figure 21. Results of Model 14. Green algal species biomass (mean wet g · m^{-2} by site) as a function of A) the proportion of heatwave days per year and B) *Macrocystis pyrifera* biomass (wet g · m^{-2}) which has been standardized to a mean of 0 and standard deviation of 1. Solid lines represent the results of generalized linear mixed effects modeling while the grey shading indicates the 95% confidence interval. Green algal biomass is represented on the log scale.

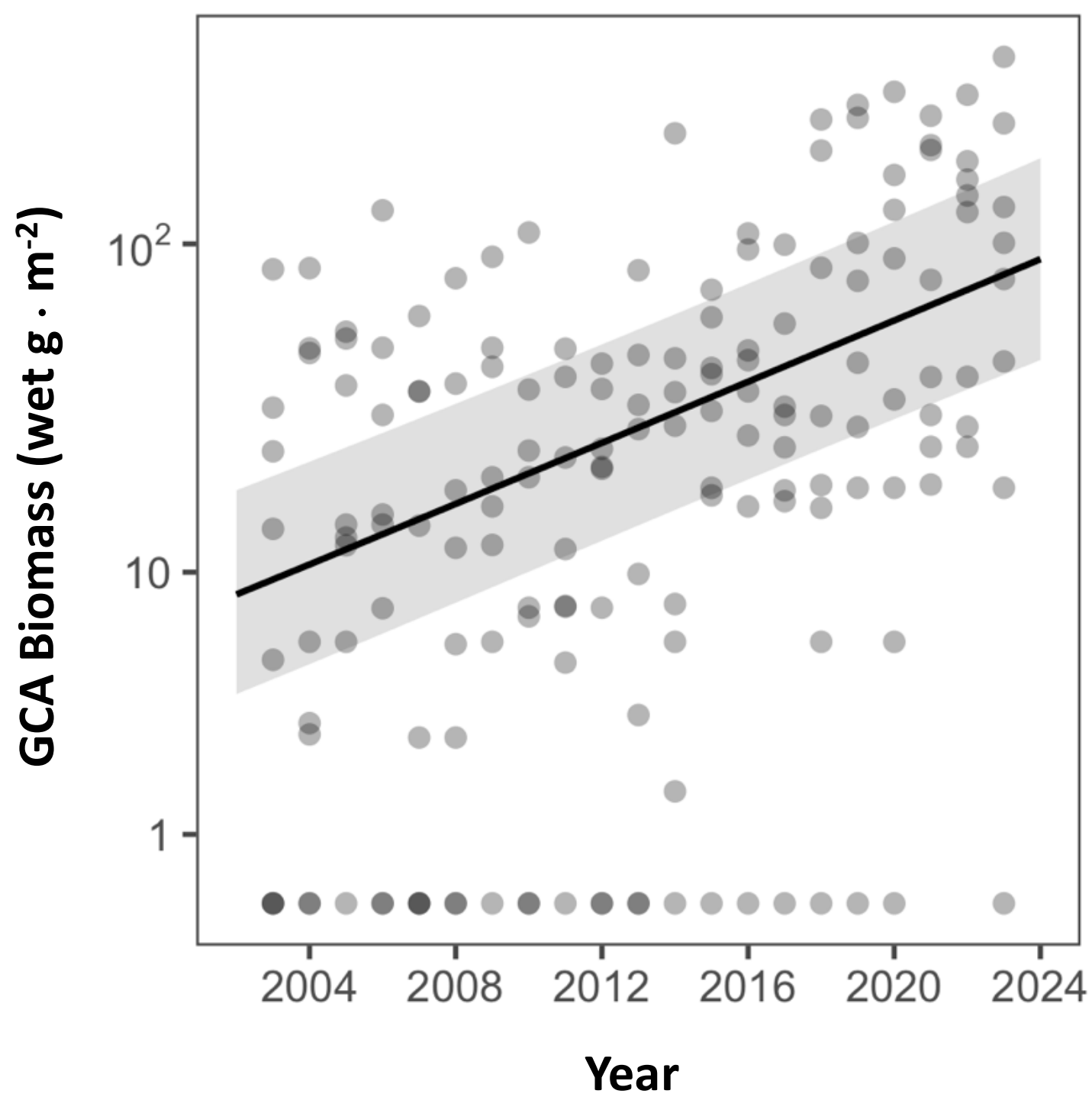

Supplementary Figure 22. Results of Model 10. GCA biomass (mean wet g · m^{-2} by site) as a function of year. The solid line represents the results of generalized linear mixed effects modeling while the grey shading indicates the 95% confidence interval. GCA biomass is represented on the log scale.

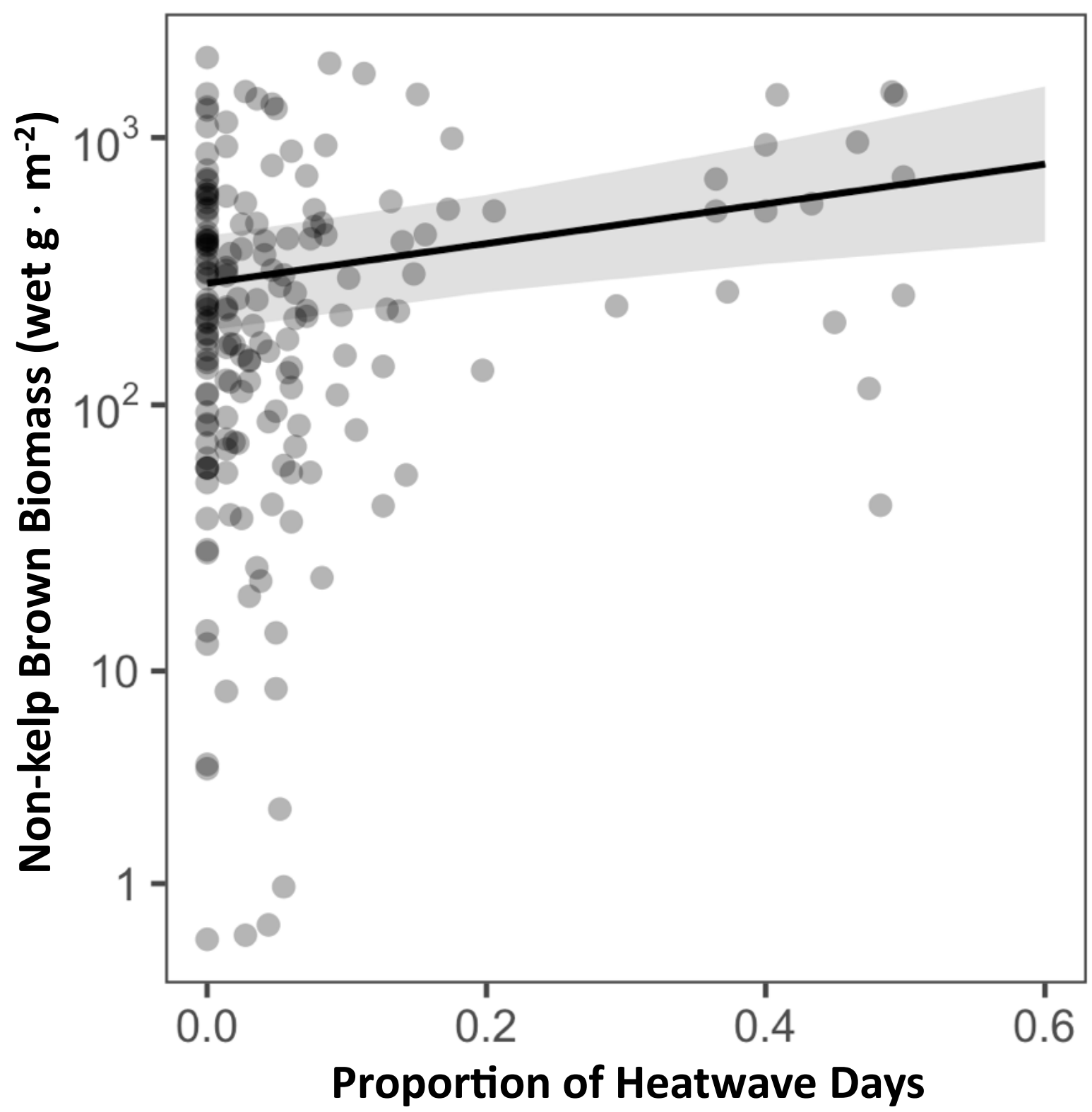

Supplementary Figure 23. Results of Model 12. Non-kelp brown algal biomass (mean wet g · m^{-2} by site) as a function of the proportion of heatwave days per year by site. The solid line represents the results of generalized linear mixed effects modeling while the grey shading indicates the 95% confidence interval. Non-kelp brown algal biomass is represented on the log scale.

Supplementary Note 1: Site Descriptions

Bulito reef is characterized by moderate bedrock relief and is on average the coldest site, with the greatest influence of cooler California Current waters (Fig. 5). Arroyo Hondo, Arroyo Quemada, and Arroyo Burro reefs are each situated at the mouth of creeks and are characterized by high variability in sand inundation, fine sediment inputs, and relatively low relief bedrock. Naples Reef is further offshore than the other sites and has high relief and the highest average flow. Both Naples and Isla Vista Reef, characterized by slight to moderate relief mudstone, became State Marine Conservation Areas in 2012. Goleta Bay Reef is a low relief site with some large boulders that experiences high sediment inputs from the neighboring Goleta Slough watershed and is situated within a relatively protected bay. Mohawk Reef is a moderate relief reef and transects are situated along overhanging bedrock shelves. Carpinteria, the warmest and most eastern reef, is characterized by moderate relief and estuarine inputs from the neighboring Carpinteria Salt Marsh.

Supplementary Table 2. Functional and thermal groups of species surveyed by the SBC LTER. Species that are difficult to distinguish visually by divers have multiple possible species listed.

**Phylum**	**Order**	**Family**	**Species**	**Functional Group**	**Thermal Group**
Rhodophyta	Florideophyceae	Delesseriaceae	*Acrosorium ciliolatum*	non-coralline red	subtropical
Rhodophyta	Florideophyceae	Lithophyllaceae	*Amphiroa beauvoisii*	geniculate coralline	subtropical
Rhodophyta	Florideophyceae	Delesseriaceae	*Anisocladella pacifica*	non-coralline red	transitional
Rhodophyta	Florideophyceae	Corallinaceae	*Bossiella orbigniana*	geniculate coralline	temperate
Rhodophyta	Florideophyceae	Corallinaceae	*Calliarthron cheilosporioides*	geniculate coralline	transitional
Rhodophyta	Florideophyceae	Kallymeniaceae	*Callophyllis flabellulata*	non-coralline red	temperate
Rhodophyta	Florideophyceae	Gigartinaceae	*Chondracanthus corymbiferus*<br>*Chondracanthus exasperatus*	non-coralline red	temperate
Rhodophyta	Florideophyceae	Gigartinaceae	*Chondracanthus spinosus*	non-coralline red	transitional
Chlorophyta	Ulvophyceae	Cladophoraceae	*Cladophora graminea*	green	transitional
Chlorophyta	Ulvophyceae	Codiaceae	*Codium fragile*	green	subtropical
Ochrophyta	Phaeophyceae	Scytosiphonaceae	*Colpomenia peregrina*	non-kelp brown	temperate
Rhodophyta	Florideophyceae	Corallinaceae	*Corallina officinalis*	geniculate coralline	subtropical
Rhodophyta	Florideophyceae	Delesseriaceae	*Cryptopleura ruprechtiana*	non-coralline red	temperate
Rhodophyta	Florideophyceae	Delesseriaceae	*Cryptopleura* spp.	non-coralline red	temperate
Ochrophyta	Phaeophyceae	Desmarestiaceae	*Desmarestia ligulata*	non-kelp brown	temperate
Ochrophyta	Phaeophyceae	Dictyotaceae	*Dictyopteris undulata*	non-kelp brown	subtropical
Ochrophyta	Phaeophyceae	Dictyotaceae	*Dictyota binghamiae*<br>*Dictyota flabellate*<br>*Dictyota coriacea*	non-kelp brown	subtropical
Ochrophyta	Phaeophyceae	Ectocarpaceae	*Ectocarpaceae* spp.	filamentous	unknown
Ochrophyta	Phaeophyceae	Lessoniaceae	*Egregia menziesii*	overstory kelp	temperate
Ochrophyta	Phaeophyceae	Lessoniaceae	*Eisenia arborea*	understory kelp	temperate
Rhodophyta	Florideophyceae	Gelidiaceae	*Gelidium* spp.	non-coralline red	temperate
Rhodophyta	Florideophyceae	Faucheaceae	*Gloiocladia fryeana*	non-coralline red	temperate
Rhodophyta	Florideophyceae	Gracilariaceae	*Gracilaria* spp.	non-coralline red	temperate
Rhodophyta	Florideophyceae	Phyllophoraceae	*Gymnogongrus* spp.	non-coralline red	temperate
Rhodophyta	Florideophyceae	Palmariaceae	*Halosaccion glandiforme*	non-coralline red	northern-temperate
Rhodophyta	Florideophyceae		*Halymenia* spp.<br>*Schizymenia pacifica*	non-coralline red	temperate

Phylum	Order	Family	Species	Functional Group	Thermal Group
Rhodophyta	Florideophyceae	Hildenbrandiaceae	*Hildenbrandia* spp.	non-kelp brown	unknown
Ochrophyta	Phaeophyceae	Laminariaceae	*Laminaria farlowii*	understory kelp	temperate
Rhodophyta	Florideophyceae	Rhodomelaceae	*Laurencia* spp.	non-coralline red	subtropical
Rhodophyta	Florideophyceae	Lithophyllaceae	*Lithothrix aspergillum*	geniculate coralline	temperate
Ochrophyta	Phaeophyceae	Laminariaceae	*Macrocystis pyrifera*	overstory kelp	temperate
Rhodophyta	Florideophyceae	Gigartinaceae	*Mazzaella* spp.	non-coralline red	temperate
Rhodophyta	Florideophyceae		*Neoptilota* spp.<br>*Ptilota* spp.<br>*Rhodoptilum* spp.	non-coralline red	temperate
Rhodophyta	Florideophyceae	Delesseriaceae	*Nienburgia andersoniana*	non-coralline red	temperate
Rhodophyta	Florideophyceae	Rhodomelaceae	*Osmundea spectabilis*	non-coralline red	northern-temperate
Rhodophyta	Florideophyceae	Delesseriaceae	*Phycodrys setchellii*	non-coralline red	temperate
Rhodophyta	Florideophyceae	Delesseriaceae	*Polyneura latissima*	non-coralline red	temperate
Rhodophyta	Florideophyceae	Halymeniaceae	*Prionitis lanceolata*	non-coralline red	temperate
Rhodophyta	Florideophyceae	Halymeniaceae	*Prionitis linearis*	non-coralline red	temperate
Ochrophyta	Phaeophyceae	Alariaceae	*Pterygophora californica*	understory kelp	temperate
Rhodophyta	Florideophyceae	Rhodymeniaceae	*Rhodymenia californica*	non-coralline red	temperate
Rhodophyta	Florideophyceae	Solieriaceae	*Sarcodiotheca furcata*	non-coralline red	subtropical
Ochrophyta	Phaeophyceae	Sargassaceae	*Sargassum horneri*	non-kelp brown	introduced
Ochrophyta	Phaeophyceae	Sargassaceae	*Sargassum muticum*	non-kelp brown	introduced
Rhodophyta	Florideophyceae	Scinaiaceae	*Scinaia confusa*	non-coralline red	temperate
Ochrophyta	Phaeophyceae	Scytosiphonaceae	*Scytosiphon lomentaria*	non-kelp brown	temperate
Rhodophyta	Florideophyceae	Phyllophoraceae	*Stenogramma californicum*	non-coralline red	temperate
Ochrophyta	Phaeophyceae	Sargassaceae	*Stephanocystis osmundacea*	non-kelp brown	transitional
Ochrophyta	Phaeophyceae	Dictyotaceae	*Taonia lennebackerae*	non-kelp brown	subtropical
Chlorophyta	Ulvophyceae	Ulvaceae	*Ulva* spp.	green	unknown
Chlorophyta	Ulvophyceae		*Sponogomorpha* spp.	green	northern-temperate
Rhodophyta			Unidentifiable Branching Red Alga	non-coralline red	unknown
Ochrophyta	Phaeophyceae	Lessoniaceae	Unidentifiable juvenile kelp	kelp	unknown
Ochrophyta	Phaeophyceae		Unidentifiable small brown blade	non-kelp brown	unknown
Rhodophyta	Florideophyceae	Ceramiaceae	Unknown Ceramiaceae spp.	filamentous	unknown
Rhodophyta			Unknown Rhodophyta	filamentous	unknown

Supplementary Table 3. Parameters for all generalized linear models, generalized linear mixed effects models, and linear mixed effects models.

**Model**	**Response**	**Response Distribution**	**Predictors**	**Notes**
1	Total understory biomass	Gaussian	*Macrocystis* biomass (scaled)	Response has been square root transformed; the proportion of heatwave days was not a significant predictor, reduced model fit when included, and was excluded from the final model
2	Total understory richness	Gaussian	Proportion of heatwave days<br>*Macrocystis* biomass (scaled)	Minor issues with residuals, no clear pattern; dispersion term does not improve residuals; best fitting model with least residual issues
3	*Macrocystis* biomass	Beta	Proportion of heatwave days	Model was not significantly better than the null model, as the proportion of heatwave days was not a significant predictor
4	Subtropical species biomass	Tweedie	Proportion of heatwave days<br>*Macrocystis* biomass (scaled)	
5	Subtropical species biomass	Tweedie	Year	
6	Transitional species biomass	Beta	Proportion of heatwave days<br>*Macrocystis* biomass (scaled)	Dispersion term for *Macrocystis* biomass improves model fit and residuals
7	Introduced species biomass	Tweedie	Proportion of heatwave days	No autocorrelation term in model, model would not converge; *Macrocystis* biomass was removed from final model as it decreased model fit and was not a significant predictor
8	Temperate species biomass	Beta	*Macrocystis* biomass (scaled)	The proportion of heatwave days was removed from the final model to improve model AIC, but the model was still not significantly better than the null model
9	Understory kelp biomass	Tweedie	Proportion of heatwave days<br>*Macrocystis* biomass (scaled)	Mild issues with residuals for all possible response distributions, but no clear pattern; including AR structure improves model fit
10	GCA biomass	Tweedie	Year	
11	GCA biomass	Tweedie	Proportion of heatwave days<br>*Macrocystis* biomass (scaled)	
12	Non-kelp brown biomass	Tweedie	Proportion of heatwave days	No autocorrelation term in model, as model would not converge with the term included
13	Non-coralline red biomass	Beta	*Macrocystis* biomass (scaled)	Minor issues with model residuals but no clear pattern, Proportion of heatwave days decreased model fit and did not improve residual issues
14	Green biomass	Tweedie	Proportion of heatwave days<br>*Macrocystis* biomass (scaled)	Minor issues with model residuals but no clear pattern
