# Supplementary material for: Macroalgal community transformation during successive marine heatwaves in southern California kelp forests
Source: Commun Earth Environ. 2026 May 16;7(1):632. doi: 10.1038/s43247-026-03599-5 (PMC13437228; doi:10.1038/s43247-026-03599-5)
Supplement: Supplementary file 3 [file 43247_2026_3599_MOESM3_ESM.pdf]

nature portfolio

Corresponding author(s): Kristen Michaud

Last updated by author(s): 04/16/2026

Reporting Summary

Nature Portfolio wishes to improve the reproducibility of the work that we publish. This form provides structure for consistency and transparency in reporting. For further information on Nature Portfolio policies, see our Editorial Policies and the Editorial Policy Checklist.

Statistics

For all statistical analyses, confirm that the following items are present in the figure legend, table legend, main text, or Methods section.

n/a	Confirmed	
☐	☒	The exact sample size (*n*) for each experimental group/condition, given as a discrete number and unit of measurement
☐	☒	A statement on whether measurements were taken from distinct samples or whether the same sample was measured repeatedly
☐	☒	The statistical test(s) used AND whether they are one- or two-sided<br>*Only common tests should be described solely by name; describe more complex techniques in the Methods section.*
☐	☒	A description of all covariates tested
☐	☒	A description of any assumptions or corrections, such as tests of normality and adjustment for multiple comparisons
☐	☒	A full description of the statistical parameters including central tendency (e.g. means) or other basic estimates (e.g. regression coefficient) AND variation (e.g. standard deviation) or associated estimates of uncertainty (e.g. confidence intervals)
☐	☒	For null hypothesis testing, the test statistic (e.g. *F*, *t*, *r*) with confidence intervals, effect sizes, degrees of freedom and *P* value noted<br>*Give P values as exact values whenever suitable.*
☒	☐	For Bayesian analysis, information on the choice of priors and Markov chain Monte Carlo settings
☒	☐	For hierarchical and complex designs, identification of the appropriate level for tests and full reporting of outcomes
☐	☒	Estimates of effect sizes (e.g. Cohen's *d*, Pearson's *r*), indicating how they were calculated

Our web collection on statistics for biologists contains articles on many of the points above.

Software and code

Policy information about availability of computer code

Data collection	No software was used.
Data analysis	All code used to perform this analysis is publicly available from: Michaud, K. M. Macroalgal community analysis Michaud et al. 2026 ver 2. Zenodo. https:// 10.5281/zenodo.19617116 (2025).

For manuscripts utilizing custom algorithms or software that are central to the research but not yet described in published literature, software must be made available to editors and reviewers. We strongly encourage code deposition in a community repository (e.g. GitHub). See the Nature Portfolio guidelines for submitting code & software for further information.

Data

Policy information about availability of data

All manuscripts must include a data availability statement. This statement should provide the following information, where applicable:

- Accession codes, unique identifiers, or web links for publicly available datasets
- A description of any restrictions on data availability
- For clinical datasets or third party data, please ensure that the statement adheres to our policy

All data used in our analysis is publicly available from the Santa Barbara Long Term Ecological Research program:
Reed, D. C. & Miller, R. J. SBC LTER: reef: bottom temperature: continuous water temperature, ongoing since 2000 ver 33. Environmental Data Initiative (2026). doi:10.6073/pasta/e565d409b63f20c768133c653ccdb2d7.

Reed, D. C. & Miller, R. J. SBC LTER: Reef: Annual time series of biomass for kelp forest species, ongoing since 2000 ver 18. Environmental Data Initiative (2025). doi:10.6073/pasta/cb45bf15430ba570828444b13dd8521f

Research involving human participants, their data, or biological material

Policy information about studies with human participants or human data. See also policy information about sex, gender (identity/presentation), and sexual orientation and race, ethnicity and racism.

Reporting on sex and gender	N/A
Reporting on race, ethnicity, or other socially relevant groupings	N/A
Population characteristics	N/A
Recruitment	N/A
Ethics oversight	N/A

Note that full information on the approval of the study protocol must also be provided in the manuscript.

Field-specific reporting

Please select the one below that is the best fit for your research. If you are not sure, read the appropriate sections before making your selection.

☐ Life sciences ☐ Behavioural & social sciences ☒ Ecological, evolutionary & environmental sciences

For a reference copy of the document with all sections, see nature.com/documents/nr-reporting-summary-flat.pdf

Ecological, evolutionary & environmental sciences study design

All studies must disclose on these points even when the disclosure is negative.

Study description	This study utilized long term monitoring data from surveys performed by the Santa Barbara Coastal Long Term Ecological Research (SBC LTER) program and temperature data from in situ bottom temperature loggers. We used this long term data to assess how understory macroalgal functional groups and species with similar thermal tolerances responded to years with many heatwave days. We used generalized linear and linear mixed effects models to asses whether the proportion of heatwave days in a given year and the biomass of the overstory kelp species, giant kelp, predicted the biomass of each understory functional and thermal group. We used a constrained ordination analysis, Canonical Analysis of Principal Coordinates, to assess whether understory community composition shifted during the strong heatwave years from 2015-2019. Similarly, we used PERMANOVA to assess whether these community changes were significant.
Research sample	This study utilized two existing datasets from the SBC LTER. Surveys of kelp forest benthic communities are performed annually at 9 sites along the mainland coast of the Santa Barbara Channel. Each site has 2-8 permanent 2 meter x 40 meter transects depending on the total area of the rocky reef. At each location, a combination of uniform point contact surveys, quadrat surveys, and swath surveys are used to estimate the abundance and biomass of kelp forest biota, which is reported as the annual biomass of each species by transect.
Sampling strategy	This study assessed biomass of 62 taxa of surveyed understory macroalgae at 9 kelp forest sites. Biomass was represented as a time series beginning in 2003, when continuous bottom temperature data was available at each of the 9 sites, to 2025, resulting in 23 repeated measures. All linear and generalized linear mixed effects models included temporal autocorellation terms within site to account for repeated measures. Sample size was not explicitly chosen and instead represented all available data across the time series.
Data collection	Divers used a combination of swath, quadrat, and uniform point contact surveys to estimate abundance and biomass of 62 taxa of understory and canopy forming macroalgae (Supplementary Table 2). Laminarian kelps including Macrocystis pyrifera, Laminaria farlowii, Pterygophora californica, Eisenia arborea and the fucoid Stephanocystis osmundacea were counted and sized along fixed 40m x 2m transects by divers. The abundances of juvenile kelps and the non-native fucoids Sargassum horneri and Sargassum muticum were measured by counting and sizing individuals within 6 fixed 1m2 quadrats uniformly spaced along the transect. The abundance of smaller species and those that are difficult to distinguish as individuals were measured using percent cover estimates from 80 uniformly spaced points along the transect.<br>For Macrocystis pyrifera, we converted measurements of frond density (no. per transect) to estimates of wet biomass density (g m-2) using allometric relationships developed for each month of the year. For all other species, we used taxon-specific allometric relationships to convert measurements of size-specific density or percent cover to wet biomass. Wet biomass was then averaged across transects to obtain wet biomass by species at each site per year from 2003 to 2025.
Timing and spatial scale	Data collection began in 2000 at some sites and by 2003, all 9 mainland sites were surveyed annually in July of each year. The

Timing and spatial scale	presented time series spanned 2003 to 2025, which were years where complete bottom temperature data was available for all 9 sites. Sites were located along 78km of the mainland coast of the Santa Barbara Channel.
Data exclusions	For most generalized linear mixed effects models, the Arroyo Hondo site was excluded due to anomalously low biomass values for all surveyed taxa. This site experienced repeated years of sand inundation in later sampling years. Exclusion of this site drastically improved model fit. All model parameters, response distributions, and notes are included in Supplementary Table 3. All model results can be found in Table 1 and Supplementary Table 1.
Reproducibility	All code is publicly available for reproduction of our analyses.
Randomization	This was not relevant to our observational study.
Blinding	This was not relevant to our observational study.

Did the study involve field work? ☒ Yes ☐ No

Field work, collection and transport

Field conditions	All surveys were performed by AAUS scientific SCUBA divers from University of California Santa Barbara and Santa Barbara Coastal Long Term Ecological Research program owned small boats. Bottom water temperature ranged from roughly 15C to 20C during July of each sampling year, though temperatures ranged from roughly 13C to 22C over the entire sampling period. Dives were between roughly 12 to 50 ft depending on the sampling site and transect. The Santa Barbara Channel and mainland are characterized by a Mediterranean climate with seasonal winter precipitation and warm, dry summers.
Location	Sites were located across ~ 78 km of the mainland coast of the Santa Barbara Channel (Bulito: 34.46 degrees N, 120.33 degrees W, Arroyo Hondo: 34.47 degrees N, 120.14 degrees W, Arroyo Quemada: 34.47 degrees N, 120.12 degrees W, Naples: 34.42 degrees N, 119.95 degrees W, Isla Vista: 34.40 degrees N, 119.86 degrees W, Goleta Bay: 34.41 degrees N, 119.82 degrees W, Arroyo Burro: 34.40 degrees N, 119.74 degrees W, Mohawk: 34.39 degrees N, 119.73 degrees W, Carpinteria: 34.39 degrees N, 119.54 degreesW; Fig. 5B). The number of permanent transects varied by site from 2 to 8 depending on the total area of the rocky reef. Average transect depth varied between 2.5 m and 11 m below mean lower low water (MLLW) across sites with a mean of 7 m.
Access & import/export	The data reported here is observational and all surveys were performed with minimal impact to the kelp forest substrate and/or biota. Permits were not required to collect survey data. All sites were accessed via small boats that are research vessels owned and operated by the University of California Santa Barbara and the Santa Barbara Coastal Long Term Ecological Research program.
Disturbance	This was an observational study involving survey data, and we attempted to inflict minimal disturbance to existing kelp forest biota and habitat.

Reporting for specific materials, systems and methods

We require information from authors about some types of materials, experimental systems and methods used in many studies. Here, indicate whether each material, system or method listed is relevant to your study. If you are not sure if a list item applies to your research, read the appropriate section before selecting a response.

Materials & experimental systems

n/a	Involved in the study	
☒	☐	Antibodies
☒	☐	Eukaryotic cell lines
☒	☐	Palaeontology and archaeology
☐	☒	Animals and other organisms
☒	☐	Clinical data
☒	☐	Dual use research of concern
☒	☐	Plants

Methods

n/a	Involved in the study	
☒	☐	ChIP-seq
☒	☐	Flow cytometry
☒	☐	MRI-based neuroimaging

Animals and other research organisms

Policy information about studies involving animals; ARRIVE guidelines recommended for reporting animal research, and Sex and Gender in Research

Laboratory animals	This study did not involve laboratory animals.
Wild animals	This study did not require capture or take of wild animals. We observed wild animals in situ during surveys, but did not alter any animals or remove them from their environment.

Reporting on sex	This information has not been collected.
Field-collected samples	This study did not involve samples collected from the field.
Ethics oversight	No ethical oversight or approval was required since we were only observing in situ organisms and did not collect any specimens.

Note that full information on the approval of the study protocol must also be provided in the manuscript.

Plants

Seed stocks	N/A
Novel plant genotypes	N/A
Authentication	N/A
